# Sea level controls on Ediacaran-Cambrian animal radiations

**DOI:** 10.1126/sciadv.ado6462

**Published:** 2024-07-31

**Authors:** Fred T. Bowyer, Rachel A. Wood, Mariana Yilales

**Affiliations:** School of GeoSciences, University of Edinburgh, James Hutton Road, Edinburgh EH9 3FE, UK.

## Abstract

The drivers of Ediacaran-Cambrian metazoan radiations remain unclear, as does the fidelity of the record. We use a global age framework [580–510 million years (Ma) ago] to estimate changes in marine sedimentary rock volume and area, reconstructed biodiversity (mean genus richness), and sampling intensity, integrated with carbonate carbon isotopes (δ^13^C_carb_) and global redox data [carbonate Uranium isotopes (δ^238^U_carb_)]. Sampling intensity correlates with overall mean reconstructed biodiversity >535 Ma ago, while second-order (~10–80 Ma) global transgressive-regressive cycles controlled the distribution of different marine sedimentary rocks. The temporal distribution of the Avalon assemblage is partly controlled by the temporally and spatially limited record of deep-marine siliciclastic rocks. Each successive rise of metazoan morphogroups that define the Avalon, White Sea, and Cambrian assemblages appears to coincide with global shallow marine oxygenation events at δ^13^C_carb_ maxima, which precede major sea level transgressions. While the record of biodiversity is biased, early metazoan radiations and oxygenation events are linked to major sea level cycles.

## Teaser

The record of Ediacaran-Cambrian biodiversity is biased, but radiations, marine oxygenation, and eustatic sea level are linked.

## INTRODUCTION

The Ediacaran-Cambrian appearance and rise of animals (metazoans) from approximately 575 to 515 Ma are seminal events in evolution. However, the underlying processes and drivers of this diversification, such as the relative influence of extrinsic, environmental factors versus intrinsic species interactions, remain unresolved. To interrogate this event, we first need to establish the global record of biodiversity itself.

The modern distribution of biodiversity is spatially highly heterogeneous, as biodiversity is distinctly regionalized, with high diversity in the tropics and a large fraction hosted in a limited number of environments or ecological communities [e.g., (*1*)]. Larger geographic areas also have higher diversities than smaller areas, known as the species-area effect (*2*). It is expected, then, that the spatial variation in biodiversity at any given moment in deep time has been shown to be greater than the temporal variation for most of the Phanerozoic fossil record of marine metazoans (*3*). Interactions between species and their environments can influence speciation and extinction rates within any given region, but over geological timescales, global diversity can also be a function of evolutionary innovation, global tectonic and climatic drivers (which may regulate global provinciality), as well as changes in atmospheric composition, biogeochemical cycling, and oceanographic circulation, although these control biodiversity via local- or regional-scale processes (*4*).

However, the fossil record of biotic diversity changes cannot be read literally. The record is notoriously incomplete and subject to many biases of sampling and preservation. The degree to which the fossil record has been explored, researched, and documented also varies, biasing the recording of data. In addition, Konservat-Lagerstätten provide exceptional windows into local biotas at episodic times and places during the history of life, and the early to middle Cambrian is famously replete with these horizons that have yielded some of the best-studied biotas of the fossil record [e.g., (*5*, *6*)]. Conditions for this exceptional preservation are created by the local dynamics of physical sedimentary deposition, as well as favorable redox profiles and early diagenetic environments, which are, in part, controlled by global seawater chemistry and oxygenation.

Changes in habitable marine shelf area, where most early metazoans lived, are controlled by relative sea level rise (transgression) and fall (regression), which, in turn, also drives the long-term geological record of marine sedimentary rocks [e.g., (*7*)], and there is a well-documented covariation between Phanerozoic metazoan macroevolutionary patterns and sedimentary rock availability [e.g., (*8*)]. This suggests that expansion of marine shelf environments coincided with the diversification of animals via the species-area effect (*2*). The late Ediacaran to Cambrian system is globally characterized by extensive continental flooding over the Great Unconformity, accompanied by a notable increase in preserved rock area/volume dominated by shallow marine settings (*9*). A strong correlation between marine sedimentary area and volume flux has been confirmed for the North American Ediacaran-Cambrian rock and metazoan fossil record, although, here, the marked rise of Cambrian reconstructed biodiversity cannot be explained by the increase in Cambrian sedimentary volume alone (*10*). These analyses suggest that there may be some secular bias in the rock record through this interval (*11*). However, while this correlation may indicate preservational biases, it also suggests that there are shared underlying driving processes that control both an increase in reconstructed biodiversity and preserved sedimentary rock quantity, such as expressed in the interrelationships between sea level, the carbon cycle, nutrient delivery, and changing oceanic redox state [e.g., see (*12*–*15*)].

**Fig. 1.**
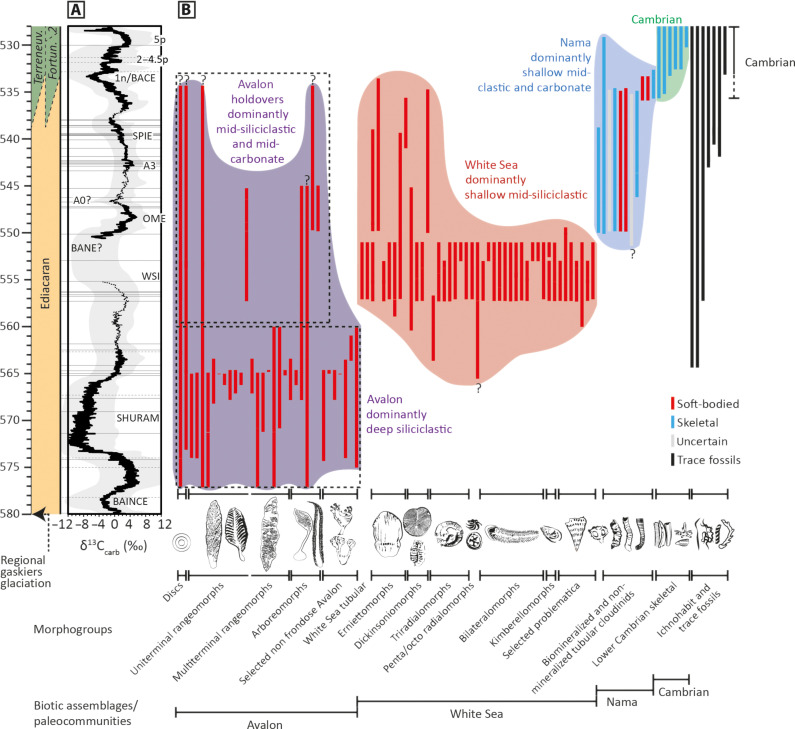
Integrated chronology and biostratigraphy of globally distributed Ediacaran to earliest Cambrian successions. (**A**) Radiometric ages (horizontal lines; table S1) calibrate the 10-point moving average of an updated global composite δ^13^C_carb_ database [data S1 and S2 updated after (*18*, *28*, *30*)]. Gray-shaded envelope shows uncertainty in the δ^13^C_carb_ framework associated with data scatter. (**B**) Compilation of estimated ranges of major Ediacaran to earliest Cambrian (580 to 528 Ma) inferred metazoan morphogroups and major trace fossil groups within the Avalon, White Sea, Nama, and Cambrian assemblages, showing dominant sedimentary setting of host successions. Age ranges, uncertainties, and full references for biostratigraphic database are provided in data S1. Modified from (*18*, *24*). Question marks denote taxa with the most uncertain maximum/minimum age constraints. See fig. S2 for an expanded version of this figure. SPIE, Spitskop excursion; OME, Omkyk excursion; WSI, White Sea Interval; BAINCE, Baiguoyuan negative carbon isotope excursion.

Although the distribution of fossil metazoan taxa is highly variable temporally and spatially (*4*, *16*–*18*), the Ediacaran-Cambrian transition is commonly viewed as comprising a succession of four metazoan radiations (Fig. 1). The Ediacara biota (~575 to ≤538 Ma) may include possible nonmetazoans [e.g., (*19*)] and stem-group and early crown-group eumetazoans [e.g., (*20*)]. The bilaterian body plan was probably present by ~560 Ma, followed by a marked increase in bilaterian fossils in the early Cambrian (the Cambrian “Explosion”) by which time most phyla had appeared (*21*). Phanerozoic evolutionary faunas are broadly defined by high-level taxonomic groups and display a broadly synchronous logarithmic increase in reconstructed biodiversity followed by extinctions, with the increase in diversity associated with successive faunas coinciding with a decline of the previously dominant fauna (*8*). The Ediacara biota has been similarly subdivided into three temporally overlapping assemblages (or paleocommunities) (Fig. 1B). These, however, are thought to have occupied different sedimentary settings: The soft-bodied Avalon assemblage (rangeomorphs, arboreomorphs, and nonfrondose morphogroups) initially occupied deeper marine siliciclastic settings, but representatives are also found in mid-depth carbonate and siliciclastic settings <560 Ma; the soft-bodied White Sea assemblage (erniettomorphs, dickinsoniomorphs, triradialomorphs, penta/octoradialomorphs, bilateralomorphs, kimberellomorphs, and problematica) occupied shallow to mid-depth siliciclastic marine settings; and the soft-bodied and skeletal (calcified) Nama paleocommunity (dominated by cloudinomorphs) is found in dominantly shallow marine siliciclastic and carbonate settings (*16*, *17*, *22*–*24*).

Apparent extinctions, as well as possible ecological shifts, are suggested to have shaped early metazoan evolutionary dynamics (*17*, *25*). The Avalon assemblage appears very broadly coincident with positive δ^13^C_carb_ values that predate the onset of the Shuram δ^13^C_carb_ excursion [Fig. 1; e.g., (*26*)]. Both the Avalon and White Sea paleocommunities largely disappeared by ~550.5 Ma (*25*, *27*, *28*), but the causes are still unclear, largely because of uncertainties in global chemostratigraphic correlation between approximately 560 and 550 Ma. The first calcified metazoans appear ~550.5 Ma in shallow marine carbonate rocks globally (*18*, *29*) and increase in diversity substantially in the Cambrian, mainly as small shelly fossils (SSFs) found within shallow marine carbonates. The Ediacaran-Cambrian boundary, defined by the first appearance of the ichnospecies *Treptichnus pedum* and the lowest level of the associated *T. pedum* ichnozone, has been suggested to coincide with, or immediately postdate, the nadir of the “Basal Cambrian negative carbon isotope (δ^13^C) excursion” (BACE), but both the precise position of this first appearance relative to the BACE nadir and the age of onset of the BACE (~538 to 535 Ma) remain uncertain (*18*, *30*). This is due, in part, to a global paucity of continuous Ediacaran-Cambrian successions. The disappearance of the Ediacara biota and subsequent radiation of the Cambrian biota have also been hypothesized to result from the decline of unique taphonomic preservational modes, an absence of outcrops of appropriate sedimentary settings, or a mass extinction [e.g., (*31*, *32*)]. This biotic transition broadly coincides with the BACE, which marks a global perturbation of the marine carbon cycle [e.g., see (*30*, *33*–*35*)], but the relationship of this to any potential extinction mechanism, such as a rise in shallow marine anoxia, is not established. The four Ediacaran to early Cambrian paleocommunities therefore differ in their preferred depositional settings, and so our ability to confidently track evolutionary change over time may be hampered by the uneven or potentially biased temporal and spatial distribution of their respective rock records.

Calibrated molecular clock studies show that the origin of the Metazoa, the last common eumetazoan ancestor, the protostome-deuterostome ancestor, and all bilaterian clades predate the first appearance of macroscopic representatives in the fossil record (*36*). This is not only due to issues of preservation, sampling, and acquisition of taxonomically identifiable features but also suggests that developmental and morphological novelties, as well as the unique characters that define the origin of major metazoan groups, may be decoupled from the drivers that allowed them to gain large body size, proliferate, and hence rise to ecological prominence in the fossil record, i.e., features that promote the expression as radiations in the fossil record. Intervals when metazoans dominate and radiate therefore often record the operation of ecological or extrinsic drivers (*36*).

Evolutionary innovations can also create dynamic feedbacks of species interactions, as they may act as ecosystem engineers and alter the flow and structural pathways of energy through the biosphere (*4*). The diversification of bilaterian animal phyla in the Cambrian must have increased metazoan abundance and fueled interspecific competition, thereby further facilitating biodiversification and biomineralization and promoting the rise of more energy intensive ecologies, such as burrowing and predation, leading to escalatory arms races [e.g., see (*37*)]. Some have further suggested that the early radiation of bilaterians may have caused the extinction of the Ediacaran biota by slow biotic replacement [see review in (*32*)].

Changes in global shallow marine oxygen levels during the Ediacaran-Cambrian interval have been inferred on the basis of interpretations of paleoredox proxy data, including uranium isotopes (δ^238^U). Seawater δ^238^U compilations from both carbonate and siliciclastic rocks suggest that multiple oscillations in global marine redox conditions occurred at 1- to 10-Ma timescales [e.g., (*38*–*40*)]. Available geochemical data therefore support recent modeling efforts (*41*), which show that any increase in environmental oxygen levels during the Ediacaran-Cambrian interval did not proceed unidirectionally but rather via a series of oxygenation pulses that likely correspond with atmospheric and “oceanic oxygenation events” [OOEs; e.g., (*42*)]. These OOEs would likely have been accompanied by corresponding regional and/or global changes in nutrient and productivity regimes and may have been driven, in part, by changing weathering dynamics, including changes in the composition of weathering material [e.g., (*42*, *43*)].

The assumption that δ^238^U data can be used as a proxy to track changes in global open ocean paleoredox conditions has been challenged by studies, which show that sea level changes [e.g., (*44*)], as well as local productivity and basin restriction [e.g., (*45*)], also control δ^238^U variability in the sedimentary record. In particular, a decline in δ^238^U has been noted to coincide with local relative sea level fall and changes in primary carbonate mineralogy and diagenesis (*44*), and the offset in δ^238^U between seawater and mudstone records has been shown to be sensitive to basin hydrography, organic carbon export dynamics, and changes in sedimentation rate and lithology (*45*). Careful screening of geochemical data, however, combined with an integrated multiproxy approach that considers sea level change, regional basin dynamics and associated degrees of restriction, and global chronostratigraphic constraints and associated uncertainties, has the potential to reveal redox-related trends. Integrating δ^238^U_carb_ and carbonate-associated sulfate (δ^34^S_CAS_) isotopic data reveals that supposed OOEs in the Ediacaran-Cambrian interval occur synchronously with changes to the global marine carbon cycle, as indicated by co-occurring trends in δ^13^C_carb_ data from the same samples, in addition to trends in coeval data revealed by regional and global chemostratigraphic correlation (*39*, *46*, *47*). This implies that broad trends in data compilations of multi-isotope systems (e.g., δ^13^C_carb_─δ^238^U_carb_─δ^34^S_CAS_) track a common process, which may reflect the extent of anoxic seafloor that coincides with high SO_4_ reduction rates and pyrite and organic carbon burial rates. In this model, the onset of decreasing δ^13^C_carb_ and δ^34^S_CAS_ has been interpreted to reflect the onset of shallow marine oxygenation (*46*). Decreasing δ^13^C_carb_ and δ^34^S_CAS_ (falling limbs) reflects progressive oxygenation of the deeper water column, resulting in decreasing organic carbon and pyrite burial, which is consistent with coeval increases in baseline δ^238^U_carb_ data. This decreased reductant burial would gradually slow/stall atmospheric oxygenation (*46*). Increasing δ^13^C_carb_ and δ^34^S_CAS_ (rising limbs) and δ^238^U_carb_ falling limbs then captures marine deoxygenation conducive to the progressive burial of organic carbon and pyrite, driving a long-term build-up of atmospheric oxygen (*39*, *46*). Falling and rising “limbs” of the C─S─U isotope systems across this time interval may thereby reflect the balance between marine and atmospheric oxygen dynamics that control, and are controlled by, the relative areal extent of marine conditions conducive to the burial of organic carbon and pyrite (*48*). The area of seafloor available to host organic carbon and pyrite burial in productive regions of the open ocean, in turn, should be controlled by changes in relative sea level and productivity, both of which are also influenced, to some degree, by global plate reorganization and associated mid-ocean ridge length, eustatic sea level change, and prevailing regional and global climate and associated weathering regimes (*43*). Regional differences in absolute magnitude of isotopic data (e.g., δ^13^C_carb_) during globally recognized excursions have also been suggested to reflect regional depositional and early diagenetic regimes [e.g., (*49*)], and alternative drivers for Neoproterozoic δ^13^C_carb_ negative excursions have also been proposed to reflect changes in the areal extent of shallow marine carbonate settings conducive to photosynthetic primary production and local isotopic reservoir effects [e.g., (*50*)]. Each of these mechanisms is strongly influenced by eustatic and relative sea level change.

A causal relationship between shallow marine oxygenation and the Cambrian radiation has long been proposed [e.g., (*51*)], but it remains unclear as to how rising oxygen availability, as well as possible associated changes in nutrient regimes, may have promoted early animal radiations. Increasing atmospheric oxygen concentration may have deepened the redoxcline, so extending habitable water depths, promoted animal-sediment mixing [e.g., (*52*)], and allowed for the evolution of more metabolically costly ecologies such as carnivory, in turn driving a predator-prey “arms race” and hence diversification (*53*). Spatially and temporally dynamic shallow marine anoxia itself might have formed physical barriers to dispersal, thereby promoting reproductive isolation and speciation (*54*, *55*). This is inferred from the early Cambrian record of the Siberian Platform, which shows that phases of increased diversity of reef biota coincide with a deepening of the habitable zone on the shallow marine shelf which, in turn, is interpreted to correspond with a possible deepening of the redoxcline (*56*). These phases also coincide with increased speciation during local sea level lowstands, and it is suggested that lowstand intervals permitted extensive oxygenation of shallow waters over the entire craton, thereby providing oxic corridors for dispersal and the creation of new founder communities (*55*).

**Fig. 2.**
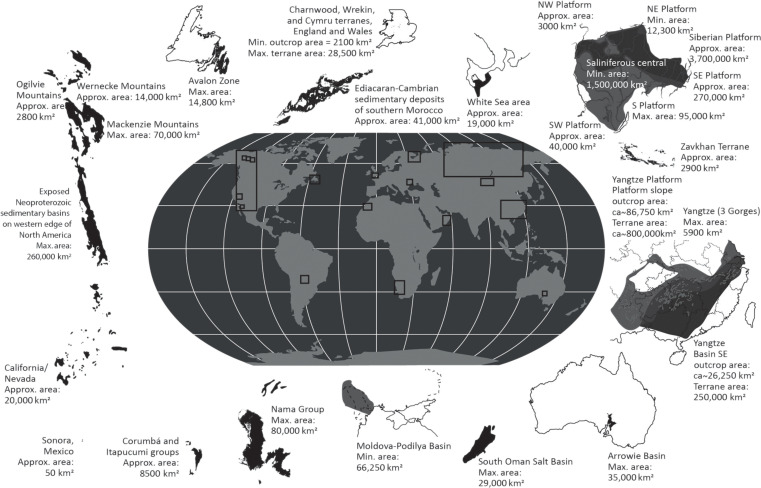
Terrane/outcrop area (maximum/minimum/approximate) calculated for each region investigated. See table S2 and data S3 for full data.

**Fig. 3.**
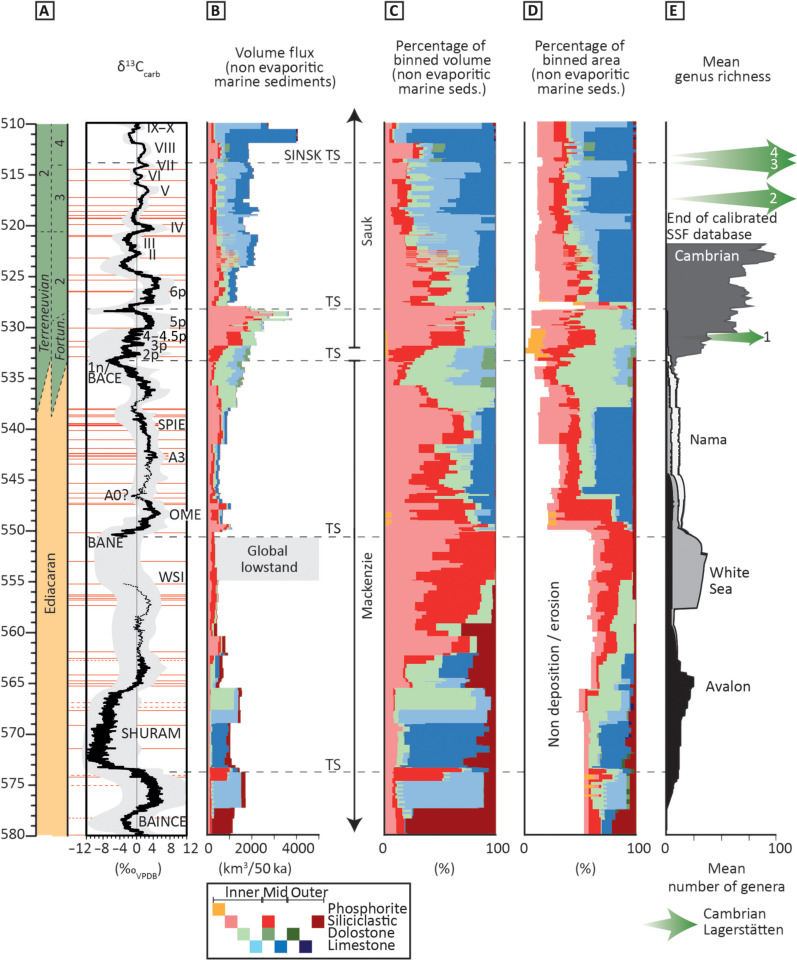
Compilation of trends in marine sedimentary rocks and hiatuses from 580 to 510 Ma. (**A**) Radiometric ages (horizontal red lines; table S1) calibrate the 10-point moving average of an updated global composite δ^13^C_carb_ curve, with associated uncertainty [data S1 and S2 updated after (*18*, *28*, *30*)]. VPDB, Vienna Pee Dee belemnite. (**B**) Estimated volume flux of globally distributed marine sedimentary rocks (table S2 and data S3). (**C**) Percentage of total volume of nonevaporitic marine sedimentary rocks (in each 50-ka bin). TS, transgressive surface. (**D**) Percentage of total area of nonevaporitic marine sedimentary rocks, including proportion of record represented by nondeposition or erosion (in each 50-ka bin). (**E**) Reconstructed biodiversity (mean genus richness) organized into four successive assemblages based on constituent morphogroups, after (*24*). Example Cambrian Lagerstätten: 1, Kuanchuanpu; 2, Chengjiang; 3, Sirius Passet; 4, Sinsk and Emu Bay Shale.

A paucity of globally distributed radiometric constraints due to limited datable ash beds, and numerous biostratigraphic correlation uncertainties, have hindered evaluation of metazoan distribution, sedimentary rock volume, and environmental change throughout the Ediacaran-Cambrian interval. Here, we use a global age framework for the interval from 580 to 510 Ma interval, that temporally calibrates available data from 24 regional composite successions (Figs. 2 and 3 and fig. S1). The fully integrated age framework construction considers all available high-precision radiometric ages, δ^13^C_carb_ chemostratigraphy, δ^238^U_carb_ data, and the biostratigraphic distribution of metazoan genera [updated after (*18*); tables S1 and S2, data S1 to S4, and figs. S1 to S6]. We use this to reconstruct mean genus richness and include all known Ediacaran Lagerstätten but exclude all early Cambrian examples up to 510 Ma from our compiled diversity data [for full list, see (*6*)], of which the most biodiverse are the Kuanchuanpu, Chengjiang, Sirius Passet, Sinsk, and Emu Bay Shale biotas (Fig. 3). Hence, our Cambrian reconstructed biodiversity data consist of the skeletal metazoan record only. This framework assumes an onset age for the BACE at ~535 Ma and an age of ~533 Ma for the first appearance of *T. pedum* after (*30*) (see Material and Methods, Figs. 1 and 3A, fig. S2, tables S1 and S2, and data S1 to S4). To reduce uncertainty, only successions where there is some confidence in temporal global correlation and spatial extent throughout the target interval are included (see the Supplementary Materials for expanded discussion and fig. S1). Hence, this record represents only a variable fraction of total global rock quantity in any one region. While absolute values of, e.g., calculated volume flux and area may therefore have limited utility, long-term temporal trends are likely to be globally meaningful and will capture underlying mechanistic processes (see data S3). These data therefore allow investigation of this interval in three ways. First, we estimate temporal changes in the global marine sedimentary rock volume and area together with the distribution of different sedimentary settings to explore how the biotic record is controlled by the availability of host rocks (see Material and Methods for definition of different sedimentary settings). Preserved sedimentary volume flux (area × thickness) is mainly controlled by accommodation (governed by relative sea level fluctuations) and sediment supply, while area, which might theoretically be a more reliable metric of changing available habitable sea floor through time, is also governed by sea level transgression/regression. Both metrics might relate to habitability and so both are considered. We test the correlation between the reconstructed biodiversity, rock volume/area, and sampling intensity, using Spearman’s rank correlation (which tests for monotonic linear and nonlinear relationships). Second, we investigate the potential mechanism of long-term sea level change as a driver for observed carbon cycle dynamics and environmental oxygenation using the calibrated records of δ^13^C_carb_ and δ^238^U_carb_. Third, we test the hypothesis that global redox changes controlled successive radiations and extinctions through the Ediacaran-Cambrian transition.

## RESULTS

### Changes in the marine sedimentary record

As a result of our compilation, we assembled a dataset of 24 composite, globally occurring stratigraphic sections that are composed of >180 formations and >620 rock units (representative blocks that summarize changes in dominant basin-scale lithofacies composition), of which ~80 formations and 259 units are Ediacaran (580 to 538.8 Ma) and ~100 formations and 362 units are Cambrian (538.8 to 510 Ma; Fig. 2, fig. S1, and data S3). Prioritization of composite sections within the age framework database was based on the availability of radiometric constraints [high priority given to sections that contain ash beds dated via zircon U─Pb chemical abrasion isotope dilution thermal ionization mass spectrometry (CA-ID-TIMS), or Re─Os ages from organic-rich mudrocks; table S1] and interbedded chemostratigraphically useful δ^13^C_carb_ data (data S1 and S2) and (for the Cambrian) biostratigraphically informative data (table S2 and data S1 and S2). The systematics of age framework construction follow methods outlined in (*18*, *28*) and references therein.

Volume flux estimates of preserved nonevaporitic marine sedimentary rocks reveal some clear trends (Fig. 3, B to D). Overall, rock volume flux shows a general increase from the Ediacaran to the Cambrian, but this is not monotonic (Fig. 3B). Fluxes are relatively low from 580 to ~566 Ma, decreasing further until ~550 Ma. Volume fluxes temporarily increase episodically during the interval from ~550 to 547 Ma, then decrease, and remain low until ~542 Ma, whereupon they increase progressively until 528 Ma (Fig. 3B). A sharp decrease at 528 Ma is followed by an interval of generally elevated volume flux before maximum values at ~512 Ma.

The proportion of the total area with nonevaporitic marine sedimentary rocks present increases notably at ~550 Ma (Fig. 3D; but see below). Nondeposition or erosion dominates the record from 580 to ~550 Ma, represented by an absence of preserved rock in ~50 to 60% of the total database, but the record becomes substantially more complete from approximately 550 Ma onward, whereafter nondeposition or erosion decreases to a mean of <15% in the lower Cambrian (Fig. 3D).

In contrast, the proportion by volume of the record of shallow marine siliciclastic settings (inner-outer shelf and upper slope) notably increases ~565 to ~550 Ma, decreases from 539 to 536 Ma, then increases from ~536 Ma, coincident with the onset of the BACE, and continues to increase until 528 Ma (Fig. 3C). Deep marine siliciclastic settings (lower slope to basinal) form up to ~80% of the record from approximately 579.9 to 578.5 Ma, but this proportion gradually decreases to ~10 to 30% by 565 Ma to <5% at ~560 Ma and then does not exceed 3.5% until after 510 Ma (Fig. 3C). Changes in the area occupied by different lithologies follow similar trends (Fig. 3D).

Carbonate rocks are abundant from ~577 Ma to at least ~555 Ma and after ~550.5 Ma, whereafter they progressively become a more dominant part of the record until 536 Ma and again from 527 to 510 Ma. These trends are again present in both the volume flux (Fig. 3C) and area (Fig. 3D) estimates.

The preserved rock record is not only temporally patchy but also spatially very heterogeneous. A limited number of regions record particularly large volume percentages of sedimentary rock, notably Avalonia, South China, and Siberia. In addition, only some regions record large volume percentages of sedimentary rock over key intervals of time: Avalonia dominates the deep-water siliciclastic record from approximately 575 to 555 Ma, and Avalonia, Laurentia, Australia, Siberia, and the East European Platform (Baltica) dominate the shallow- to mid-depth siliciclastic record from approximately 560 to 550 Ma (fig. S5). Sensitivity tests removing key regions from the compilation, however, still preserve all the major trends shown in total/percentage volume flux through time (fig. S4). Trends in area and percentage area flux estimates are also largely preserved but clearly show the degree of bias imposed by the temporal completeness of the Siberian Platform record alone before and after ~550 Ma (fig. S4).

Our global compilation does, however, show temporal trends in the distribution of different marine sedimentary rocks, interpreted in part to record major global second-order (~10 to 80 Ma) transgressive-regressive cycles that govern the record of shallow versus deep and siliciclastic versus carbonate rocks (Fig. 3, B to D). These are nested within the Ediacaran “Mackenzie” and Cambrian “Sauk” megasequences described from the North American rock record [e.g., (*7*, *10*)]. Major transgressive surfaces are identified at ~574 Ma (approximately coincident with the initiation of the Shuram δ^13^C_carb_ excursion), ~550.5 Ma, ~534 to 533 Ma (with an onset that is currently poorly constrained but may represent a global, diachronous sequence boundary), ~528 Ma, and ~514 Ma (Fig. 3B). Each major transgressive surface generally marks a sharp decrease, followed by a progressive increase, in sedimentary rock volume. Cambrian sections largely appear to transition from proximal (shallow inner shelf settings) to distal (mid-outer shelf and deeper marine settings) with transgression and continental flooding (fig. S1).

Low total rock volume throughout the ~565- to 550-Ma interval coincides with polar glaciations that may have extended to mid-latitudes at ~555 to 550 Ma, which is supported by glacial deposits that are radiometrically constrained on multiple cratons [e.g., (*57*–*59*)]. This therefore potentially records a protracted interval of generally low sea level, particularly during ~555 to 550 Ma, when carbonate rock volume reaches its nadir (see the Supplementary Materials for expanded discussion). Phosphorites are concentrated in condensed sections, particularly at the base of three transgressions; at ~550 Ma, coincident with and during recovery from the BACE at ~534 to 530 Ma, and at ~528 to 527 Ma.

### Biotic and sedimentary record dynamics

**Fig. 4.**
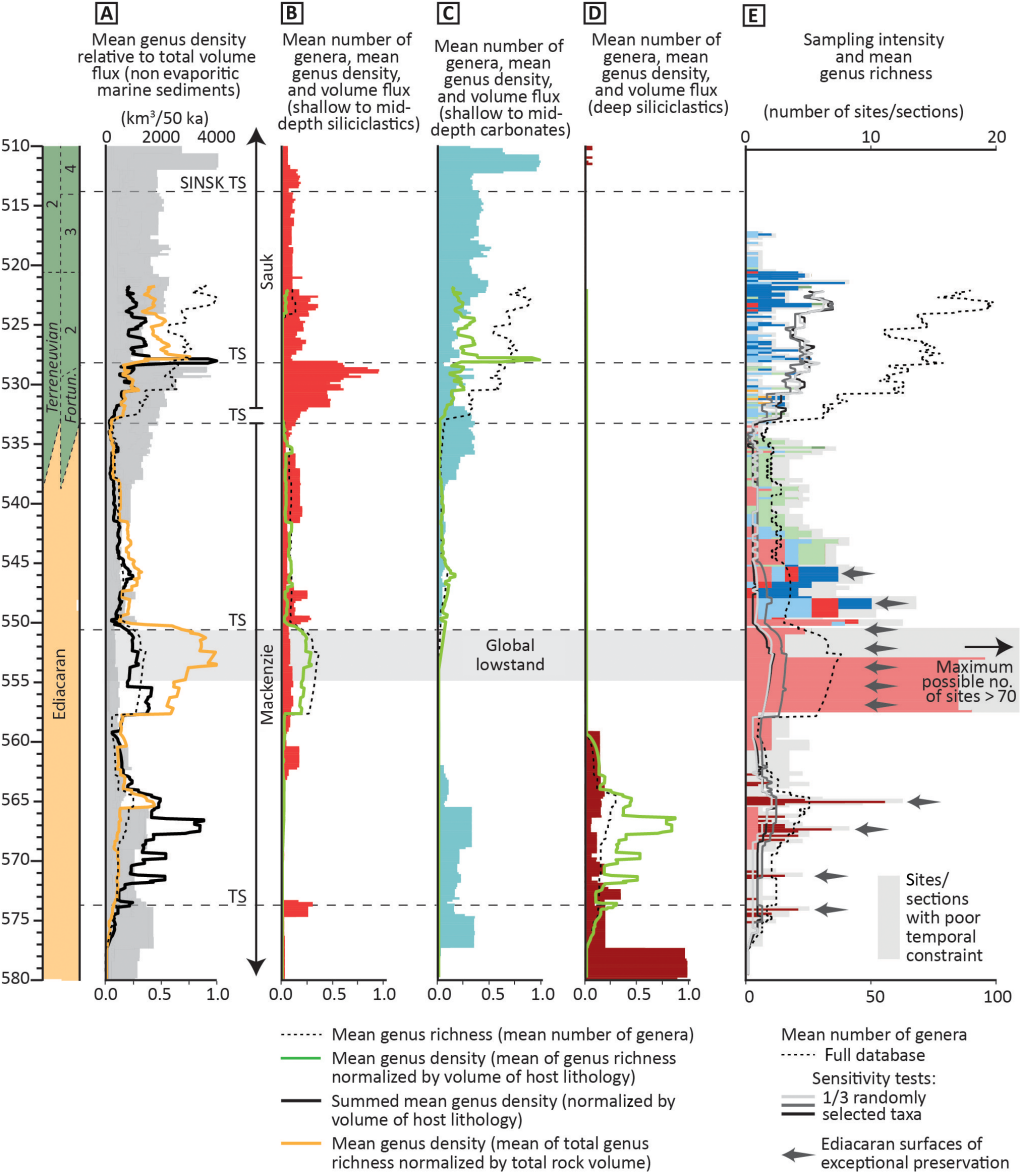
Comparison of total volume flux of marine sedimentary rocks and lithology-specific metazoan distribution from 580 to 510 Ma. (**A**) Total volume flux of marine sedimentary rocks with reconstructed biodiversity curves. (**B**) Normalized volume flux of shallow to mid-depth siliciclastic rocks and normalized distribution of associated metazoans. (**C**) Normalized volume flux of shallow to mid-depth carbonate rocks (limestone and dolostone) and normalized distribution of associated metazoans. (**D**) Normalized volume flux of deep-water siliciclastic rocks and normalized distribution of associated metazoans. (**E**) Frequency of sampling of sites/surfaces/localities (colored according to dominant lithologies, as in Fig. 3) and mean genus richness (dashed line). Gray lines each represent randomized subsamples of a third of total mean reconstructed biodiversity. Sites of exceptional preservation are noted. In (A) to (D), mean genus density is normalized to 1, and in (B) to (D), volume flux is also normalized to 1 to show relative changes in the volume flux of each sedimentary setting through time.

Our compilation considers 332 raw counts of genera, of which 81 are Ediacaran (pre-BACE onset, >535 Ma) and 251 Cambrian (post-BACE onset, <535 Ma; non-Lagerstätten, skeletal genera only). Total mean reconstructed biodiversity (mean genus richness) shows peaks during the 575- to 560-Ma and 557- to 550-Ma intervals, and a marked increase after 533 Ma (Fig. 3E). Random subsamples of this dataset also show the same trends (Fig. 4E).

When considering the complete sedimentary rock and metazoan datasets (580 to 522 Ma), no statistically significant (*P* < 0.05) correlation (Spearman’s rank, ρ) is evident between the mean generic richness and the volume of all lithologies (Table 1 and Fig. 4A). However, statistically significant correlations are expressed between the mean generic richness and the volume of hosting lithologies. There is a moderate positive correlation between the mean generic richness of carbonate-hosted biota and the volume of shallow to mid-depth carbonates, whereas there are weak and moderate negative correlations between the mean generic richness and the volume of shallow to mid-depth siliciclastics and deep siliciclastics, respectively (Table 1). First differences of the same correlation metrics yield no statistically significant correlations, with the exception of a weak negative correlation between the volume of deep siliciclastics and the mean generic richness of deep siliciclastic-hosted biotas (Table 1).

**Table 1.** Statistical correlations for reconstructed biodiversity compared to volume/area of marine lithologies and sampling intensity. Bold type indicates statistical significance (*P* values) within the 95% confidence interval.

Subset	Correlation with reconstructed biodiversity (mean number of genera)	Raw data		First difference	
		Spearman’s rank		Spearman’s rank	
		Statistical correlation (ρ)	Significance (*P* value)	Statistical correlation (ρ)	Significance (*P* value)
**All data**	Vol. all lithologies	0.05	0.244	0.047	0.266
	Vol. deep siliciclastics	−0.18	**0.004**	−0.22	**<0.001**
	Vol. shallow to mid-depth siliciclastics	−0.14	**<0.001**	−0.05	0.205
	Vol. shallow to mid-depth carbonates	0.39	**<2.2 × 10** ^**−16**^	−0.07	0.100
	Area all lithologies	0.21	**1.62 × 10** ^**−7**^	0.09	**0.023**
	Area deep siliciclastics	−0.17	**0.009**	0.11	0.079
	Area shallow to mid-depth siliciclastics	0.54	**<2.2 × 10** ^**−16**^	0.09	**0.037**
	Area shallow to mid-depth carbonates	0.78	**<2.2 × 10** ^**−16**^	0.03	0.480
**Pre-BACE onset Ediacaran (>535 Ma)**	**Sampling intensity**	0.78	**<2.2 × 10** ^**−16**^	–	–
	Vol. all lithologies	−0.36	**2.9 × 10** ^**−15**^	0.01	0.803
	Vol. deep siliciclastics	−0.18	**0.004**	−0.22	**<0.001**
	Vol. shallow to mid-depth siliciclastics	−0.07	0.153	−0.04	0.390
	Vol. shallow to mid-depth carbonates	−0.05	0.290	−0.08	0.092
	Area all lithologies	0.17	**<0.001**	0.13	**0.004**
	Area deep siliciclastics	−0.17	**0.009**	0.11	0.079
	Area shallow to mid-depth siliciclastics	0.63	**<2.2 × 10** ^**−16**^	0.09	**0.050**
	Area shallow to mid-depth carbonates	0.57	**<2.2 × 10** ^**−16**^	0.10	**0.036**
**Post-BACE onset “Cambrian” (<535 Ma)**	**Sampling intensity**	0.15	0.090		
	Vol. all lithologies	0.00	0.979	0.12	0.191
	Vol. deep siliciclastics	–	–	–	–
	Vol. shallow to mid-depth siliciclastics	−0.04	**1.31 × 10** ^**−7**^	0.01	0.931
	Vol. shallow to mid-depth carbonates	0.14	0.104	−0.05	0.561
	Area all lithologies	−0.10	0.612	0.01	0.942
	Area deep siliciclastics	–	–	–	–
	Area shallow to mid-depth siliciclastics	−0.25	**0.005**	−0.03	0.705
	Area shallow to mid-depth carbonates	0.09	0.321	−0.05	0.608
**All data after removal of Siberian Platform**	Vol. all lithologies	0.29	**1.99 × 10** ^**−12**^	0.03	0.462
	Vol. deep siliciclastics	0.09	0.144	−0.25	**<0.001**
	Vol. shallow to mid-depth siliciclastics	0.04	0.314	−0.03	0.547
	Vol. shallow to mid-depth carbonates	0.55	**<2.2 × 10** ^**−16**^	−0.12	**0.006**
	Area all lithologies	0.43	**<2.2 × 10** ^**−16**^	0.15	**<0.001**
	Area deep siliciclastics	−0.12	0.060	0.11	0.092
	Area shallow to mid-depth siliciclastics	0.65	**<2.2 × 10** ^**−16**^	0.10	**0.016**
	Area shallow to mid-depth carbonates	0.01	0.848	−0.02	0.693
**Pre-BACE onset Ediacaran (>535 Ma) after removal of Siberian Platform**	Vol. all lithologies	−0.16	**<0.001**	−0.03	0.548
	Vol. deep siliciclastics	0.09	0.144	−0.25	**<0.001**
	Vol. shallow to mid-depth siliciclastics	0.47	**<2.2 × 10** ^**−16**^	−0.05	0.302
	Vol. shallow to mid-depth carbonates	0.07	0.172	−0.17	**<0.001**
	Area all lithologies	0.41	**2.2 × 10** ^**−16**^	0.15	**0.002**
	Area deep siliciclastics	−0.12	0.060	0.11	0.092
	Area shallow to mid-depth siliciclastics	0.78	**<2.2 × 10** ^**−16**^	0.09	0.077
	Area shallow to mid-depth carbonates	−0.20	**4.81 × 10** ^**−5**^	−0.03	0.584
**Post-BACE onset Cambrian (<535 Ma) after removal of Siberian Platform**	Vol. all lithologies	0.46	**4.53 × 10** ^**−8**^	0.15	0.089
	Vol. deep siliciclastics	–	–	–	–
	Vol. shallow to mid-depth siliciclastics	−0.27	**0.002**	−0.07	0.408
	Vol. shallow to mid-depth carbonates	0.76	**<2.2 × 10** ^**−16**^	−0.01	0.870
	Area all lithologies	0.41	**1.46e** ^**−6**^	0.10	0.252
	Area deep siliciclastics	–	–	–	–
	Area shallow to mid-depth siliciclastics	−0.09	0.321	−0.06	0.467
	Area shallow to mid-depth carbonates	0.14	0.125	0.03	0.735
	**δ**^**13**^**C**_**carb**_ **/ δ**^**238**^**U**_**carb**_ **correlations**				
**580–510 Ma (*n* = 448)**	10-point moving mean / 10-point moving mean	−0.76	**<2.2 × 10** ^**−16**^	−0.36	**4.17 × 10** ^**−15**^
	10-point moving mean / 10-point moving min	−0.68	**<2.2 × 10** ^**−16**^	−0.23	**9.06 × 10** ^**−7**^
	Raw δ^13^C_carb_ / δ^238^U_carb_	−0.65	**<2.2 × 10** ^**−16**^	−0.14	**0.002**
**1560–510 Ma (*n* = 668)**	10-point moving mean / 10-point moving mean	−0.71	**<2.2 × 10** ^**−16**^	−0.29	**4.73 × 10** ^**−14**^
	10-point moving mean / 10-point moving min	−0.64	**<2.2 × 10** ^**−16**^	−0.20	**9.33 × 10** ^**−8**^
	Raw δ^13^C_carb_ / δ^238^U_carb_	−0.59	**<2.2 × 10** ^**−16**^	−0.13	**0.001**

Statistically significant correlations are also expressed between mean generic richness and total area (both in sum and subdivided into host lithologies). There is a weak-moderate positive correlation with the area of all lithologies, a weak negative correlation with the area of deep siliciclastics, and strong positive correlations with both shallow to mid-depth siliciclastic and shallow to mid-depth carbonate area (Table 1). However, first differences of lithology-specific mean generic richness only yield a weak but significant positive correlation between the area of shallow to mid-depth siliciclastics and the mean generic richness of shallow to mid-depth siliciclastic-hosted biotas.

When subdividing the data into Ediacaran (pre-BACE onset, >535 Ma) and Cambrian (post-BACE onset, <535 Ma), the Ediacaran data show a statistically significant moderate negative correlation between the mean generic richness and the volume of all lithologies, which is largely driven by a weak negative correlation between the volume of deep siliciclastics and mean generic richness of deep siliciclastic-hosted biotas (Table 1). There is no statistically significant correlation between the volume of shallow to mid-depth carbonates or shallow to mid-depth siliciclastics and the mean generic richness of their respective biotas in the Ediacaran dataset (Table 1). There are also statistically significant correlations in the Ediacaran dataset between mean generic richness and area, which mirror the correlations reported in the full dataset; weak positive correlation with area of all lithologies, weak negative correlation with deep siliciclastic area, and strong positive correlations with both shallow to mid-depth siliciclastic and shallow to mid-depth carbonate area (Table 1). The first differences of the Ediacaran dataset also support a weak negative correlation between the volume of deep siliciclastics and the mean generic richness of deep siliciclastic-hosted biotas, in addition to weak positive correlations between the mean generic richness and the area of all lithologies, the area of shallow to mid-depth siliciclastics, and the area of shallow to mid-depth carbonates. By contrast, the Cambrian dataset only shows weak but statistically significant negative correlations between the volume and area of shallow to mid-depth siliciclastics and the mean generic richness of associated biotas (Table 1). The first differences of the Cambrian dataset show no statistically significant correlations (Table 1).

Sensitivity tests indicate that the Siberian Platform alone has a notable influence on rock volume/area metrics, and so we also consider statistical correlations after removal of the Siberian Platform stratigraphic record. This results in several changes: First, all correlations between the volume or area of deep siliciclastics and the mean generic richness of deep siliciclastic-hosted biotas become nonsignificant; second, correlations between mean generic richness and volume of shallow to mid-depth siliciclastics in the full dataset and area of shallow to mid-depth siliciclastics in the Cambrian dataset become nonsignificant; and third, correlations between mean generic richness and area of shallow to mid-depth carbonates in the full dataset and Ediacaran dataset become nonsignificant (Table 1). Removing the Siberian Platform record also introduces statistically significant positive correlations between mean generic richness and volume of all lithologies in the full dataset and Cambrian dataset, between the mean generic richness and the volume of shallow to mid-depth siliciclastics in the Ediacaran dataset, and between the mean generic richness and the volume of shallow to mid-depth carbonates in the Cambrian dataset (Table 1). Last, removing this record also removes the strong positive correlation observed between the area of shallow to mid-depth carbonate and the mean generic richness in the Ediacaran dataset and replaces it with a weak negative correlation (Table 1). First differences, however, maintain statistically significant weak positive correlations between the mean generic richness and the area of all lithologies, and the area of shallow to mid-depth siliciclastics in the full dataset, and the area of all lithologies in the Ediacaran dataset but yield no other statistically significant correlations that support correlations observed in the raw data.

When the mean of total reconstructed generic richness is normalized to total volume flux of marine sedimentary rocks (orange line in Fig. 4A), peaks in the resulting mean genus density indicate deviations from trends in volume flux, and these instances are also captured in summed mean genus density (where total mean generic richness is normalized by the volume of host lithology; black line in Fig. 4A). We further consider trends in mean generic richness after normalization to the volume (Fig. 4, B to D) or area (fig. S6, C to F) of associated sedimentary settings through time, when rock-fossil collections are subdivided into shallow to mid-depth siliciclastic, shallow to mid-depth carbonate, and deep siliciclastic settings. Both the deep siliciclastic biota between ~575 Ma and 563 Ma (dominantly the older Avalon assemblage; Fig. 4D and fig. S6F) and the shallow to mid-depth siliciclastic biota between ~557 Ma and 550 Ma (dominantly the White Sea assemblage; Fig. 4B and fig. S6C) show markedly higher mean generic richness than can be explained by changes in the relative volume or area of available host rocks. Most terminal Ediacaran to early Cambrian skeletal metazoans are reported from shallow marine carbonate rocks, and there is a statistically significant positive correlation between the mean generic richness of calcified taxa and the volume of shallow to mid-depth carbonates throughout the full duration of the dataset, both before and after removal of the Siberian Platform record (Table 1). There is also a statistically significant strong positive correlation between the mean generic richness and the volume of shallow to mid-depth carbonates <535 Ma after removal of the Siberian Platform record (Table 1). However, the Cambrian skeletal assemblage shows a rise in reconstructed biodiversity that proportionally exceeds the increase in volume flux of shallow marine carbonate, and there is a notable peak in mean genus density at ~528 Ma, when considering shallow to mid-depth carbonate-hosted Cambrian biotas (Fig. 4C).

The Avalon assemblage is known mainly from deep-water siliciclastic settings, and the record of this biota is concentrated between approximately 575 and 559 Ma. Western Avalonia (eastern Newfoundland) dominates the deep-water siliciclastic record during this interval (fig. S5). However, both correlation statistics (Table 1) and temporal trends (Fig. 4D and fig. S6F) indicate that peaks in mean reconstructed biodiversity of the Avalon assemblage are not controlled by corresponding peaks in the availability (volume or area) of deep siliciclastics.

The record of shallow to mid-depth siliciclastic rocks that host the majority of the White Sea assemblage is also spatially very patchy during the White Sea interval of ~560 to 550 Ma (fig. S5), but Australia and the East European Platform sedimentary rocks house most of this diversity (data S1). Correlation statistics show that there are statistically significant positive correlations between mean reconstructed biodiversity and shallow to mid-depth siliciclastic volume (after removal of the Siberian Platform dataset) and area (before and after removal of the Siberian Platform dataset) in the Ediacaran (Table 1), which may suggest that rock availability does impart some influence on mean generic richness of soft-bodied fossils in shallow to mid-depth siliciclastics across the White Sea and Nama assemblage interval.

### Sampling and the biotic record

**Fig. 5.**
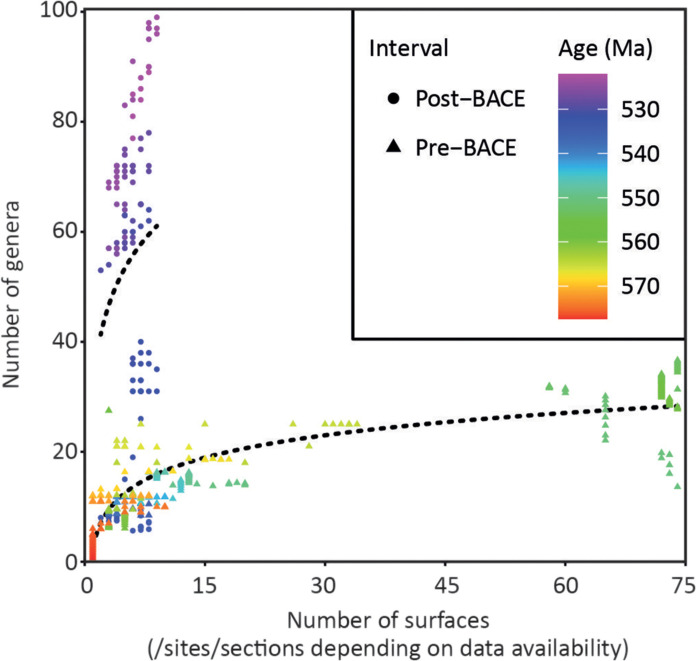
Correlation between sampling intensity and reconstructed biodiversity from 580 to 510 Ma.

The sampling of fossils through time is far from uniform, with peaks of intensively sampled surfaces of exceptional preservation corresponding to, e.g., the Mistaken Point and Trepassey formations of Newfoundland (~565 Ma) and the Siberian Platform of Russia, Yangtze Platform of South China, and Zavkhan Terrane of Mongolia (~534 to 520 Ma) following peaks of mean reconstructed diversity (Fig. 4E). Exceptionally fossiliferous bedding planes in sections of the White Sea Winter Coast of Russia and the Ediacara Member of Australia (~557 to 550 Ma) have also been intensively sampled, but changes in relative sampling intensity throughout successions in this interval are difficult to estimate using our age framework due to the paucity of radiometrically dated levels, carbonate interbeds that provide δ^13^C_carb_ data, or knowledge of the precise levels of sampled bedding planes in composite sections. Consequently, only maximum possible sampling intensity is presented for the ~557- to 550-Ma interval (Fig. 4E) on the basis of the maximum possible number of co-occurring fossiliferous bedding planes in both regions [using collections listed in the database of (*17*)] in 100-ka time bins and the interpreted biostratigraphic correlation between White Sea and Australian sections. By contrast, the 549.5- to 534-Ma interval appears to show a decrease in sampling intensity that correlates with a decrease in mean reconstructed biodiversity of the Nama assemblage. Sampling then markedly increases from 534 to 518 Ma, in concert with rising mean reconstructed diversity of the Cambrian biota, although this follows a different multiple. After ~518 Ma, numerous, but temporally episodic, Lagerstätten (not included in this database) result in a marked increase in reconstructed biodiversity due to exceptional preservation [e.g., the Maotianshan shales (*60*)]. There is a strong and statistically significant positive correlation between total mean reconstructed biodiversity and sampling intensity pre-BACE onset (>535 Ma) but no statistically significant correlation between these metrics post-BACE onset (<535 Ma; Fig. 5 and Table 1). In the post-BACE interval, sampling intensity remains fairly constant despite the increase in reconstructed biodiversity through time.

### Evolutionary dynamics

**Fig. 6.**
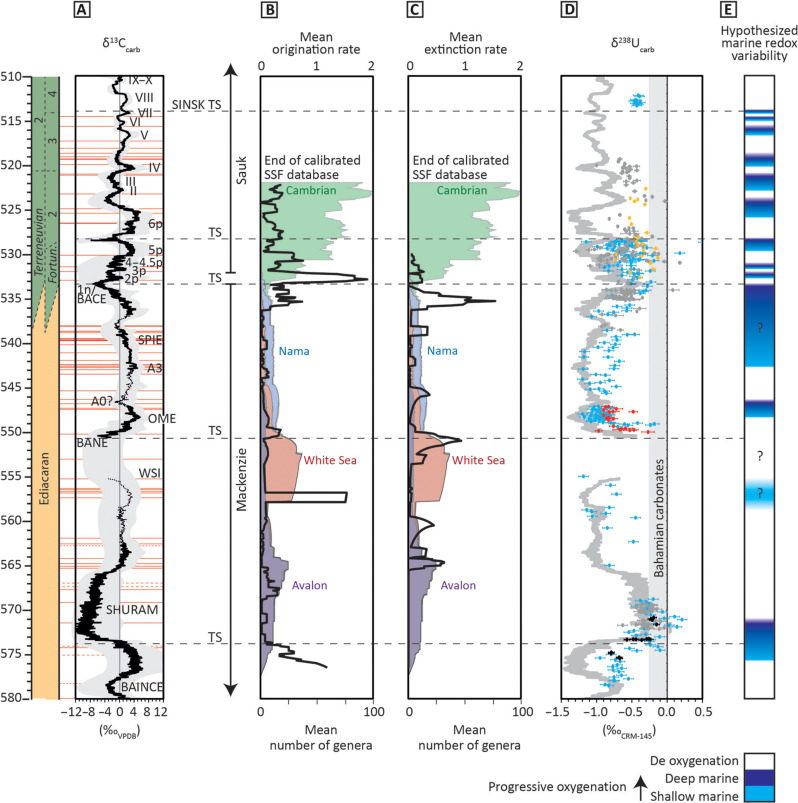
Compiled volume flux of globally distributed marine sedimentary successions, mean rates of origination and extinction, and global redox data. (**A**) Updated global composite δ^13^C_carb_ curve with uncertainty [updated after (*18*, *28*, *30*)] calibrated to radiometric dates (red horizontal lines; table S1). Mean rates of (**B**) origination and (**C**) extinction with reconstructed biodiversity (mean genus richness) of the four successive assemblages based on constituent morphogroups, after (*24*). (**D**) Temporally calibrated carbonate U isotope (δ^238^U_carb_) data from multiple sources. Data are color-coded on the basis of provenance (see data S1 for full data and references). The oscillating pale gray line in (**D**) shows an inverted representation of the 10-point moving average of global δ^13^C_carb_ data (A), used as a visual aid to show how a perfect anticorrelation between δ^13^C_carb_ and the most reliable δ^238^U_carb_ data would appear. (**E**) Hypothesized marine redox variability based on interpretation of δ^13^C_carb_ and δ^238^U_carb_ records.

While Ediacaran reconstructed biodiversity can be shown to be driven, in part, by sampling, the appearances of new morphogroups that define the evolutionary assemblages coincide with intervals of elevated rates of origination (Fig. 6B). Apart from the first appearance of metazoans of the Avalon assemblage (~575 Ma), mean origination rates show peaks at ~557 Ma coincident with the appearance of new morphogroups of the White Sea assemblage (e.g., erniettomorphs, kimberellomorphs, and problematica) and from 535 to 532 Ma coincident with the BACE interval that records the appearance of new small shelly taxa of the Cambrian paleocommunity (Fig. 6B).

Elevated mean extinction rates occur particularly at 552 to 551 Ma (Fig. 6C), which marks the disappearance of triradialomorphs, penta/octoradialomorphs, numerous bilateralomorphs, and kimberellomorphs that distinguish the White Sea assemblage, and at ~536 to 534 Ma, recording the last Nama assemblage taxa coincident with the BACE onset (Fig. 6C). The BACE therefore appears to represent a rapid paired extinction/origination event near the base of a major second-order transgression, immediately before, or coincident with, the start of the Sauk megasequence (Fig. 6, B and C).

**Fig. 7.**
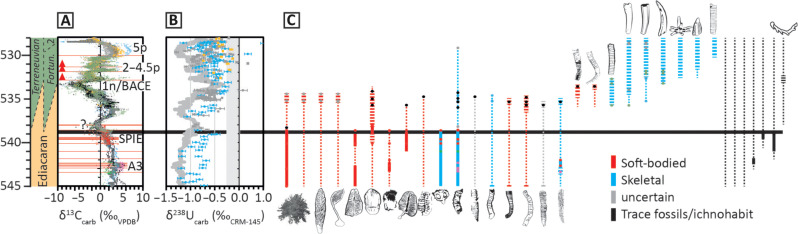
Temporally calibrated global redox and biodiversity across the pre-BACE to post-BACE interval, 545 to 528 Ma. (**A**) Global composite δ^13^C_carb_ framework [data S1 and S2 updated after (*18*, *28*, *30*)] calibrated to radiometric dates (red horizontal lines; table S1). (**B**) Temporally calibrated carbonate U isotope data (δ^238^U_carb_; data S1 and S2), with oscillating pale gray line showing an inverted representation of the 10-point moving average of global δ^13^C_carb_ data (A), corresponding to the trend, followed by minimum δ^238^U_carb_ values. (**C**) Biostratigraphic ranges of global macrofossil occurrences calibrated within this age framework (data S1 and S2). Thick black horizontal lime shows the current base Cambrian. See fig. S2 for full expanded version of this figure and key to vertical range and fossil symbols. Data in (A) and (B) are colored according to provenance (full data and references provided in data S1).

The 536- to 534-Ma (BACE) interval shows, however, very low mean generic richness in our compilation (Fig. 3E). Probable metazoans that continue close to the Ediacaran-Cambrian boundary, or around the inferred position of the BACE, consist of only two to four soft-bodied genera of the Ediacaran biota (including the erniettomorphs *Ernietta* and *Swartpuntia*), *Nenoxites* (*Shaanxilithes* in South China), four skeletal cloudinids (*Cloudina*, *Sinotubulites*, *Corumbella*, and *Zuunia*), and up to six nonskeletal (or uncertain) cloudinids (*Sekwitubulus*, *Conotubus*, *Gaojiashania*, *Wutubus*, *Saarina*, and *Costatubus*) (Fig. 7C). The pre-BACE onset appearance of treptichnid traces (e.g., in the Urusis Formation of the Nama Group) (*61*–*63*), a transitionary Ediacaran-Cambrian biotic assemblage consisting of protoconodonts, anabaritids, and cloudinids in carbonate-dominated settings (e.g., Zavkhan Terrane and Mongolia) (*64*), and typical Ediacaran soft-bodied fossils and cloudinids in mixed siliciclastic-carbonate settings (e.g., the Great Basin and Laurentia) (*65*) all predate the lowest chemostratigraphically constrained occurrence of *T. pedum* (*18*, *30*)*.*

### Paleoredox, sea level and biotic records

Although δ^238^U_carb_ data are sparse in some intervals and not all are temporally well-constrained throughout the record, as previously noted (*39*), there is a clear and statistically significant (Table 1) systematic antithetic relationship (negative correlation) between multiple and globally synchronous marine (δ^13^C_carb_ and δ^238^U_carb_) records. This is expressed as large, negative δ^238^U_carb_ excursions (of magnitude up to approximately −0.6‰) correlated with positive δ^13^C_carb_ excursions (Fig. 5E), with co-occurring trends that appear to support the view that OOEs oscillated synchronously with changes to the global marine carbon cycle. When interpreting long-term trends in combined δ^13^C_carb_ and δ^238^U_carb_ data as a result of underlying paleoredox-related mechanisms, even where data are patchy, at least 10 possible oxic-anoxic intervals are distinguished, and baseline δ^238^U_carb_ values become, on average, more positive, and oscillations become shorter throughout the interval.

A notable and prolonged OOE at ~575 to 569 Ma, when δ^238^U_carb_ values increase to approach modern seawater composition, coincides with the appearance and rise of the Avalon assemblage. The onset of this OOE (corresponding with the pre-Shuram δ^13^C_carb_ peak) immediately predates the onset of a major transgression at ~574 Ma (Fig. 6, B and E [e.g., (*50*)]. δ^238^U_carb_ data are scarce through the ~569- to 551-Ma interval, coincident with the dominant White Sea assemblage, and δ^13^C_carb_ data are particularly sparse or entirely absent throughout the ~555- to 551-Ma interval. However, on the basis of the available data, the areal extent of reducing marine conditions may have increased between 565 to 557 Ma, potentially coincident with late Ediacaran high to mid-latitude regional glaciations [e.g., (*57*–*59*)]. A second major shallow marine oxygenation event can then be inferred on the basis of peak δ^13^C_carb_ values at ~557 Ma, with subsequent oxygenation of deeper environments culminating in maximum oxygenated seafloor area at peak δ^238^U_carb_ values recorded during the nadir of the subsequent negative δ^13^C_carb_ excursion (BANE) at ~551 to 550 Ma. This interval broadly coincides with the disappearance of the White Sea assemblage, the appearance of the Nama assemblage, and a major transgression at or immediately after ~550.5 Ma. While this transgression is expressed in globally distributed successions, it is most pronounced in the lower Nama Group, Namibia, where active basin subsidence associated with the ongoing Damara orogeny also initiated rapid relative sea level rise. This underscores the long-recognized necessity of interrogating regional tectonic models of basin subsidence in concert with global frameworks that constrain the temporal co-occurrence of transgressive deposition to recognize eustatic sea level change. The total duration of the subsequent interval of expanding anoxia is uncertain but may extend from 550.5 to ≤543 Ma and is coincident with the dominant Nama assemblage (*40*). A cryptic OOE may also occur during the decline in δ^13^C_carb_ at ~540 to 538 Ma; however, this interval currently suffers from uncertainty in the global age framework associated with the termination of carbonate deposition in the Nama Group, Namibia, and uncertain global chemostratigraphic correlation. OOEs thereafter occur during the falling limb of the BACE at ~536 to 534 Ma (Fig. 7B) and subsequently with the falling limbs of successive δ^13^C excursions 2p to 4.5p, 5p at ~529.5 to 528 Ma, and 6p at ~527 to 524 Ma [although δ^238^U_carb_ data for peak 6p are sparse because of the recalibration of data from the Dahai Member of the Zhujiaqing Formation, South China, to 5p after (*66*)]. Subsequent pulses of shallow marine oxygenation at peak δ^13^C_carb_ and during the falling limbs of peaks II to III, IV, V, VI, and VII until ~515 Ma are largely based on trends in δ^13^C_carb_ and δ^34^S_CAS_ on the Siberian Platform, following the interpretations of (*46*). Transgressive surfaces at ~574, ~550.5, ~534 to 533, ~528, and ~514 Ma therefore postdate δ^13^C_carb_ maxima and often coincide with δ^13^C_carb_ minima, approximating the pivot from inferred maximum oxygenated seafloor area (corresponding with the minimum rate of increase in atmospheric oxygen concentration) to decreasing areal extent of oxygenated seafloor, increasing reductant burial, and increasing rate of atmospheric oxygenation.

## DISCUSSION

### Trends in the global Ediacaran-Cambrian sedimentary rock record

Our sedimentary rock record estimates are self-consistent and show a good match with trends derived from North American compilations that extract more precise volume estimates from the Macrostrat database (*10*, *67*). This is despite the fact that North American–only compilations incorporate all sedimentary successions, including those without confident temporal constraint, and use a different age model. While these compilations produce different absolute values, they still show a broadly similar increase in volume flux through the same time interval as our global compilation, as well as an increase in the proportion of carbonate rocks at ~577 to 555 Ma and a short-lived increase in overall sedimentary rock volume at ~550 to 545 Ma (*10*, *67*). While trends in our compilation may be largely representative of the global late Ediacaran record at ~580 to 538 Ma, there is a relative paucity of preserved rock outcrop/volume during this interval. By comparison, our dataset may be less globally representative of the Cambrian rock record, given the notable increase in preserved Cambrian sedimentary rock volume.

Our volume flux estimates of preserved marine sedimentary rock vary in their accuracy due to variable outcrop exposure, availability and resolution of published geological maps, variability in dip of strata, and some poorly constrained unit thicknesses (full details in table S2). With the exception of the Nama Group, Namibia, and because of the current absence of available databases that are able to accurately interrogate rock area polygons outside of the North American Macrostrat database, we also make no attempt to vary prescribed area estimates based on individual rock units throughout a given composite section. Moreover, there is some subjectivity in water depth assignments, given that they are based on published literature rather than a single individual’s field-based analysis [but see the approach used in (*16*) and used herein to minimize bias by considering available published sedimentological indicators].

While global coverage of preserved marine sediment is spatially patchy, trends in area and percentage area flux estimates are largely preserved. However, we show that some sections contribute sizable proportions of the rock quantities and drive some observed trends (fig. S4). Notably, it is the temporal completeness of the Siberian Platform record alone that drives the marked increase in area and percentage area at ~550 Ma, which is largely a result of the inception of widespread deposition across large areas of the Siberian Platform and the subsequent preservation of this record on the cratonic interior (fig. S4). Eastern Newfoundland alone also dominates the deep-water siliciclastic record that hosts the Avalon assemblage in the ~575- to 559-Ma interval, and Australia and the White Sea shallow to mid-depth siliciclastic rocks provide most of our record of the White Sea assemblage.

The age model used here allows for a 1-Ma resolution, and this high resolution enables consideration of the coincidence of Sloss-scale transgressive relationships with other variables, such as redox evolution and reconstructed biodiversity. There remain, however, uncertainties with the age model that require focused future efforts to resolve. While the interval from approximately 555 to 550 Ma has the greatest uncertainty in global δ^13^C_carb_ age frameworks (see the Supplementary Materials for expanded discussion), the solution represented here shows that it is possible for carbonate rocks to be entirely absent from all compiled composite successions at ~555 to 550.5 Ma (Fig. 3, C and D). This age model is currently permissible as radiometric data that could constrain global δ^13^C_carb_ are conspicuously absent between approximately 555 and 550 Ma from all carbonate-dominated successions, and major sequence boundaries or erosional surfaces are ubiquitous from <555 Ma [e.g., (*68*)] to approximately >550.5 Ma (*28*) in radiometrically dated carbonate-dominated successions.

Global correlation in the ~538- to 535-Ma interval also remains uncertain, and this may be due, in part, to either widespread erosion and removal (or partial removal) of carbonate strata or the transition from carbonate to siliciclastic-dominated depositional regimes, as evidenced by the variable completeness of the BACE in numerous globally distributed sections (e.g., Risky Formation, NW Canada, and upper Turkut Formation, northern Siberia; see table S2 and references therein). This preservational variability is often region specific but can be explained by a variety of factors including global sea level fall outpacing regional basin subsidence, cratonic collision, and basin infill.

Notwithstanding these issues, our data compilation captures the Ediacaran Mackenzie and Cambrian Sauk megasequences (*7*, *10*), each of which follows a transition from proximal (e.g., shallow inner shelf settings) to distal (e.g., deeper marine shelf and mid-outer shelf) with transgression and continental flooding. Each major transgressive surface generally marks a sharp decrease, followed by a progressive increase, in sedimentary rock volume.

### Controls on reconstructed biodiversity metrics

Our compilations of sedimentary rock volume and area, and corresponding correlation statistics that test volume and area relationships against changes in mean generic richness, are markedly affected by the inclusion of the Siberian Platform record (Table 1 and fig. S4). However, some statistically significant correlations are present regardless of whether the Siberian Platform is included. First, when considering the entire time interval, statistical tests reveal significant positive correlations between mean reconstructed biodiversity and the volume of shallow to mid-depth carbonates, the area of all lithologies, and the area of shallow to mid-depth siliciclastics (Table 1). Second, a strong positive correlation is recorded between mean reconstructed biodiversity and area of shallow to mid-depth siliciclastics >535 Ma. Third, statistically significant weak-moderate negative correlations are recorded between mean reconstructed biodiversity and the volume of all lithologies >535 Ma and the volume of shallow to mid-depth siliciclastics <535 Ma. Last, while the majority of negative correlations may be associated with stochasticity (commonly supported by the paucity of corresponding negative correlations in first differences), the absence of any positive correlation between either volume or area of deep siliciclastics and mean reconstructed biodiversity of the deep siliciclastic-hosted Avalon assemblage biota suggests that rock availability alone cannot explain changes in mean reconstructed biodiversity of the Avalon assemblage.

Normalization of mean generic richness to either total sedimentary rock volume flux (Fig. 4) or total area (fig. S6) reveals peaks in genus density in discrete intervals of the record that are most likely attributed to either sampling or taphonomic biases. This is most clearly demonstrated in the Avalon assemblage interval, which is characterized by prominent peaks in genus density relative to both the volume and area of deep siliciclastics (Fig. 4D and fig. S6F). Previous analysis (*10*) has likewise shown that the increase in the number of occurrences and genera at ~570 Ma is not accompanied by an increase in sedimentary unit counts, rock area, or volume flux in Laurentia, and the subsequent volume flux increase toward ~550 Ma coincides with a decrease and plateau in the number of fossil occurrences and genera that mark the major turnover between the White Sea and Nama assemblages. This is supported herein whereby a decline of soft-bodied reconstructed biodiversity at ~551 to 550 Ma is not associated with a decrease in the volume or area of associated shallow to mid-depth siliciclastics (Fig. 4B and fig. S6C). While possible taphonomic issues remain, biotic turnover in this interval is also supported by a shift in assemblage morphogroups across the White Sea–Nama transition. The final disappearance of Ediacaran soft-bodied biota associated with a decline in reconstructed biodiversity across the Ediacaran-Cambrian boundary interval, ~534 to 533 Ma, also does not appear to coincide directly with any notable decrease in volume or area of shallow to mid-depth siliciclastics (Fig. 4B).

Previous analyses have revealed a strong correlation between overall generic diversity and both preserved sedimentary area and the number of sedimentary units through the Ediacaran to Cambrian (*10*). Our full dataset also yields statistically significant positive correlations between mean generic richness and total area of all lithologies and area of shallow to mid-depth siliciclastics (Table 1). This is despite the fact that area estimates per formation/unit are limited for the majority of regions in our dataset due to aforementioned issues associated with variability in the dip of strata, outcrop extent, and subdivision of published geological maps.

The Avalon assemblage represents an exceptional taphonomic window that, as a result, has drawn intense paleontological sampling (Fig. 4E). Increases in mean generic richness in the Avalon assemblage do not coincide with an increase in deep-water siliciclastic rock volume or area, but this assemblage is restricted to the time interval when these rocks are recorded (Fig. 4D and fig. S6F). That the Avalon assemblage is adapted to deeper water is supported by the observation that most Avalon holdovers that persist after 550 Ma are found in the deepest parts of mid-depth settings [e.g., from the Khatyspyt Formation of Siberia and Shibantan Member of the Dengying Formation, South China, although there are some possible exceptions (*24*)]. The first appearance of this assemblage may, however, record the true rise to ecological dominance, as the earlier Ediacaran record contains numerous deep-water siliciclastic successions from ≥620 to 580 Ma that might be expected to host biota in the absence of inhospitable environmental conditions but do not host fossils of Avalon assemblage morphologies.

The White Sea assemblage also represents an exceptional taphonomic window of intense paleontological sampling (Fig. 4E), and so the decline in reconstructed diversity alone may have limited meaning. Nonetheless, notwithstanding persisting uncertainties in the age model, the decline of the White Sea assemblage at 550 Ma appears to represent a true extinction of distinctive White Sea morphogroups, as no decrease in shallow marine siliciclastic volume or area coincides with the disappearance of these taxa.

Notably, the ~555- to 550-Ma interval may preserve few or no carbonate rocks. Therefore, it is possible that calcified cloudinids first appeared >550.5 Ma (or even at 562 Ma, if preservation in dolostones is precluded) and that the apparent global onset of biomineralization inferred by the appearance of calcified biota at ~550.5 Ma may be entirely constrained by availability of carbonate host rocks, with 550 Ma marking the end of a global lowstand and a massive flooding event possibly related, in part, to deglaciation of high- to mid-latitude ice sheets [discussed further in the Supplementary Material, e.g., (*57*–*59*)]. As calcareous biomineralization of cloudinids is inferred to have been under minimal biological control (*69*), it is also possible that this was not facilitated until widespread highly saturated seawaters with respect to carbonate became available. The calcium carbonate saturation state of the ocean may have been elevated at this time and, thus, primed for the emergence of metazoan biomineralization (*70*). Elevated carbonate saturation state would be an expected consequence of the decrease in accommodation space suitable for carbonate deposition due to glacioeustatic lowstand, elevated calcium concentrations that may have resulted from enhanced evaporite weathering during the Shuram interval, and seafloor carbonate dissolution as a direct consequence of oxygenation (*70*). The apparent absence of nonskeletal cloudinids (e.g., *Gaojiashania*) from shallow to mid-depth siliciclastic deposits from ~555 to 550 Ma may also support the established biostratigraphic zonation, whereby the appearance of cloudinids (both skeletal and nonskeletal) postdate the White Sea diversity decline, ~551 to 550 Ma. Moreover, if there was differential preservation in dolostone versus limestone, then we might expect this to also result in no apparent correlation between the volume of dolostone (or total carbonate) and associated mean fossil diversity in the late Ediacaran and lower Cambrian.

We have shown that Ediacaran sampling intensity, >535 Ma, statistically correlates with total mean reconstructed biodiversity (Fig. 5 and Table 1). However, we have not considered occurrence, although previous studies have suggested a very strong positive correlation between the number of occurrences and generic richness during the Ediacaran to Cambrian (*10*). Unfortunately, our biostratigraphic compilation unevenly records the occurrences of organisms across time and geologic units. Therefore, while faithfully representing taxonomic ranges, our data are unsuitable to apply sampling standardization methods such as the shareholder-quorum subsampling approach, which subsamples to equivalent coverage of the underlying taxon-occurrence frequency distribution, relaying on the proportion of recovered specimens from the sample total to estimate coverage (*71*). This underscores that because of the scarcity of fossiliferous Ediacaran successions, sampling dependence on genus richness may continue to present a potentially insurmountable problem in reconstructing accurate estimates of Ediacaran biodiversity.

The Ediacaran record of biodiversity is generally far more patchy (especially before 550 Ma) than most of the Phanerozoic record. The record is also uneven with respect to sampled rock types (Fig. 4E and figs. S4 and S5), as well as intensity of study, collection, and preservational biases, particularly toward siliciclastic surfaces of exceptional preservation. These factors may explain why reconstructed biodiversity does not always correlate with rock volume but does correlate with sampling intensity in the Ediacaran. For example, in Avalonian successions that host the Avalon assemblage, both preservation and collection biases have inflated reconstructed diversity estimates, irrespective of changes in rock volume. The absolute timing of the decline of Ediacaran soft-bodied taxa (temporally constrained only in Laurentia after ~535 Ma) (*18*, *30*) may be related to the fact that the 538- to 533-Ma interval is poorly represented on a global scale due to the sparsity of spatially distributed successions that are currently well constrained in time. This is also an interval of low confidence in the age model that requires future clarification (see Materials and Methods). Preservational style may also, in part, explain the difference in reconstructed biodiversity estimates between the Nama and White Sea assemblages, as in situ soft-bodied fossils on bedding planes within the Nama Group, Namibia, are comparatively unusual compared to White Sea Winter Coast, Russia and Ediacara Member, Australia. By contrast, bedding planes with in situ fossils from the Shibantan Member of the Dengying Formation, South China, which was deposited time equivalent to the Nama Group, show a higher relative reconstructed biodiversity, but given that the Shibantan Member is restricted in its areal extent, this effect may inflate diversity estimates relative to rock area.

The peak in mean genus density at the base of Cambrian Stage 2, ~528 to 527 Ma, which results from normalization of mean generic richness to the volume of host shallow to mid-depth carbonates (Fig. 4C), largely results from fossiliferous transgressive limestones across the Siberian Platform at the base of the “S5” sequence [see (*72*)]. These limestones are locally associated with a basal transgressive lag containing a fossil assemblage of high mean reconstructed biodiversity. Since most of this biota is calcified, phosphatized, or otherwise mineralized (mainly SSFs), this may partially reflect preservational bias. In addition, SSFs are also readily extracted, differentiated, and hence apportioned to different species. These factors are, however, unlikely to be the cause of the recorded gradual increase in mean reconstructed biodiversity through the Terreneuvian. The mean reconstructed biodiversity curves generated here relate to SSFs alone, however, and are therefore far from representative of the Cambrian metazoan biota as a whole, as they do not consider the huge diversity present in the records of Lagerstätten.

### Relationships between global paleoredox, evolutionary dynamics, and eustatic sea level

The observation that minimum δ^238^U_carb_ values become, on average, more positive and oscillations shorter throughout the studied interval (Fig. 6D) might suggest progressive oxygenation and shorter-lived anoxic intervals (Fig. 6E). This, in turn, may reflect a long-term decrease in the capacity of the oceans to buffer against rising environmental oxygen levels due to the protracted depletion of a hypothesized marine organic carbon reservoir that had been maintained under prevailing redox stratified oceanic conditions [e.g., (*43*, *73*, *74*)]. However, the feasibility, and thus existence, of this marine organic carbon reservoir has been the subject of continued debate due to the difficulty in maintaining an organic carbon reservoir of sufficient size to drive observed δ^13^C_carb_ excursions, and modeling results suggest limited change in the size of the oceanic organic carbon reservoir through time [e.g., (*75*)]. Instead, many have advocated for alternative driving mechanisms for Neoproterozoic δ^13^C_carb_ excursions, associated with oxidation of geologic carbon reservoirs [e.g., (*76*)] and/or eustatic sea level changes that may have controlled global changes in the locus and intensity of marine primary productivity and area of shallow carbonate settings conducive to carbonate isotopic reservoir effects (*50*). Our time-calibrated integrated record shows that oscillations in the Ediacaran-Cambrian C─U isotope record that are superimposed upon a long-term trend appear to be related, at least in part, to global sea level changes, which govern the record of sedimentary lithologies and sedimentation rates, weathering, and nutrient delivery, and changes in the areal extent of shelf settings where efficient reductant burial and/or local reservoir effects can take place. Throughout this interval, initiations of major eustatic transgressions postdate δ^13^C_carb_ peaks and often approximate numerous δ^13^C_carb_ minima and δ^238^U_carb_ maxima (Fig. 6, A and D), thus potentially approximating the timing of maximum environmental oxygen availability.

This pattern is consistent with long-term cycles of platform flooding, which increase shelf area and permit efficient organic carbon and pyrite burial during subsequent increasing δ^13^C_carb_. In each case, δ^13^C_carb_ maxima correspond with the onset of OOEs, which most likely began in shallow marine settings in proximity to the atmosphere, and are thought to have been driven by this long-term reductant burial [e.g., (*48*)]. The duration and magnitude of δ^13^C_carb_ minima may reflect changes in the buffering capacity of a putative marine organic carbon reservoir, in part related to waning reservoir size, and/or the influence of changes in the composition and flux of weathering material [e.g., (*43*)]. Alternatively, or additionally, the duration of δ^13^C_carb_ minima may be a more passive reflection of the duration over which globally represented regional effects were in operation, associated with sea level highstands conducive to globally widespread elevated shallow marine area and associated isotopic reservoir effects [e.g., see (*50*)]. In either case, variability in the magnitude of each δ^13^C_carb_ excursion is expected to result, in part, from within- and between-region differences in the degree and style of carbonate diagenesis (*49*). It is highly likely that each of these processes was controlled, to some degree, by sea level in driving changes in accommodation space and depositional regimes, even in the absence of an oversized deep marine organic carbon reservoir.

The BACE represents a rapid, major paired extinction/origination event coincident with the start of the Sauk megasequence and a possible global OOE (Figs. 3B; 6, A to C; and 7B), but the 536- to 534-Ma interval has very low mean generic richness (Fig. 3E), suggesting that these rates may be artificially high. The elevated extinction rate here also occurs during a sustained period of decreased mean reconstructed diversity, and the elevated origination could be due to an edge effect rather than being representative of true macroevolutionary trends. Indeed, some tools may perform poorly except at high and relatively consistent levels of sampling density (*77*). Notwithstanding these concerns, the later early Cambrian short-lived δ^13^C_carb_ excursions have also been tied to paired extinction/origination events (*78*). Such a close pairing of high extinction rates followed by high origination rates is common in the Phanerozoic biotic record (*79*) and suggests the operation of an equilibrium, diversity-dependent dynamic where interspecific competition controls total biodiversity.

In sum, the onset of the Avalon, White Sea, and Cambrian assemblages each appear to coincide with δ^13^C_carb_ maxima, which may also represent the onset of shallow marine OOEs that were triggered by long-term reductant burial permitted, in part, by pulsed increases in eustatic sea level. Eustatic sea level may have also controlled the timing of phosphorite deposition throughout this interval. Phosphorite deposition during Terreneuvian transgressions would have increased the potential for fossil preservation and promoted mechanical concentration, creating an apparent high biodiversity of SSFs in transgressive lags and condensed successions. In addition, the subsequent weathering of phosphorites during sea level lowstands may have further increased nutrient delivery (depending on the style of weathering and the bioavailability of the resulting P input) and fueled productivity, leading to short-term widespread regional deoxygenation and longer-term atmospheric oxygenation [e.g., (*80*)], the latter also ultimately being driven by progressive organic carbon and pyrite burial.

Morphological or ecological novelties such as increased motility and the appearance of segmentation, appendages, or the ability to burrow into sediment must predate their first appearance in the fossil record and subsequent rise to ecological dominance. Moreover, the appearance of skeletal hard parts, with the rise in durophagous predation further promoting protective skeletons, would have substantially enhanced preservable biodiversity in the Cambrian. However, we show here that the temporal and spatial distribution of Ediacaran-Cambrian metazoans adapted to different water depths and sedimentary settings is driven, in part, by the availability of these habitats in the preserved rock record. We further argue that the expansion of habitable settings was likely, in part, controlled by phases of extensive global oxygenation and related nutrient dynamics that were linked to sea level change. This, combined with intrinsic biotic factors such as interspecific competition, would have promoted the potential to capitalize on evolutionary innovations leading to biodiversification. The driver of sea level change therefore served as a fundamental control on Ediacaran-Cambrian metazoan diversification, creating the record of diverse but temporally disjunct metazoan sedimentary habitats and controlling phases of extensive global oxygenation.

By fully integrating geochemical, paleontological, and lithological information into a radiometrically calibrated age framework, we are able to reveal several fundamental features of the late Ediacaran to early Cambrian interval (580 to 510 Ma), each of which may relate to major changes in eustatic sea level. First, paleontological sampling intensity correlates with overall mean reconstructed biodiversity prior to BACE onset. Second, the temporal distribution of the Avalon assemblage appears to be controlled by the availability of preserved rocks that archive their dominantly deep marine siliciclastic habitats, although their first appearance postdates the availability of these rocks and so may record their rise to ecological dominance. The paucity or even absence of shallow carbonate rocks globally from approximately 555 to 550.5 Ma may suggest that the apparent first appearance of metazoan calcareous biomineralization at ~550.5 Ma may be, in part, constrained by the preserved sedimentary record. By contrast, most skeletal Cambrian metazoans are reported from shallow marine carbonate rocks, and there is no clear correlation between mean reconstructed biodiversity of skeletal Cambrian metazoans and the record of these lithologies, unless the lithological record of the Siberian Platform is removed. Some intervals of exceptionally high mean reconstructed biodiversity in the lower Cambrian fossil record are more readily explained by enhanced preservation potential leading to concentrations within carbonate and phosphatic carbonate rocks that often directly relate to sea level change.

The environmental drivers of some apparent radiations during the Ediacaran to Cambrian interval also appear to be related to sea level change, which controls the availability of different sedimentary habitats, and may be linked to carbon cycle perturbations and, by extension, major oxygenation events. This appears to be supported by temporally calibrated δ^238^U_carb_ and δ^13^C_carb_ data, which show a statistically significant antithetic relationship that may support the long-held hypothesis that oxygenation oscillated synchronously with changes to the global marine carbon cycle. The onset timings of major transgressions commonly postdate δ^13^C_carb_ maxima and often approximate δ^13^C_carb_ minima, which may lend further support to models that infer a causal link between sea level, reductant burial, and oxygenation, and/or alternative models that support a sea level driver for observed trends in global isotopic datasets. One mechanism that may help explain the observed pattern is a corresponding increase in the spatial extent of anoxic shallow marine shelf seas and increased nutrient delivery, which would enhance the burial of organic carbon and pyrite and thereby drive increases in atmospheric and oceanic oxygen concentrations.

Major transgressions would therefore both increase habitable shallow marine shelf area and also drive long-term increases in environmental oxygen levels, which culminated in the appearance of the Avalon, White Sea, and Cambrian assemblages. Each of the Ediacaran-Cambrian assemblages marks the appearance of new metazoan morphogroups and, in the case of the Nama and Cambrian, the decline of others. Perhaps most notably, the BACE interval (~536 to 532 Ma) represents a global oxygenation-deoxygenation event at the beginning of the rapidly increasing mean generic diversity of the Cambrian assemblage and approximately coincident with the start of the Sauk megasequence. In sum, the drivers of the appearance of successive early metazoan evolutionary assemblages appear to be related to oxygenation events that were often linked to major sea level transgressions, where sea level further controlled the distribution of diverse habitable settings.

## MATERIALS AND METHODS

### Age framework construction

The global age framework used represents an iteration of an ongoing project aimed at helping to refine Neoproterozoic-Cambrian chronostratigraphy. Age framework construction follows a hierarchical methodology whereby all data (e.g., δ^13^C_carb_ chemostratigraphic and paleontological) from stratigraphic sections that contain radiometrically dated interbeds (e.g., tuff beds with volcanic zircons dated via U─Pb CA-ID-TIMS; table S1 and fig. S1) form the foundational scaffold of temporally calibrated information. All section information is integrated within established regional stratigraphic correlation frameworks to produce regional composite stratigraphic sections. Carbonate δ^13^C_carb_ data from composite sections that lack radiometrically dated interbeds are then correlated with the radiometrically calibrated global δ^13^C_carb_ data scaffold by visual alignment. Last, fossil occurrence information from regional composite sections that are dominantly siliciclastic and do not contain radiometrically dated interbeds or δ^13^C_carb_ data are loosely constrained within the available radiometrically and/or chemostratigraphically calibrated global biostratigraphic scaffold (figs. S1 and S2).

The lithostratigraphic database compiled herein only includes information from those successions for which there is some degree of confidence in their temporal calibration through all, or part, of the ~580- to 510-Ma interval (tables S1 and S2 and data S1 and S2). Hence, there are numerous successions that are omitted because of large remaining uncertainties in precise temporal calibration. Examples of some important fossiliferous successions that are not included in this database are the following: the Krol Group of the lesser Himalaya, northern India, the Asha Group of the south Urals, Russia, the Vestertana Group of northern Norway, several large regions of Laurentia (including sections of the Grand Canyon, British Columbia, the Appalachians, Greenland, and Scotland), and Ediacaran-Cambrian successions of Kazakhstan, Kyrgyzstan, north and west China, Korea, east Sayan of Russia, Antarctica, Spain, Mauritania, and Ghana.

Numerous uncertainties remain in regional stratigraphic and global chemostratigraphic correlations in the late Ediacaran (summarized in fig. S1 and table S2). These are exemplified by correlation uncertainty in the interval of ~538 to 533 Ma and the paucity of carbonate-dominated successions with δ^13^C_carb_ data that are radiometrically constrained in the interval of ~565 to 550 Ma (and especially ~555 to 550 Ma). Correlation uncertainties between approximately 565 and 550 Ma may be partly explained by the far-field effects of a long-lived glacioeustatic sea level lowstand [discussed further in the Supplementary Materials; e.g., (*57*–*59*)]. Given these numerous uncertainties, the global stratigraphic correlation framework presented here, while inherently incomplete, represents the most parsimonious framework in chronostratigraphic terms, when considering all available published data, including radiometric (zircon U─Pb CA-ID-TIMS and Re─Os), chemostratigraphic, and biostratigraphic information.

### Lithostratigraphic database and volume flux estimates

We compiled a summarized lithostratigraphic record for 24 fossil-bearing basin-scale successions across the 580- to 510-Ma interval (data S3, Fig. 2, and fig. S1). These composite successions were prioritized for incorporation into the database as they each include compositional elements that enable temporal calibration (e.g., radiometric or chemostratigraphic data or assemblage-specific fossil constituents). Prioritization of individual sections to create regional composite profiles and prioritization of composite profiles into the global database followed methods outlined in (*28*). Successions that were included contain dated horizons (e.g., via zircon U─Pb or Re─Os geochronology) and/or carbonate carbon isotope data in at least some intervals of ~580 to 510 Ma (fig. S1). Fossil constituents of Cambrian small shelly fossil, trilobite, and archaeocyath biozones were also used to inform the most parsimonious chronostratigraphic fit. By contrast, successions that were not included were those that do not have published radiometric or carbon isotope data (e.g., northern Norway) or where confident regional/global chemostratigraphic correlation is weakened by data scatter or uncertainties in correlation with globally recognized carbon isotope trends (e.g., late Ediacaran sections of the Lesser Himalaya).

For each composite section, summarized lithostratigraphy was subdivided into dominant depositional setting yielding a series of simplified lithofacies (fig. S1 and data S3). These are classified as nonmarine lithological groupings (including fluvial/subaerial/fanglomerate siliciclastics, evaporitic/semirestricted, and volcanic) and marine lithological groupings. Marine lithological groupings include siliciclastic (subdivided into shallow inner shelf, mid-outer shelf to upper slope, and lower slope to basinal), carbonate (first subdivided into limestone and dolostone, and further subdivided into shallow inner shelf, mid-outer shelf to upper slope, and lower slope to basinal), phosphorite, and diamictite (fig. S1, table S2, and data S3).

The maximum area of preserved rock in each region was estimated using either, or a combination of, GoogleEarth polygon area and/or the area of known geological extent using available geological maps and ImageJ software (https://imagej.net/; Fig. 2). Volume fluxes were estimated for each composite succession by multiplying maximum area of preserved rock by average unit thicknesses (e.g., formations/members or intervals of continuous lithofacies type) and dividing total unit volume by the time interval prescribed within the stratigraphic correlation framework, subdivided into 50-ka bins (table S2 and data S1 to S3). Average rather than maximum unit thicknesses were used in rock volume estimates to dampen the effects of using maximum area estimates in each case (e.g., reducing the likelihood of biases imposed by accruing uncertainties on both maximum thickness and area estimates). Where radiometric ages are absent or sparse, the lithostratigraphic database assumes continuous depositional rate, which undoubtedly obscures short-term variability in volume flux.

Estimates of maximum preserved rock area vary in their accuracy due to outcrop extent, variable dip of strata, and availability and resolution of geological maps. Similarly, estimates of average unit thickness vary in their accuracy due to differences in available published information associated with exposure, tectonic disruption, and variability in documentation of lateral changes in unit thickness. For example, while near 100% exposure and minimal tectonic disruption mean that the Nama Group (Namibia and South Africa) is relatively well constrained in terms of rock volume through time; minimal exposure and uncertainties in total terrane area permit only a semiquantitative approximation of preserve rock volume for the Moldova-Podilya Basin. Consequently and with the exception of the Nama Group, we make no attempt to vary prescribed area estimates based on individual rock units throughout a given composite section. As a result, in our database, changes in area through time largely reflect the relative presence/absence of marine sedimentary rock across the compilation of composite successions in aggregate and almost always approximate maximum possible area per region leading to near maximum volume flux estimates in any time slice. In summary, these volume fluxes are estimated, as they accrue uncertainties in global stratigraphic correlation and unit thickness calculations and, (with the exception of the Nama Group) in contrast to the Laurentian Macrostrat database (*67*), do not take into account changes in outcrop area or regional stratigraphic dip, which affect area estimates, through time.

### Diversity metrics

Diversity metrics were estimated for ~580 to 522 Ma using the age-calibrated biostratigraphic occurrence compilation of (*18*). This incorporates datasets and references compiled in (*16*, *17*), updated with more recently published paleontological occurrences, and includes, to our knowledge, all genera described from each of the 24 composite successions compiled in our lithostratigraphic database through the ~580- to 522-Ma interval. This database reports only those fossil occurrences that are, in some way, constrained by the age framework (full details in data S1). The biostratigraphic data are therefore treated as recording the taxonomic ranges through time for each genus rather than a full fossil occurrence dataset.

Trace fossil data were excluded from the diversity and turnover rate estimates. We also excluded fossils identified in the literature solely as holdfasts, as they may represent a variety of different genera already present in the database. Last, we excluded Doushantuo phosphatized embryos (Weng’an assemblage), the Wenghui assemblage of South China, and *Khatyspytia grandis*, due to uncertainties in taxonomic classification (although the latter may be synonymous with *Charniodiscus*).

For Ediacaran taxa, all fossil occurrences were assigned a lithological affinity based on the lithology of the specific beds from which they were reported. This is due to the fact that the vast majority of Ediacaran taxa is reported from siliciclastic units that are often not constrained directly within the age-calibrated δ^13^C_carb_ database but may be constrained within radiometrically calibrated age-depth frameworks (data S1). Furthermore, some Ediacaran occurrences represent taphomorphs reported from both siliciclastic and carbonate strata. By contrast, all Cambrian occurrences in our database are calibrated directly by the δ^13^C_carb_ age framework. Therefore, for Cambrian taxa, all fossil occurrences were assigned a lithological affinity based on the dominant lithofacies of their host formation or section or interval within the host formation.

Considering the highly variable degree of uncertainty associated with the current Ediacaran biostratigraphy, we categorize the possible age ranges for each fossiliferous section, site, or surface according to a level of uncertainty, ranging from 1 to 4, where 1 represents the most certain (bracketing radiometric constraints ± Bayesian age-depth model), 2 indicates carbonate carbon isotope chemostratigraphic best fit (by visual alignment) or combination of radiometric anchors and biostratigraphic best fit, 3 indicates a preliminary biostratigraphic best fit only (e.g., Australian White Sea interval), and 4 represents poorly constrained lithostratigraphic/highly uncertain chemostratigraphic correlations/alignments (uncertainties detailed in table S2). Fossil occurrences categorized as having an uncertainty of 1 are prescribed with the full corresponding temporal range calibrated within the age framework (data S1). By contrast, when the age estimate of a fossil occurrence falls between uncertainties of 2 and 4, a randomly generated occurrence age is prescribed that falls within the age range permitted by uncertainty of the age framework (data S1). These generated ages therefore take into account the uncertainties in calibration of the age model for the different Ediacaran fossil occurrences reported, extending or reducing the range of any given taxon within that uncertainty. Using these criteria, we generated 10,000 different datasets to account for as many combinations of taxonomic temporal range as possible.

We estimated diversity metrics for each of the 10,000 generated datasets using the R package divDyn (*81*), defining 1-Ma moving windows (with 100,000-year movements), as bins to compute the metrics of interest (e.g., a bin identified as “570.5” represents the interval of 570.9 to 570 Ma and is followed by the bin identified as ‘570.4,” which covers the interval of 570.8 to 569.9 Ma). We used genus richness (total number of genera per bin) as a measure of diversity, and, considering that these represent taxonomic range through datasets, we used Foote’s per capita origination and extinction rates (*82*). As stated in the Discussion (“Controls on reconstructed biodiversity metrics”), the lack of a full record of taxonomic occurrences through time units prevents us from applying other more recent approaches, such as Alroy’s gap-filler rates or three-timer rates, as they rely on a measure of “in-bin sampling” that cannot be estimated from our database. Given that Cambrian biostratigraphic data only include fossil occurrences that are temporally calibrated directly within the δ^13^C_carb_ database, they do not account for full lower Cambrian taxonomic ranges. In particular, the Cambrian fossil occurrence database presented herein does not include a number of Lagerstätten (e.g., Maotianshan shales) and cannot be used to estimate last appearances in the Cambrian, as most fossils are known to extend beyond the minimum age of the database. Hence, to avoid artificially high extinction rates and low diversity estimates, we terminate extinction rate estimates at 530 Ma and genus richness estimates at 522 Ma.

Diversity metrics were also estimated for each of the different paleocommunities (Avalon, White Sea, Nama, and Cambrian) and for each lithological affinity category assigned to fossil occurrences (shallow to mid-depth siliciclastic, deep siliciclastic, shallow to mid-depth carbonate, shallow to mid-depth limestone, and shallow to mid-depth dolostone). We calculated and report the average, maximum, and minimum values across all 10,000 datasets for each time bin. Average richness and turnover rates are presented in each corresponding figure, unless stated otherwise. To evaluate the robustness of the fossil record to incomplete sampling across the study interval, we randomly subsampled a third of all reported taxa to build three subsampled datasets, which were then subjected to the same methods as described above.

### Sampling intensity

The number of fossiliferous beds or bedding plane surfaces was used as a measure of sampling intensity through time. For this metric, we evaluated all instances with a body fossil record, including those where holdfasts and *K. grandis* have been recorded, which had been excluded from diversity estimates as previously explained. Whenever the number of surfaces was not available or appropriate, the number of sites/localities or sections was used.

Because our database contains numerous entries that are assigned to a composite section or basin, several heavily sampled areas are underrepresented. To correct for this, we complemented our database by assigning age ranges for some important fossiliferous sections that are included in the full occurrence matrix of (*17*) (e.g., age ranges prescribed to some sections that host the Ediacara Member, Australia, based on preliminary biostratigraphic correlation of constituents of the White Sea paleocommunity). These were identified as a report/collection/locality/section/assemblage to which we could assign an age range within our age model (data S4).

Because of the temporal uncertainty of many Ediacaran fossil occurrences from siliciclastic assemblages or other poorly constrained successions within the model, the age of the fossiliferous surfaces, sites, or sections from those settings was constrained only by the total estimated duration of the host formation/member. In these intervals (e.g., siliciclastic-only successions in the interval of ~560 to 550.5 Ma), the number of surfaces therefore represents the total maximum possible sampling intensity at any given time across the full duration of the corresponding lithostratigraphic unit. We distinguish between those surfaces, sections and sites whose age is relatively well constrained by carbon isotope chemostratigraphic or absolute radiometric dating (color in Fig. 4E) and those that are poorly constrained/uncertain where only biostratigraphic best fit or highly uncertain chemostratigraphic and lithostratigraphic correlations have been possible (gray shown in Fig. 4E).

### Statistical analyses

To compare across our different data compilations and perform correlation statistics, we used moving mean windows that were equivalent to the intervals used to construct our reconstructed biodiversity data. Our lithostratigraphic compilation was grouped and averaged across 1-Ma bins, which moved in 100-ka increments. We used Spearman’s rank (ρ) to test for monotonic associations (Table 1). Since moving mean averages are autoregressive data models, we also applied this statistical test over the first differences of both our diversity and lithostratigraphic data.

Similarly, our sampling data were also binned in 1-Ma bins, and the moving sums of the number of sites, sections, or surfaces were calculated every 100 ka. We also used Spearman’s ρ to evaluate associations to the reconstructed biodiversity data. Given the unevenness of the resolution and duration of our sampling metrics and the correlation uncertainties of the age framework in key intervals of high diversity (e.g., the White Sea interval in Australian and East European Platform successions), we stretched the duration of several sections across their maximum possible range. As a result, a large proportion of first difference values were artificially flattened across the zero line, which led us to conclude that the transformation was unsuitable to further analyze the data.

Last, statistical correlations between uranium and carbon isotopic data were also evaluated using Spearman’s ρ. The 10-point moving average (and 10-point moving minimum for δ^238^U_carb_) was preferred because of the large sampling gaps in the uranium record across the middle Ediacaran. We also compared the associations of first differences. All statistical tests were performed using the stats package on R statistical software, and the results are presented in Table 1.

## Supplementary Materials

### Other Supplementary Material for this manuscript includes the following:

Data S1 to S4

Auxiliary References
