# Supplementary material for: Sea level controls on Ediacaran-Cambrian animal radiations
Source: Sci Adv. 2024 Jul 31;10(31):eado6462. doi: 10.1126/sciadv.ado6462 (PMC11290527; doi:10.1126/sciadv.ado6462)
Supplement: Supplementary file 1 — Supplementary Text Figs. S1 to S6 Tables S1 and S2 Legends for data S1 to S4 [file sciadv.ado6462_sm.pdf]

Science Advances
AAAS

Supplementary Materials for

Sea level controls on Ediacaran-Cambrian animal radiations

Fred T. Bowyer *et al.*

Corresponding author: Fred T. Bowyer, fred.bowyer@ed.ac.uk

Sci. Adv. **10**, eado6462 (2024)
DOI: 10.1126/sciadv.ado6462

The PDF file includes:

Supplementary Text
Figs. S1 to S6
Tables S1 and S2
Legends for data S1 to S4

Other Supplementary Material for this manuscript includes the following:

Data S1 to S4
Auxiliary References

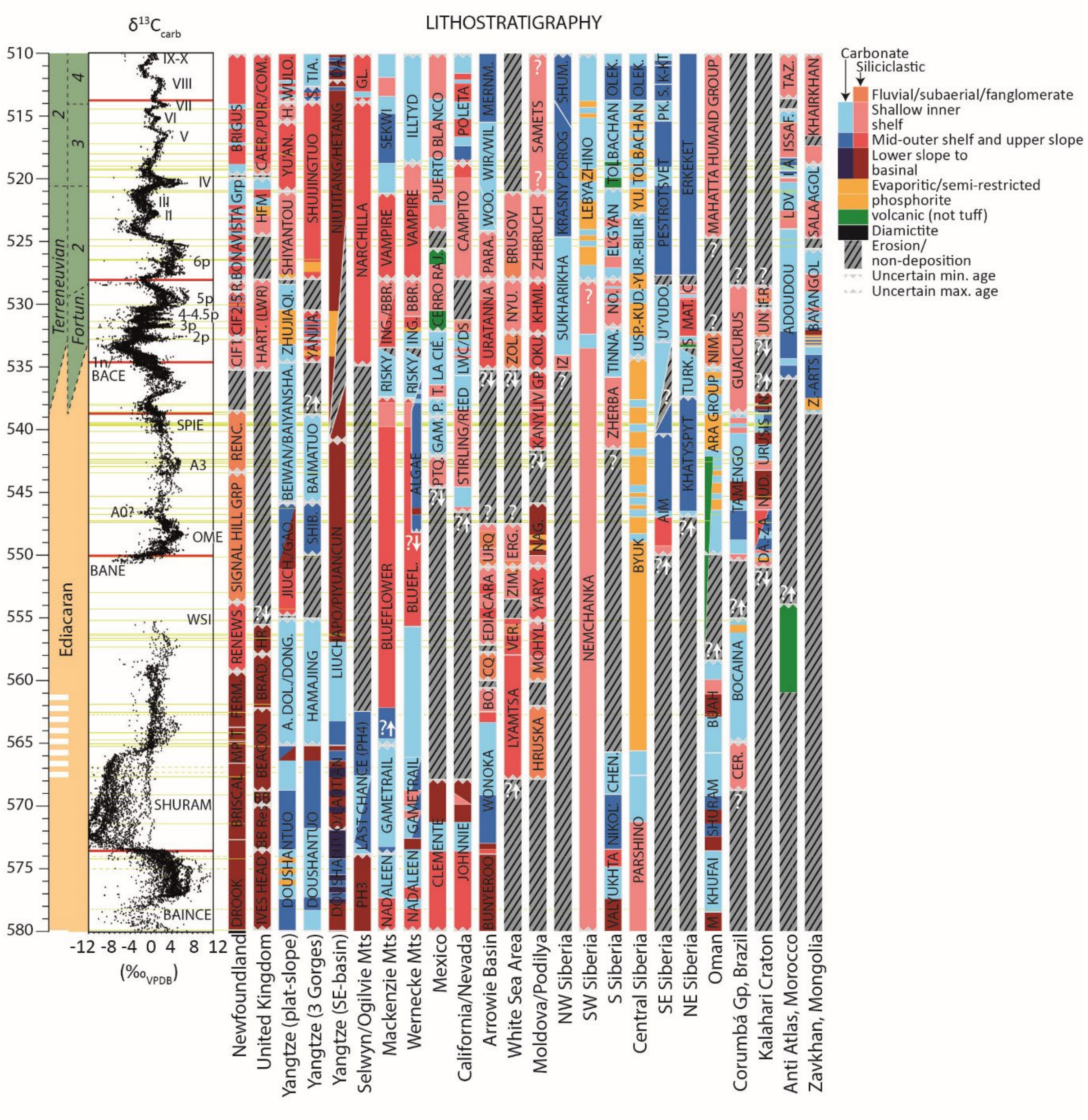

Fig. S1: Carbon isotopic and lithostratigraphic correlation employed in this age model [modified Model K, after ref. (*18*)]. See Tables S1, S2, and Data S1, S2 for full details.

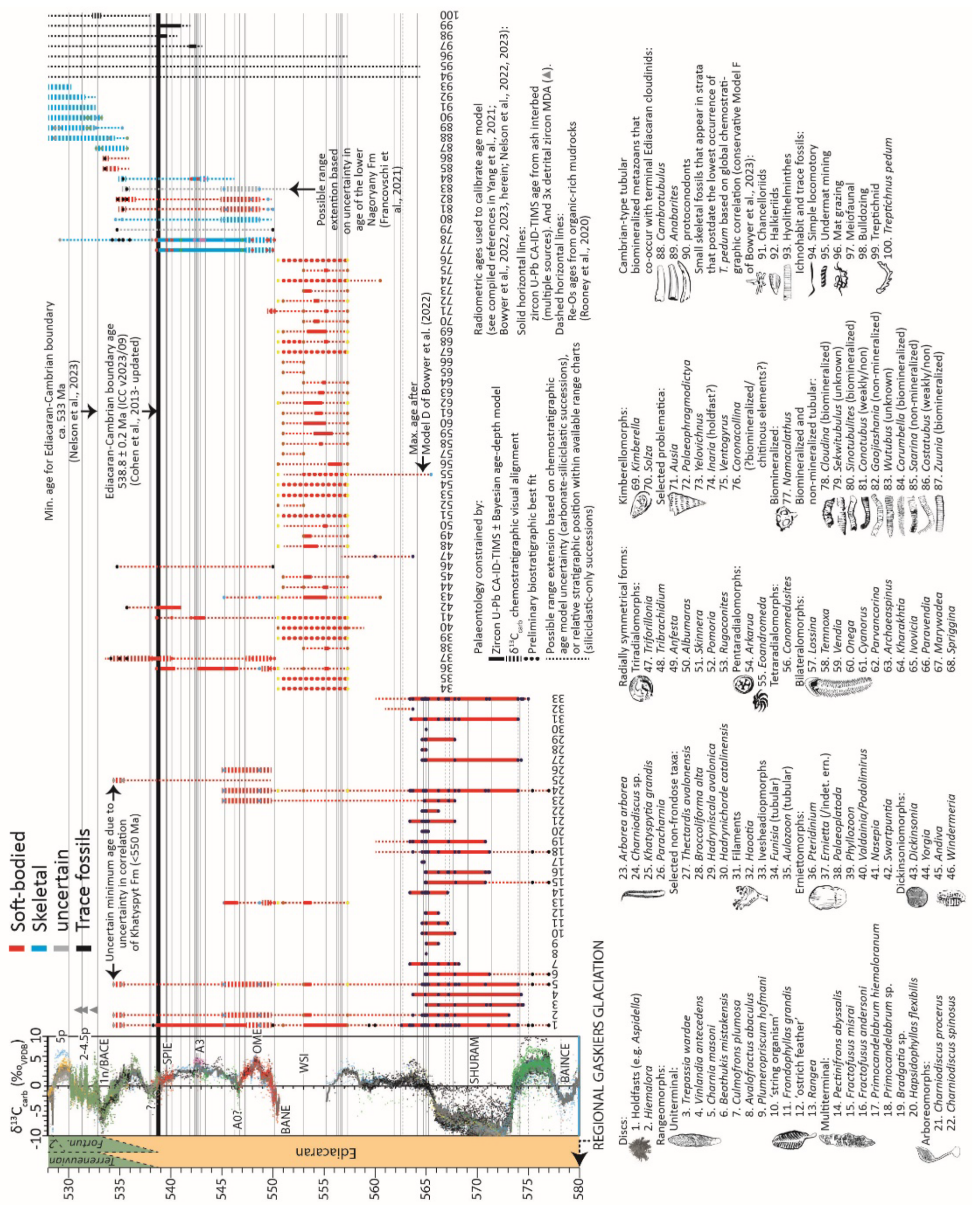

Fig. S2: Chemostratigraphic and biostratigraphic correlation employed in this age model [modified after ref. (*18*)]. Carbon isotope data are colored according to provenance, with smoothed grey line showing 10-point moving average of all data. See Data S1 and Methods for full details.

The age and paleolatitudinal distribution of ca. 565–550 Ma glacial deposits

There is growing evidence in support of an interval of post-Gaskiers (and post-Shuram excursion) Ediacaran glaciation (*57–59*, *83–86*). Precise depositional ages for the majority of post-Gaskiers Ediacaran glacial deposits, and the associated duration and paleolatitudinal extent of glaciation(s), remain uncertain. However, detrital zircon studies of Cadomian glacial deposits (including Armorican, Bohemian and Iberian massifs) yield a consistent maximum depositional age (MDA) of ca. 565 Ma (*58*). Although there remain numerous uncertainties in paleogeographic reconstructions, partly associated with the complexities of Ediacaran paleomagnetic data [see recent review in (*87*)], some reconstructions [e.g., (*88*)]

place Cadomia on the periphery of Gondwana at ca. 565 Ma, in close proximity to the Moroccan Anti-Atlas (Fig. S3). The Bou-Azzer Inlier of the eastern Anti-Atlas hosts diamictite deposits that are interpreted as glacial (*84*), and zircon U-Pb LA-ICP-MS dating of associated ignimbrites yields an age of ca. 567 ± 4 Ma (*59*). Together, these data appear to support a high latitude glaciation ca. ≤565 Ma (Fig. S3D, E). Similarly, the youngest zircon population of an ash bed in the middle Dhaiqa Formation of Saudi Arabia (reconstructed between 30-60°S), which underlies diamictite deposits interpreted as glacial, yields a U-Pb LA-ICP-MS MDA of 560 ± 4 Ma (*58*, *86*).

In the age model herein, low latitude carbonate deposition continues until at least ca. 555 Ma, based on the zircon U-Pb CA-ID-TIMS age of a tuff deposit interbedded with dolostone of the Bocaina Formation, Brazil [Table S1, (*89*)]. However, this age model entertains the possibility that the lowest latitudinal extent of glaciation occurred between ca. 555 Ma and ca. 550 Ma, and that this interval coincided with a global paucity of carbonate deposition (Fig. S3F). This is based on both the absence of radiometrically constrained carbonate deposits in this interval and the potentially low paleolatitude (*88*) of Iran, where detrital zircon U-Pb data constrain an MDA of ca. 560–550 Ma for inferred-glacial diamictite deposits in the Kahar Formation of the Central Elborz Mountains (*90*). This possible glacioeustatic sea level lowstand may also be responsible for a globally widespread unconformity that caps underlying carbonate units [e.g., the unconformable boundary between the Bocaina and overlying Tamengo formations, Brazil (*89*, *91–93*), and the boundary between the lower and middle members of the Dengying Formation, South China, following the possible Model D lithostratigraphic correlation described in ref. (*94*)].

The subsequent transgressive surface at 550.5 Ma is most pronounced in, but synchronous between, those regions with active tectonics that were conducive to the creation of accommodation space, including the Kalahari foreland basin and the extensional basins of South China. Elsewhere, this transgression is far less pronounced as the facies are shallower. Indeed, shallowing of sedimentary rocks is noted from ca. 550–533 Ma in some regions, including Avalonia, Australia, and Moldova (Fig. S1). This may be due to lower gradient platform geometries and/or the final infill of available accommodation space.

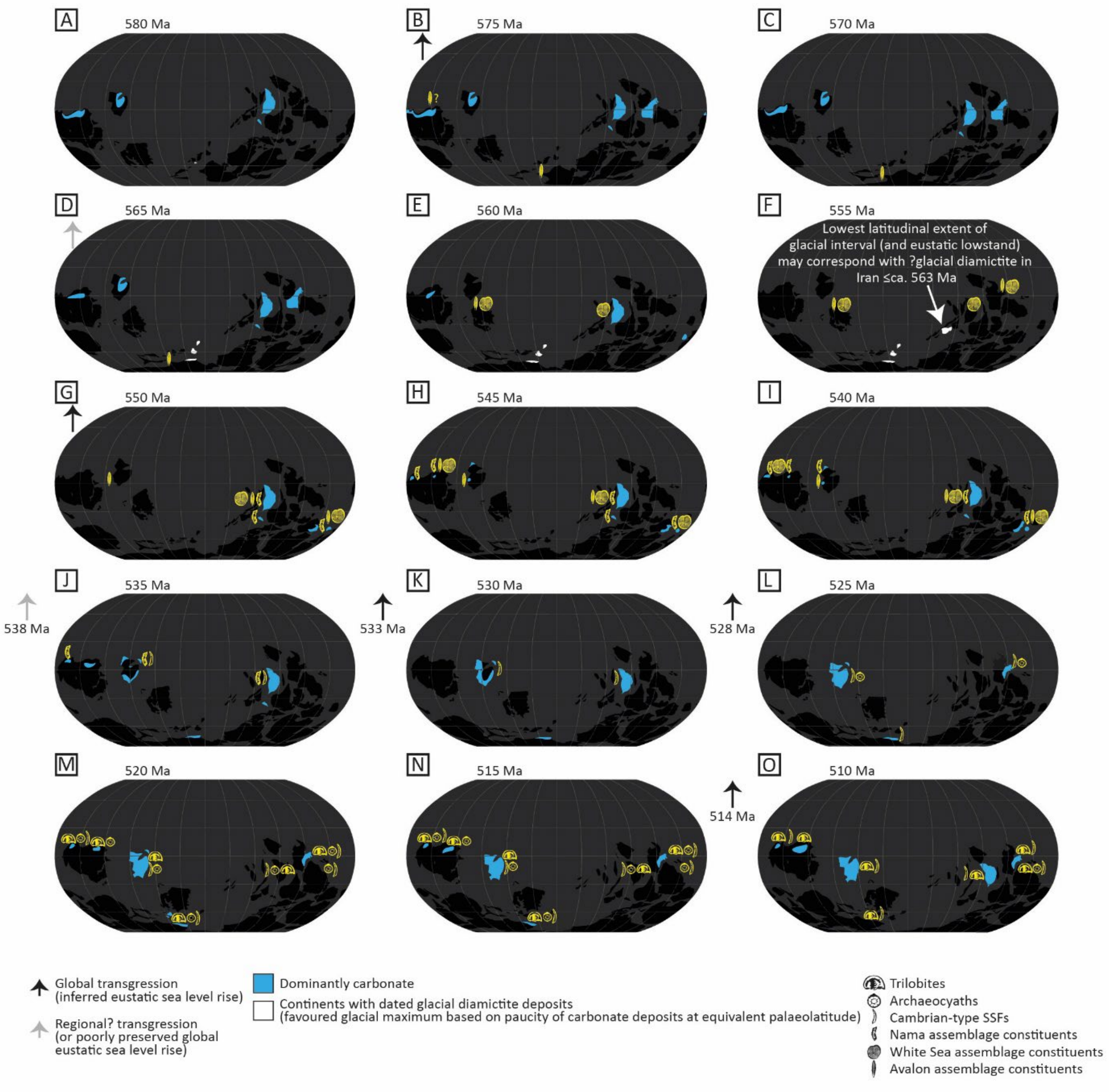

Fig. S3: Paleolatitudinal distributions of key biota, carbonate deposits and inferred glacial deposits at 5 Myr time slices according to the age model herein. Paleogeography after ref. (*88*) but note ongoing uncertainty in precise paleolatitude of terranes peripheral to Gondwana (highlighted by problematic high latitude positions for Cambrian carbonate-hosting Cadomian, Moroccan and Avalonian terranes) that may be reconciled by Gondwana apparent polar wander (*95*), or numerous other mechanisms [e.g., see review in (*87*)]. The implications of alternative paleogeographies are numerous throughout this interval, as recently demonstrated by ref. (*96*).

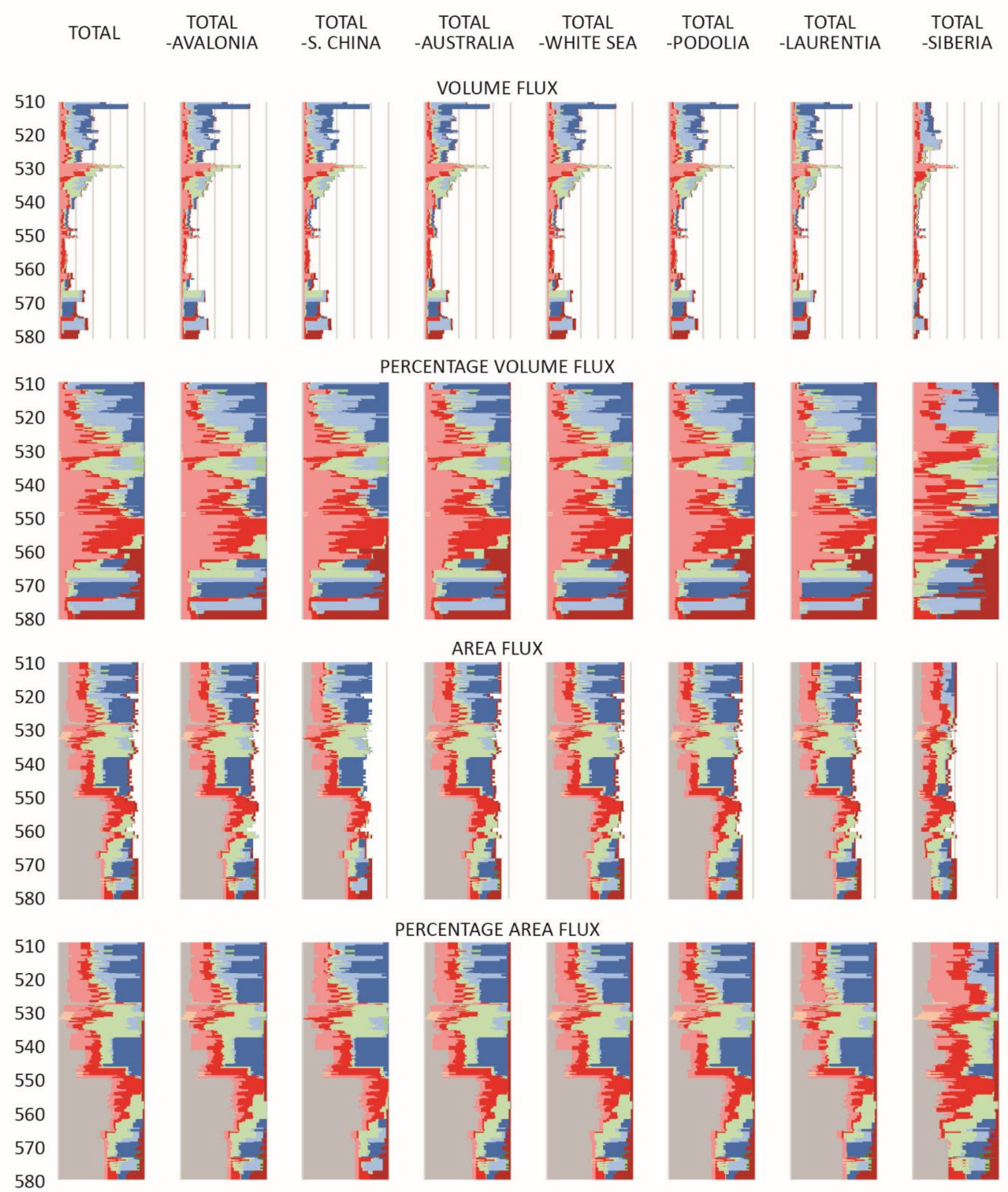

Fig. S4: Sensitivity tests showing the effects of removing key regions, and the resulting changes in total marine sedimentary rock volume flux (and percentage) and area flux (and percentage). Note differences in patterns and completeness of area flux that result from the removal of the Siberian Craton record alone. Full details and sensitivity test calculations are presented in Data S3. See Fig. 3 for key to color.

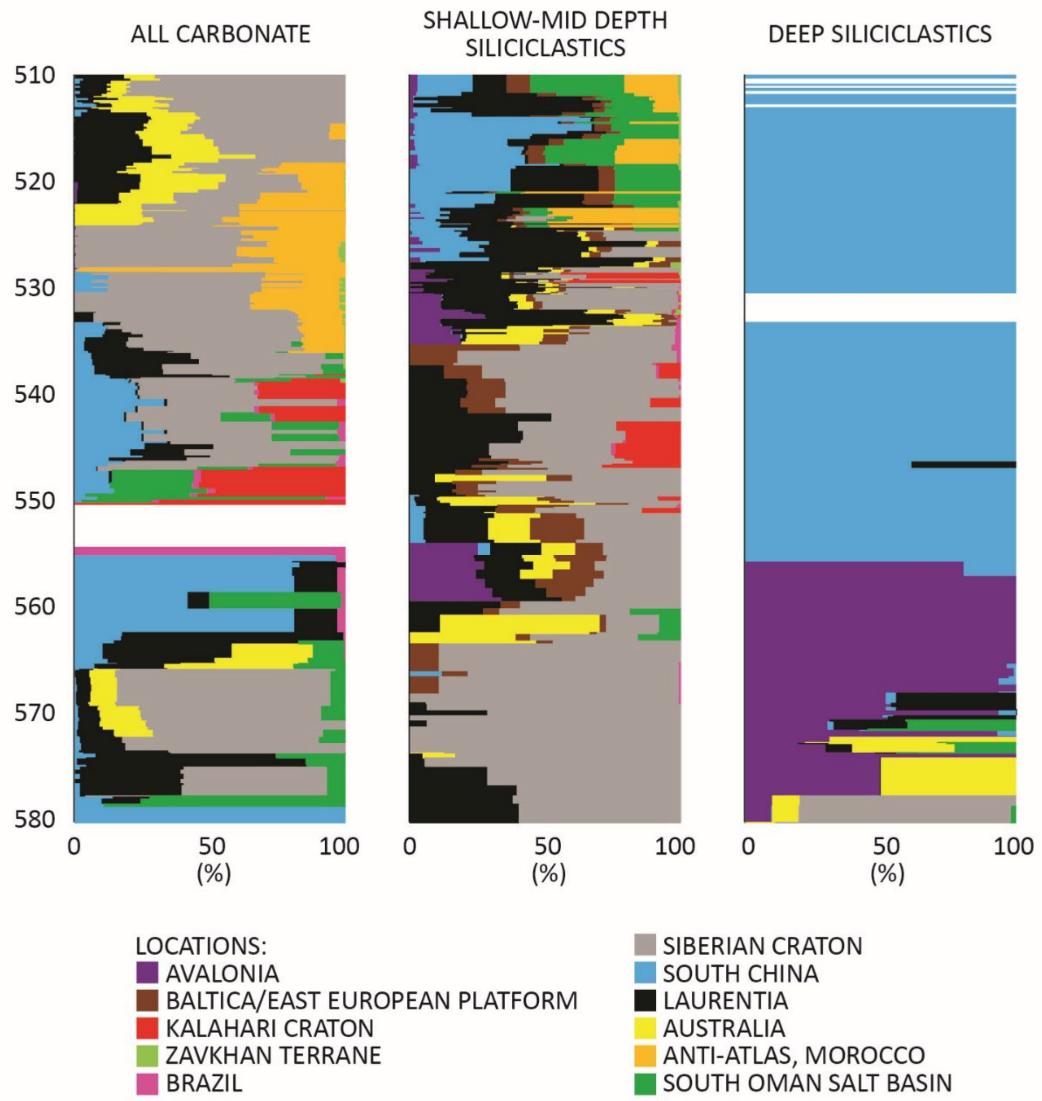

Fig. S5: The regional representation of percentage volume flux, subdivided into all carbonates, shallow-mid depth siliciclastics and deep siliciclastics. Full details are presented in Data S3.

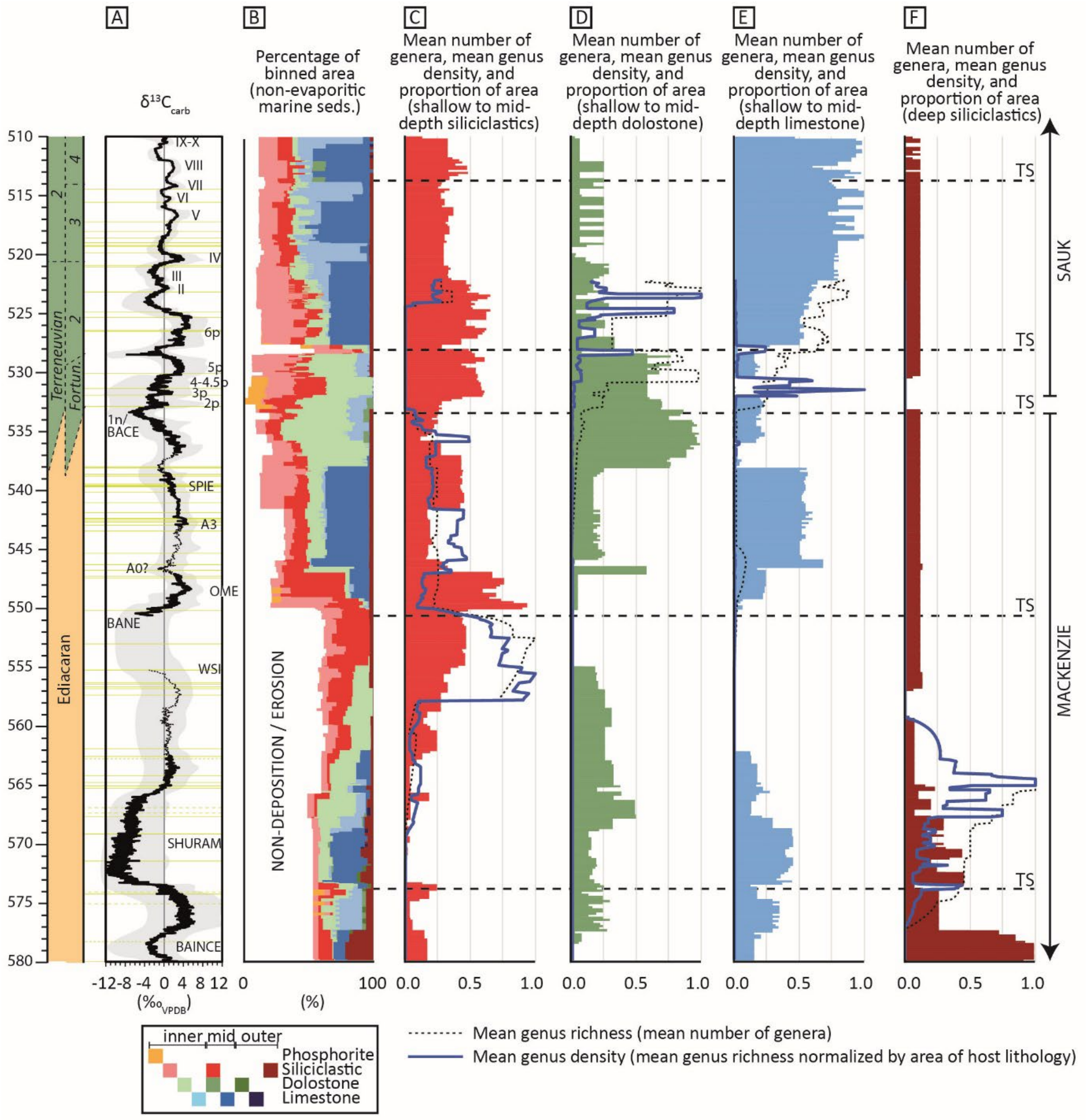

Fig. S6: Comparison of percentage binned area of marine sedimentary rocks and lithology-specific metazoan distribution. (A) Updated global composite $\delta^{13}C_{carb}$ curve with uncertainty [Data S1, modified after (*18*, *28*)] calibrated to radiometric dates (Table S1). **(B)** Percentage of binned area of marine sedimentary rocks. **(C)** Normalized area of shallow to mid-depth siliciclastic rocks and normalized distribution of associated metazoans. **(D)** Normalized area of shallow to mid-depth dolostone and normalized distribution of associated metazoans. **(E)** Normalized area of shallow to mid-depth limestone and normalized distribution of associated metazoans. **(F)** Normalized area of deep siliciclastic rocks and normalized distribution of associated metazoans. In **(C)-(F)**, proportional area of sedimentary rocks and genus density are normalized to 1.

Table S1. Updated after ref. (***94***)

Radiometric ages (several Cambrian ages are listed in GTS2020)

Italicized ages are not included in the compilation for the reasons provided.		
**Age (Ma)**	**Details**	**Reference**
**486.78 ± 0.53 (2.57)**	Zircon U-Pb air abrasion ID-TIMS age of a crystal-rich volcanic sandstone bed at the sequence boundary between the Dolgellau Fm and Dol-cyn-afon Fm, Mawddach Group at Bryn-llin-fawr, Harlech Dome, N. Wales. Fourteen single zircon grains yield weighted mean $^{207}Pb/^{206}Pb$ age of 486.78 ± 2.57 Ma. Recalculated by ref. (*97*) using the updated U decay constant of ref. (*98*). "Dated horizon is close to the top of the *Acercare* Zone. Dated ash is 4m below the regional lowest occurrence of *Rhabdinopora*, and 5m below *R.f. parabola*. Very close to the Cambrian-Ordovician boundary." After ref. (*97*)	(*99*)
**488.71 ± 1.17 (2.28)**	Zircon U-Pb air abrasion ID-TIMS age of a crystal-rich volcanic sandstone bed in the lower Dolgellau Fm, Mawddach Group at Ogof-ddu, Criccieth, N. Wales. Nine multigrain zircon fractions yield weighted mean $^{207}Pb/^{206}Pb$ age of 488.71 ± 1.17 (2.78) Ma. Recalculated by ref. (*97*) using the updated U decay constant of ref. (*98*). Dated horizon calibrates the lower *Peltura scarabaeoides* Zone. "*Peltura scarabaeoides scarabaeoides* below and *P.s. westergardi* above indicate the third subzone (*Parabolina lobata* Subzone) of the *Peltura scarabaeoides* Zone in Norway." After ref. (*97*)	(*100*)
**≤492.83 ± 0.71**	Zircon U-Pb CA-ID-TIMS maximum depositional age for an othroquartzite at the top of the Worm Creek Member of the St Charles	(*101*)

	Formation, Smithfield Canyon, Utah (sample HC-20-77). (n=3)	
**≤494.16 ± 0.46**	Zircon U-Pb CA-ID-TIMS maximum depositional age for an othroquartzite at the base of the Worm Creek Member of the St Charles Formation, Smithfield Canyon, Utah (sample TF21-SC1). (n=10)	(*101*)
**≤494.35 ± 0.46**	Zircon U-Pb CA-ID-TIMS maximum depositional age for a 0.25m-thick calcareous sandstone bed in the upper Nounan Formation, Smithfield Canyon, Utah (sample TF21-SC2). (n=6)	(*101*)
**494.6 ± 2.9**	Re-Os age from the Alum shale, Andrarum-3 core. Approximates peak organic carbon isotope data that correlate to the acme of the SPICE excursion. Initial $^{187}Os/^{188}Os = 0.82 \pm 0.01$. (2σ age, MSWD = 1.3, n = 7)	(*102*)
**502.10 ± 2.40 (3.50)**	Zircon Pb-Pb age from a tuff breccia in the Taylor Fm of the Taylor Nunatak, Shackleton Glacier, Antarctica. Two multigrain zircon fractions (sample TAY-F), processed via air abrasion ID-TIMS. Sample TAY-F is 15m below 3m-thick carbonate horizon that yielded trilobites *Amphoton* sp. cf. *Amphoton oatesi* and *Nelsonia* sp. cf. *Nelsonia schesis*. Ref. (*103*) suggest an upper Floran to Undillian Stage based on biostratigraphic correlation with Australia. This equates to the middle Drumian Stage, and is approximately equivalent to the middle Mayan Stage of Siberia.	(*103*)
**503.14 ± 0.13 (0.59)**	Zircon U-Pb CA-ID-TIMS age from a subaerial pyroclastic tuff 1.0 m above the base of the Triebenreuth Fm, Franconian Forest, Germany (sample Trieb-1). Five (of seven) single grain analyses, MSWD = 0.74. Drumian based on trilobite biozonation. Poorly preserved but diverse assemblage including	(*104*)

	trilobite sclerites, echinoderm ossicles, brachiopod and mollusk valves, hyolith conchs, and sponge spicules, from calcareous nodules and calcareous, silicieous shales ~40m above Tribenreuth volcaniclastic rocks. Trilobites include eodiscinids, corynexochids and ptychopariids.	
**506.25 ± 0.07 (0.16) [0.55]**	Zircon U-Pb CA-ID-TIMS age (weighted mean $^{206}Pb/^{238}U$) of tuff bed in the lower Braintree Member of the Chamberlain's Brook Formation, Red Bridge Road, east side of Conception Bay, southeastern Newfoundland (sample RBCB-1.2). (MSWD = 0.78, n = 8). Immediately above unconformity that caps *Callavia broeggeri* Zone, and within an interval without established biostratigraphic zonation.	(*105*)
**506.34 ± 0.21 (0.25) [0.60]**	Zircon U-Pb CA-ID-TIMS age (weighted mean $^{206}Pb/^{238}U$) of tuff bed in the Braintree Member of the Chamberlain's Brook Formation, Smith Point/Sound section, Trinity Bay, southeastern Newfoundland (sample SPCB-4.4). (MSWD = 0.08, n = 5). Immediately above an unconformity at the base of the *A harlani* Zone.	(*105*)
**507.21 ± 0.13 (0.19) [0.58]**	Zircon U-Pb CA-ID-TIMS age (weighted mean $^{206}Pb/^{238}U$) of tuff bed in the Braintree Member of the Chamberlain's Brook Formation, Redland Point/Cove section, St Mary's Bay, southeastern Newfoundland (sample ReBr-118.5). (MSWD = 0.04, n = 4). Within the *Morocconus*-*Condylopyge eli* Assemblage Zone.	(*105*)
**507.67 ± 0.08 (0.17) [0.55]**	Zircon U-Pb CA-ID-TIMS age (weighted mean $^{206}Pb/^{238}U$) of tuff bed in the upper Jigging Cove Member of the Brigus Formation, Smith Point/Sound section,	(*105*)

	Trinity Bay, southeastern Newfoundland (sample SPBr-105.75). (MSWD = 1.26, n = 6). Within an interval without established biostratigraphic zonation. Immediately below an unconformity at the base of the *A. harlani* Zone, and above the *Myopsostrenua* cf. *M. sabulosa* Zone (and above the '*Geyerorodes*? *Howleyi*' Assemblage Zone).	
**507.91 ± 0.07 (0.16) [0.55]**	Zircon U-Pb CA-ID-TIMS age (weighted mean $^{206}Pb/^{238}U$) of tuff bed in the upper Saint Mary's Member of the Brigus Formation, Red Bridge Road, east side of Conception Bay, southeastern Newfoundland (sample RBBR-26.5). (MSWD = 0.28, n = 6). Within *Callavia broeggeri* Zone.	(*105*)
**509.10 ± 0.33 (0.62)**	Zircon U-Pb CA-ID-TIMS age for a bentonite bed within the basal Quarry Ridge Gritz, basal Upper Comley Sandstone Fm (sample Comley ub). Seven single grain analyses, MSWD = 0.51. *Paradoxides harlani* and other trilobites in immediately overlying beds. *P. harlani* trilobite zone of upper Stage 4, Series 2. Geyer (2019) notes this as the *Protolenus* Zone	(*106*)
**508.05 ± 1.13 (2.75)**	Zircon U-Pb air abrasion ID-TIMS age for a volcanic ash bed 9.5m above the base of the Hanford Brook Fm (middle Somerset Mb), Saint John, New Brunswick (sample SoS-56.1). Eight single grains or small multigrain fractions. Age recalculated using the U decay constant of ref. (*98*) by ref. (*97*). *Protolenus howleyi* trilobite Zone, Late Branchian, Stage 4, Series 2. Used to suggest an age for the base of Series 3/Stage 5 of 507 Ma (*97*).	(*107*)
**511.87 ± 0.14 (0.28) [0.60]**	Zircon U-Pb CA-ID-TIMS age for a tuff in the Billy Creek Formation (upper part of the Warragee Member),	(*108*)

	Angorichina, Flinders Ranges (sample R1300537). Eight single grain analyses (MSWD = 0.9, probability of fit = 0.48). Age from middle-upper *Pararaia janeae* Zone.	
**514.45 ± 0.43 (0.69)**	Zircon U-Pb CA-ID-TIMS age for a bentonite bed several cms below the top of the Green Callavia Sandstone, uppermost Lower Comley Sandstone Fm (sample Comley 1b). Two (of seven) single grain analyses, MSWD = 2.0. *Callavia* trilobite zone of upper Stage 3 of Series 2.	(*106*)
**514.46 ± 0.13 (0.28) [0.60]**	Zircon U-Pb CA-ID-TIMS age for a tuff from a volcaniclastic layer in the Mernmerna Formation in the Paralana 1B DW1 drill core (sample 1830309). Six single grain analyses (MSWD = 1.6, probability of fit = 0.17). Age from middle-upper *Pararaia bunyerooensis* Zone.	(*108*)
**514.56 ± 0.13 (0.28) [0.60]**	Zircon U-Pb CA-ID-TIMS age for a 10cm thick green tuff bed in the Third Plain Creek Member of the Mernmerna Formation, Bunkers Range (sample R1300538). Eight single grain analyses (MSWD = 1.2, probability of fit = 0.29). Age from middle-upper *Pararaia bunyerooensis* Zone.	(*108*)
**515.38 ± 0.13 (0.28) [0.60]**	Zircon U-Pb CA-ID-TIMS age for a tuff bed 25m above the base of the Mernmerna Formation, Bunyeroo Gorge, Flinders Ranges (sample BG Tuff). Eight single grain analyses (MSWD = 2.0, probability of fit = 0.06). Age from middle-upper *Pararaia bunyerooensis* Zone.	(*108*)
**515.56 ± 1.03 (1.16)**	Zircon U-Pb age from the upper Lemdad Fm (equivalent to Lower Issafen Fm) of the Lemdad syncline, Morocco (section Le-XI). Five single grain analyses, originally processed via air abrasion in (*107*), and recalculated by (*109*) using	(*107*, *109*)

	updated U decay constant (see (*109*) for details). Lower Botoman based on trilobite biostratigraphy. Marks onset of peak V? or VI? in Morocco.	
**517.22 ± 0.31 (0.40) [0.66]**	Zircon U-Pb CA-ID-TIMS age of bentonite 7m above the base of the Purley Shale Fm, Woodlands Quarry, Warwickshire, England (Avalon terrane). Five of nine single grain analyses (samples z1-4, z12), MSWD = 0.67.	(*110*)
**518.03 ± 0.69 (0.71)**	Youngest zircon U-Pb CA-ID-TIMS age (incorporating U-Pb tracer calibration uncertainty) of five single grain analyses of detrital zircons from the Maotianshan shale immediately underlying Chengjiang biota. Taken as maximum depositional age for the Chengjiang biota.	(*60*)
**518.59 ± 0.20 (0.32) [0.63]**	Zircon U-Pb CA-ID-TIMS age of tuff bed in the upper Amouslek Formation, Timoulaye Izder section, Morocco (sample Tim-269.5). (MSWD = 0.22, n = 3). Within the upper *Choubertella* or possibly lower *Daguinaspis* Zone.	(*111*)
**518.99 ± 0.14 (0.20) [0.58]**	Zircon U-Pb CA-ID-TIMS age of tuff bed in lower Amouslek Formation correlated to neighbouring section at Tazemmourt to upper *Choubertella* Zone. Sample Ti-Am-34.0 (MSWD = 0.38, n = 6).	(*111*)
**519.30 ± 0.23 (0.57) [0.77]**	Zircon U-Pb CA-ID-TIMS age of the Caerfai Bay Shales Fm at Cwm Bach, Pembrokeshire, South Wales (Avalon terrane). Six of seven fractions (single grains or fragments) from sample Cwm Bach 1 (MSWD = 1.1, n = 6).	(*106*)
**519.23 ± 0.14 (0.21) [0.58]**	Zircon U-Pb CA-ID-TIMS age of tuff bed that rests unconformably atop the Tiout Member, in the basal Amouslek Formation (sample Ti-Am-0.0). Transition between upper *Fallotaspis plana* and lower	(*111*)

	Choubertella zones. Approximate zero-crossing point of recovery from peak IV.	
**519.87 ± 0.24 (0.35) [0.64]**	Zircon U-Pb CA-ID-TIMS age of brown-weathering dolomitic feldspathic sandstone 8.5m below trilobite horizon T1 (sample Ti-I-neg8.5). Within lower member of the Igoudine Formation. Interpreted as a maximum depositional age. Large relative uncertainty (MSWD = 2.60, n = 2).	(*111*)
**520.93 ± 0.14 (0.28) [0.61]**	Zircon U-Pb CA-ID-TIMS age of tuff bed in the upper Lie de vin Formation, Tiout section, Anti Atlas Mountains, Morocco (sample M236). Six single grain analyses (MSWD = 0.42, n = 6). Ash bed at base of rising limb of peak IV.	(*112*)
**521.06 ± 0.12 (0.28) [0.61]**	Zircon U-Pb CA-ID-TIMS age of tuff deposit 210m below top of Lie de vin Formation in the Tiout section, 500m below base of the Tiout Member, 310m below peak IV (sample Tiout-566). (MSWD = 0.61, n = 7)	(*111*)
**523.17 ± 0.16 (0.42) [1.0]**	Zircon U-Pb CA-ID-TIMS age of tuff bed in the lower Lie de vin Formation at Oued Sdas section, Anti Atlas Mountains, Morocco (sample M234). Ten single grain analyses (MSWD = 1.2, n = 10) Ash bed at level immediately prior to peak II.	(*112*)
**524.837 ± 0.092 (0.35) [0.93]**	Zircon U-Pb ID-TIMS age from tuff bed in the upper Tifnout Member (Adoudou Formation) at Oued Sdas section, Anti Atlas Mountains, Morocco (sample M231). Mixture of 5 air abraded and 3 chemically abraded single grain analyses (MSWD = 0.72, n = 8) Ash bed within falling limb of peak 6p.	(*112*)
**525.343 ± 0.088 (0.35) [0.93]**	Zircon U-Pb ID-TIMS age from a tuff bed in the middle Tifnout Member (Adoudou Formation) at Oued Sdas section, Anti Atlas Mountains, Morocco (sample	(*113*), updated in (*112*)

	M223). Mixture of 6 air abraded and 6 chemically abraded single grain analyses (MSWD = 0.33, n = 11). Ash bed at/immediately after max values of peak 6p.	
**526.43 ± 0.54 (0.64) [0.85]**	Zircon U-Pb CA-ID-TIMS age from a tuff bed within the lower Shuijingtuo Formation, Yanjiahe section, Yangtze Gorges area (sample 16YC03). Age represented weighted mean ^{238}U-^{206}Pb age from five (of six) single grain analyses (MSWD = 0.30, n = 5). Minimum age constraint on the ZHUCE	(*66*)
**526.5 ± 1.1**	SHRIMP U-Pb age of the base of the Badaowan (Shiyantou-equivalent) Formation, bed 9 at Meishucun section, Yunnan Province, South China. Note that the ZHUCE excursion in models B and C is curtailed early at Meishucun, consistent with this age. However, the ZHUCE excursion at Xiaotan section is considered to correlate with the entirety of 6p in these models.	(*114*)
**530.02 ± 1.2**	Zircon ID-TIMS (air abrasion) age for tuff bed 24.32 – 24.58m above the base of the Chapel Island Formation, Ratcliffe Brook Group, Somerset Street, Saint John, southern New Brunswick (sample SoS-24.4). Three multigrain fractions. Approximate age of the Chapel Island Formation lithofacies association (Member) 5 (Mystery Lake Member) after regional litho- and biostratigraphic correlation with sections in Saint John, New Brunswick (*115*, *116*). Middle part of trace fossil zone *Rusophycus avalonensis*, Placentian Series.	(*117*), recalculated in (*97*)
**529.7 ± 0.3**	Zircon U-Pb CA-ID-TIMS age for tuff deposit in upper Mattaia Formation, Mattaia Creek mouth (above FAD *Aldanella attleborensis*). *No detailed information*	(*118*, *119*)

	available on the number of single grain analyses, concordance or MSWD for this age, as it was published in abstract form only. However, if correct, this places a minimum age constraint on the FAD of *A. attleborensis* which is consistent with a first appearance approximately contemporaneous with peak 5p in Siberia, as previously suggested.	
**531.32 ± 0.66 (0.68) [0.88]**	Zircon U-Pb CA-ID-TIMS age from a micaceous pale green siliceous siltstone ~9m above L1906 and ~6m below the base of the middle member of the Wood Canyon Formation, Spring Mountains, Nevada (sample L2008-10.1A). Interpreted as a maximum depositional age. Four (of six) single grain analyses (MSWD = 1.0; probability of fit [p(χ^2)] = 0.39).	(*30*)
**531.86 ± 0.69 (0.70) [0.90]**	Zircon U-Pb CA-ID-TIMS age from a <0.2 m thick lenticular and discontinuous bed of siliceous green mudstone to siltstone in between beds of coarse sandstone to pebble conglomerate within the upper part of the lower Wood Canyon Formation. The sample comes from an interval dominated by peritidal to shallow marine quartz sandstone that directly underlies fluvial facies of the middle member, and overlies the lowest occurrence of *T. pedum*. Spring Mountains, Nevada (sample L1906). Interpreted as a maximum depositional age. Six (of eight) single grain analyses (MSWD = 1.6; probability of fit [p(χ^2)] = 0.16).	(*30*)
**532.83 ± 0.98 (1.00) [1.14]**	Zircon U-Pb CA-ID-TIMS age from a <0.1 m thick micaceous siltstone bed within the second dolostone marker unit of the lower Wood Canyon Formation, Spring Mountains, Nevada (sample L2005-27.1).	(*30*)

	Interpreted as a maximum depositional age. Five (of seven) single grain analyses (MSWD = 0.8; probability of fit [p(χ^2)] = 0.52).	
533 Ma	*Zircon U-Pb ID-TIMS age from bed 5 of Meishucun section (Zhongyicun Member). Age in abstract form only and no information pertaining to uncertainty or procedural laboratory techniques (including number of single grain analyses, air vs chemical abrasion, tracer etc.) exist in published form to our knowledge. Likely CA-ID-TIMS. Whilst we do not include this age in our model, we note that age Model 'K' remains entirely consistent with this age. Whilst the phosphorite interval of the Zhongyicun Member at Meishucun does not afford any useful, detailed chemostratigraphic correlation potential at present, this age is consistent with a young BACE age [e.g., ref.* (*30*)*], and an old (5p) correlation for the ZHUCE (Dahai Member) after ref.* (*66*)*.*	*Age provided in ref.* (*109*) *citing ref.* (*120*)
**534.6 ± 0.5**	Zircon air-abrasion ID-TIMS age for ultra-potassic trachyrhyolite porphyry cobbles in a fluvial conglomerate of the lower Tyuser Formation in the Kharaulakh ranges, lower Lena River. *Age requires updating using CA-ID-TIMS.*	(*121*)
**537.95 ± 0.28 (0.36) [0.68]**	Zircon U-Pb CA-ID-TIMS age of a tuff bed in the upper Nomtsas Formation exposed on the Neint Nababeep Plateau, northwest South Africa (sample E1841-62.1). Five (of five) single grain analyses (MSWD = 0.98, n = 5).	(*63*)
**538.04 ± 0.14 (0.27) [0.63]**	Zircon U-Pb CA-ID-TIMS age of a tuff bed in the lower Nomtsas Formation exposed on the Neint Nababeep Plateau, northwest South Africa (sample E1843). Seven (of eight) single grain	(*63*)

	analyses (MSWD = 1.4, n = 7).	
**538.568 ± 0.093 (0.17) [0.60]**	Zircon U-Pb CA-ID-TIMS age of a tuff bed in the lower Nomtsas Formation exposed on the Neint Nababeep Plateau, northwest South Africa (sample E1832-0). Five (of eight) single grain analyses (MSWD = 0.95, n = 5).	(*63*)
**538.58 ± 0.19 (0.24) [0.62]**	Zircon U-Pb CA-ID-TIMS age of a tuff bed in the lower Nomtsas Formation exposed on Farm Swartkloofberg, west of Swartpunt (sample 17SWART7, ash 6, equivalent to 92-N-1 of (*122*). Three single grain analyses (MSWD = 0.10, n = 3)	(*62*, *122*)
**538.99 ± 0.21 (0.25) [0.63]**	Zircon U-Pb CA-ID-TIMS age of the highest ash bed exposed in the upper Spitskop Member of the section on Farm Swartpunt (sample 15UNA20, ash 5). Three single grain analyses (MSWD = 2.2, n = 3). Ongoing correlation uncertainty between dated ash beds on the Neint Nababeep Plateau and in the vicinity of farms Swartpunt and Nord Witputz.	(*62*)
**538.74 ± 0.17 (0.25) [0.63]**	Zircon U-Pb CA-ID-TIMS age of a tuff bed in the Spitskop Member (Urusis Fm) exposed on the Neint Nababeep Plateau, northwest South Africa (sample L1835-0). Six (of eight) single grain analyses (MSWD = 1.1, n = 6).	(*63*)
**539.40 ± 0.23 (0.35) [0.66]**	Zircon U-Pb CA-ID-TIMS age of a 10cm-thick bed of sandy, hematite-rich dolostone interpreted as a diagenetically altered tuffaceous horizon in the upper La Ciénega Formation (top of Unit 3 at Cerro Clemente), above a laterally reproducible negative excursion correlated with the global BACE (sample CC1801-138). Interpreted conservatively as a maximum depositional age. Six single grain zircon	(*123*)

	fragments (of 10) (MSWD = 1.05, n = 6). We consider this level to be slightly younger than the age itself, to maintain consistency with ages and carbon isotope profile of the Swartpunt section.	
539.4 ± 2.9	*SHRIMP U-Pb age of bentonites, Zhongyicun Member of the Zhujiaqing Formation, bed 5 at Meishucun section, Yunnan Province, South China. Note, age updated by ID-TIMS (120), see above), but data in abstract form only. We prefer the younger age provided by (120) for this age model in agreement with (109) However, uncertainty in some details of this age derivation (noted above) mean that it is not included in figures.*	(*114*)
**539.41 ± 0.23 (0.33) [0.66]**	Zircon U-Pb CA-ID-TIMS age of a tuff bed in the Spitskop Member (Urusis Fm) exposed on the Neint Nababeep Plateau, northwest South Africa (sample L1834-31.5). Six (of six) single grain analyses (MSWD = 0.60, n = 6).	(*63*)
**539.63 ± 0.15 (0.27) [0.64]**	Zircon U-Pb CA-ID-TIMS age of a tuff bed in the Huns? Member (Urusis Fm) exposed on the Neint Nababeep Plateau, northwest South Africa (sample E1680-750). Four (of five) single grain analyses (MSWD = 0.47, n = 4).	(*63*)
**539.64 ± 0.19 (0.23) [0.62]**	Zircon U-Pb CA-ID-TIMS age of tuff bed in upper Spitskop Member in middle of section on Farm Swartpunt (sample 15UNA19, ash 4). Four single grain analyses (MSWD = 0.46, n = 4).	(*62*)
**539.52 ± 0.14 (0.20) [0.61]**	Zircon U-Pb CA-ID-TIMS age of tuff bed in upper Spitskop Member in middle of section on Farm Swartpunt (sample 15UNA18, ash 3). Five single grain analyses (MSWD = 1.4, n = 5).	(*62*)
**539.58 ± 0.34 (0.37) [0.68]**	Zircon U-Pb CA-ID-TIMS	(*62*)

	age of tuff bed in upper Spitskop Member in middle of section on Farm Swartpunt (sample 15UNA17, ash 2). Six single grain analyses (MSWD = 0.44, n = 6)	
**540.095 ± 0.099 (0.17) [0.60]**	Zircon U-Pb CA-ID-TIMS age of an ash bed in the upper Spitskop Member at the base of the section on Farm Swartpunt [sample 15UNA22, ash 1, equivalent to 94-N-11 of (*122*)]. Five single grain analyses (MSWD = 1.7, n = 5). Ongoing uncertainty with respect to possible zircon reworking in the vicinity of Farm Swartpunt suggested by ref. (*63*)	(*62*, *122*)
**541.00 ± 0.13 (0.21) [0.81]**	Zircon U-Pb CA-ID-TIMS age from Ara Group (A4 Member, 3045m depth in Birba-5 well, SOSB), Oman (sample BB-5). 8 concordant single grain analyses of 18 total (MSWD = 1.0, n = 8). This age is no longer considered to accurately constraint the temporal position of the 1n/BACE (see ref. (*30*) for details).	(*124*)
**541.85 ± 0.75 (0.77) [0.97]**	Zircon U-Pb age for ash bed at top of Tamengo Formation, Brazil. Dated via U-Pb CA-ID-TIMS using the ET535 tracer. Cluster of the five youngest concordant analyses (MSWD = 3.3, n = 5 out of 11)	(*89*)
**542.33 ± 0.11 (0.19) [0.79]**	Zircon U-Pb CA-ID-TIMS age from Ara Group (A3 Member, 9m below top of A3 carbonate unit, 2194.4m depth in Mukhaizna-11 well), Oman (sample MKZ-11B). 8 concordant single grain analyses of 16 total (MSWD = 0.50, n = 8)	(*124*)
**542.37 ± 0.28 (0.32) [0.68]**	Zircon U-Pb age for ash bed at top of Tamengo Formation, Brazil. Dated via U-Pb CA-ID-TIMS using the ET535 tracer. Cluster of four concordant analyses (MSWD = 0.68, n = 4 out of 8)	(*89*)
**542.54 ± 0.45 (0.53) [1.13]**	Zircon U-Pb CA-ID-TIMS age from top of Fara Formation (Ara A2-A3	(*124*)

	equivalent), Oman (sample WB.01.1). 4 concordant single grain analyses of 10 total (MSWD = 2.6, n = 4)	
**542.65 ± 0.15 (0.21) [0.62]**	Zircon U-Pb CA-ID-TIMS age of a tuff bed in the Nasep Member (Urusis Fm) exposed in the Witputs Sub-basin, southern Namibia (sample L1940B). Five (of five) single grain analyses (MSWD = 1.0, n = 5).	(*63*)
542.68 ± 1.25 (2.80)	*Zircon Pb-Pb ID-TIMS age of an ash bed in the lower Spitskop Member on Farm Nord Witputs (sample 91-N-1 or, alternatively, BZS-7). Air abrasion age of eight single grain and small multigrain fractions.* (*122*) *originally reported a weighted mean* $^{207}Pb/^{206}Pb$ *crystallization age of 545.1 ± 0.70 Ma (MSWD = 0.22, n = 8). Age superseded by recent data. Ongoing correlation issues and possible ash bed reworking in vicinity of Farm Swartpunt [e.g.,* (*63*, *125*)*] requires future detailed investigation.*	(*122*)*, recalculated in* (*97*)
**542.90 ± 0.12 (0.20) [0.80]**	Zircon U-Pb CA-ID-TIMS age from Ara Group (A3 Member, 3m above base of A3 carbonate unit, 3988.3m depth in Minha-1 well), Oman (sample Minha-1A). 8 concordant single grain analyses of 17 total (MSWD = 0.62, n = 8)	(*124*)
**543.40 ± 3.5**	SIMS zircon U-Pb age from an ash bed 45m above the base of the Baimatuo Member, Zhoujiaao section, southern margin of the Huangling anticline, 3 Gorges Area. Age constrains plateau in $\delta^{13}C_{carb}$ at ~3‰.	(*126*)
543.9 ± 0.24 Recalculated to 542.8 ± 1.30 Ma by (*109*) ***and interpreted as the maximum age of the unit by*** (*109*)	*Zircon U-Pb ID-TIMS (air abrasion), Kessyusa Group (Syhargalakh Formation) volcanic breccia of the Tas-Yuryakh volcanic complex (Khorbusuonka River). These zircons have not been re-analysed using the updated chemical abrasion methodology and, as stated in ref.* (*109*)*, have lost Pb. We anticipate substantial*	(*121*)

	modification to this age after future re-analysis. If taken as a minimum age for the top of the Turkut Formation, this may imply correlation of the negative excursion at the top of the Turkut Formation (e.g. at the Olenek River section) with the A0 excursion. However, given the presence of morphologically simple anabaritids (Cambrotubulus) at the base of the Turkut Formation, this seems unlikely at present. We prefer to correlate the Turkut negative excursion with the 1n/BACE onset ('0n'), which is also consistent with the interpretation of ref. (*109*) *for a maximum age of 542.8 ± 1.30 Ma for the Tas-Yuryakh volcanic complex, and a minimal hiatus separating the Syhargalakh Fm from the underlying Turkut Formation. However, this correlation remains poorly constrained, especially when considering the possibility for variable completeness of the upper Turkut Formation associated with the karstic unconformity, generally low and scattered* $\delta^{13}C_{carb}$ *data throughout the Turkut and underlying Khatyspyt formations, and associated difficulty in confident regional* $\delta^{13}C_{carb}$ *correlation between sections [e.g., see SI of ref.* (*72*)*].*	
**545.27 ± 0.11 (0.18) [0.61]**	Zircon U-Pb CA-ID-TIMS age of a tuff bed in the Nudaus Formation, exposed in the Witputs Sub-basin, southern Namibia (sample L1941). Six (of seven) single grain analyses (MSWD = 0.63, n = 6).	(*63*)
**546.25 ± 0.19 (0.27) [0.64]**	Ash bed in the Jiucheng Member, 471m above the base of the Dengying Formation at Yinchangpo section (sample 14YCP02). Zircon U-Pb CA-ID-TIMS age (n = 5 of 12). Originally dated via SIMS with weighted mean $^{207}Pb/^{206}Pb$	CA-ID-TIMS age (*28*) updated from (*127*)

	age of 546.3 ± 2.70 (3.80) (MSWD = 0.58, n = 44 of 50). Constrains a maximum depositional age for the base of the overlying Baiyanshao Member at Yinchangpo section.	
**546.72 ± 0.21 (0.29) [0.89]**	Zircon U-Pb CA-ID-TIMS age of tuff bed from Ara Group (middle of A0 Member, 3847m depth in Asala-1 well), Oman (sample Asala-1 c21). 8 concordant single grain analyses of 12 total (MSWD = 0.92, n = 8).	(*124*)
**547.23 ± 0.28 (0.36) [0.96]**	Zircon U-Pb CA-ID-TIMS age of tuff bed from the Fara Formation (200m above the base of the formation), Oman (sample WB.01.2). Considered to predate A0 Member. 8 concordant single grain analyses of 17 total (MSWD = 1.3, n = 8)	(*124*)
**547.36 ± 0.23 (0.31) [0.91]**	Zircon U-Pb CA-ID-TIMS age from 8 single grain analyses (sample 94-N-10B). Lower Hoogland Member, Zaris Formation, Kuibis Subgroup, Nama Group, Namibia. 8 single grain analyses (MSWD = 1.4, n = 8).	(*124*). Ref. (*97*) report age of 547.32 ± 0.31 (0.65) Ma.
**550.14 ± 0.16 (0.24) [0.63]**	Zircon U-Pb CA-ID-TIMS age of an ash in Doushantuo Member IV (Miaohe Member) at Jijiawan section, Hubei Province (Yangtze Gorges area), South China (sample 16JJW-3). Ash bed is 85cm below base of Dengying Formation (Hamajing Member). Six concordant (of 14) single grain analyses (MSWD = 1.9).<br><br>Original ages in ref. (*128*) for same ash bed (sample JIN04-2): U-Pb concordia age of 551.07 ± 0.61 Ma (MSWD = 0.48), and weighted mean $^{207}Pb/^{206}Pb$ 550.55 ± 0.75 Ma (MSWD = 0.48). Age recalculated in ref. (*28*) using two concordant (of ten total) single grain analyses. All ten zircons yield weighted mean $^{207}Pb/^{206}Pb$ age of 548.09 ± 2.61 Ma.	Age updated in ref. (*28*) from initial age of ref. (*128*).

**552.96 ± 0.19 (0.30) [0.66]**	Zircon U-Pb CA-ID-TIMS age of a tuff bed in the lower part of the Zimnegory Formation, Valdai Group, White Sea area (sample WhiteSeaAsh). Five concordant (of 11) single grain analyses. Sample previously dated at 555.3 ± 0.3 Ma (*129*), recalculated to 552.85 ± 0.77 (2.62) ($^{207}Pb/^{206}Pb$) by ref. (*28*).	(*28*)
**554.29 ± 0.14 (0.22) [0.63]**	Zircon U-Pb CA-ID-TIMS age for an ash bed 3.8m above the phosphorite layer at the base of the Jiucheng Member at Xiaolantian section, eastern Yunnan (sample 14CJ07, MSWD = 0.8, n = 7 of 10). Age implies highly condensed sedimentation, cryptic hiatus within lower Jiucheng member, reworking during deposition, or incorporation of xenocrystic materials in the magmatic environment (see age of sample 14YCP02). See discussion in (*28*). Modified age Model 'K' (herein) assumes that this age approximates the depositional age of the lower Jiucheng Member.	Updated from original SIMS age of (*127*) by (*28*)
**555.18 ± 0.3 (0.34) [0.70]**	Zircon U-Pb age for ash bed in the upper Bocaina Formation, Brazil. Dated via U-Pb CA-ID-TIMS using the ET535 tracer. (MSWD = 1.6, n = 8 out of 8)	(*89*)
**556.26 ± 0.21 (0.25) [0.65]**	Zircon U-Pb CA-ID-TIMS age of the dolostone/chert boundary of the basal Liuchapo Formation, Nangao section (samples 17WA05). Five concordant (of 9 total) single grain analyses (MSWD = 0.2, n = 9).	(*28*)
**556.38 ± 0.14 (0.27) [0.65]**	Zircon U-Pb CA-ID-TIMS age of the dolostone/chert boundary of the basal Liuchapo Formation, Wengxiu section (samples 17GZWX01). Eight concordant (of 10 total) single grain analyses (MSWD = 1.8, n = 8).	(*28*)
**556.6 ± 6.4**	Zircon U-Pb CA-ID-TIMS age from 4 single grain analyses (sample 846).	(*130*)

	Hanging Rocks Formation (Maplewell Group, Charnian Supergroup).	
**556.78 ± 0.10 (0.18) [0.62]**	Zircon U-Pb CA-ID-TIMS age from bentonite bed in middle Mohylivska Formation, Podolia, Ukraine (sample B1b). All five zircons from bentonite B1 are concordant, yielding a statistically equivalent weighted mean age (MSWD = 2.2, n = 5)	(*131*)
**557 ± 3**	SHRIMP zircon U-Pb age for ash bed in dolostone of the Liuchapo Fm at Fanglong section, Guizhou province. Immediately above recovery from a negative $\delta^{13}C_{carb}$ excursion.	(*132*)
**557.28 ± 0.14 (0.22) [0.63]**	Zircon U-Pb CA-ID-TIMS age of tuff bed at the base of the Verkhovka Formation, Valdai Group, White Sea area (sample 9607-1601). Six concordant (of 9 total) single grain analyses (MSWD = 1.6).	(*28*)
**561.85 ± 0.34 (0.66) [0.89]**	Zircon U-Pb CA-ID-TIMS age from 7 concordant (of 12) single grain analyses (sample 912). Bradgate Formation (Maplewell Group, Charnian Supergroup). (MSWD = 1.2)	(*130*)
**562.5 ± 1.1**	Zircon U-Pb CA-ID-TIMS age from 7 concordant single grain analyses (MSWD = 0.34). 27m below the top of the Trepassey Formation, Shingle Head, Mistaken Point ecological reserve, Newfoundland (sample N10-SH6B). *Detailed stratigraphic mapping and reassessment by* (*133*) *places this ash bed in the lower Fermeuse Formation. See* (*133*) *for discussion of additional uncertainties associated with the age of sample N10-SH6B. The maximum age of the lower Fermeuse Formation suggested by sample SH-2 of* (*133*) *(see below) is 564.13 ± 0.20 Ma.*	(*134*)
**562.7 ± 3.8**	Re-Os age lower Buah Fm, Well M. Initial $^{187}Os/^{188}Os$ = 0.68 ± 0.01. (2σ age, MSWD	(*135*)

	= 1.40, n = 7)	
**564.13 ± 0.20 (0.25) [0.65]**	Zircon U-Pb CA-ID-TIMS age of tuff bed in the lower Fermeuse Formation (Shingle Head surface), Mistaken Point Ecological Reserve, Newfoundland (sample SH-2). Six single grain analyses (MSWD = 1.5, n = 6)	(*133*)
**564.71 ± 0.63 (0.65) [0.88]**	Zircon U-Pb CA-ID-TIMS age of tuff bed immediately atop the 'Pizzeria' in the Trepassey Formation, Long Cove, Mistaken Point Ecological Reserve, Newfoundland (sample LC-1). Two single grain analyses (MSWD = 0.69, n = 2 of 11)	(*133*)
**565.00 ± 0.16 (0.22) [0.64]**	Zircon U-Pb CA-ID-TIMS age of tuff deposit immediately above 'E' surface of upper Mistaken Point Formation (~60m below top of Formation), Mistaken Point Ecological Reserve, Newfoundland (sample MP-14). Four concordant single grain analyses (MSWD = 1.2, n = 4)	(*133*)
**565.22 ± 0.33 (0.65) [0.89]**	Zircon U-Pb CA-ID-TIMS age from 2 concordant (of 5) single grain analyses (sample 907). Beacon Hill Formation (Maplewell Group, Charnian Supergroup). (MSWD = 0.42, n = 2)	(*130*)
566.25 ± 0.35 (0.48) [0.77]	*Zircon U-Pb CA-ID-TIMS age of Mistaken Point Formation, Newfoundland (sample MPMP33.56). Five single grain analyses (MSWD = 1.3, n = 5). Analyses show degree of discordance, and the age of the same ash horizon has been updated in* (*133*) *(their sample MP-14, see above). We favour the concordant age of* (*133*) *sample MP-14.*	(*136*)
**566.9 ± 3.5**	Re-Os age from an organic-rich microbialite in the lower part of the Gametrail Formation, Wernecke Mountains at section JB1714 (Goz D, sample JB1714-31.3m). Immediately above carbonate interval that hosts a negative carbon isotope	(*49*)

	excursion that has been regionally correlated with the global Shuram excursion. Dated microbialite approximates 0 permil recorvery of carbonate carbon isotope data. Initial $^{187}Os/^{188}Os = 0.33 \pm 0.003$. (2σ age, MSWD = 1.04, n = 6)	
**567 ± 3.9**	Zircon U-Pb age of volcanic tuff from Sylvitsa Group (Perevalok Formation), Krutaya Gora section, Us'va River, central Urals (sample 09-03-15). (MSWD = 1.14, n = 16). *Age shown in figures but lack of detailed analytical methods provided in original publication.*	(*137*)
**567.3 ± 3.0**	Re-Os age upper Unit PH4, inferred equivalent to Blueflower Fm (sample A1707). 16m above contact with Gametrail Fm, Coal Creek Section, Ogilvie Mountains. Initial $^{187}Os/^{188}Os = 0.61 \pm 0.04$. (2σ age, MSWD = 0.81, n = 6)	(*135*)
**567.63 ± 0.21 (0.26) [0.66]**	Zircon U-Pb CA-ID-TIMS age of tuff bed in the middle Briscal Formation (~110m above the base of the Formation) overlying the 'Brasier Surface', Mistaken Point Ecological Reserve, Newfoundland (sample BRS-1). Five single grain analyses (MSWD = 2.1, n = 5)	(*133*)
**569.08 ± 0.45 (0.73) [0.94]**	Zircon U-Pb CA-ID-TIMS age from 2 concordant (of 12) single grain analyses (sample 918). Bennscliffe Breccia between Blackbrook Reservoir Formation and Beacon Hill Formation (Charnian Supergroup). (MSWD = 0.8, n = 2).	(*130*)
570.94 ± 0.38 (0.46) [0.77]	*Zircon U-Pb CA-ID-TIMS age of tuff bed in upper Drook Formation, Newfoundland (sample Drook-2). Five single grain analyses (MSWD = 0.33, n = 5). Despite ongoing uncertainty in the age of this ash layer, we favour the model of (133) based on maintenance of stratigraphic superposition using more*	(*136*)

	recent ages from the upper Drook Formation (their sample DRK-10) and overlying lower Briscal Formation (their sample DRK-1). Accordingly, the age of sample Drook-2 is not included in our age model despite concordant single grain analyses and low uncertainty.	
**571.38 ± 0.16 (0.25) [0.66]**	Zircon U-Pb CA-ID-TIMS age of tuff bed in the basal Briscal Formation (~20m above top of Drook Formation), Daley's Cove, Mistaken Point Ecological Reserve, Newfoundland (sample DRK-1). Eight single grain analyses (MSWD = 2.0, n = 8)	(*133*)
**574.0 ± 4.7**	Re-Os age upper Nadaleen Fm, J1719. Initial $^{187}Os/^{188}Os$ = 0.60 ± 0.01. (2σ age, MSWD = 0.75, n = 8)	(*135*)
**574.17 ± 0.19 (0.24) [0.66]**	Zircon U-Pb CA-ID-TIMS age of Drook Formation (~25m below top of Formation), 'Pizza Disc Bed', Pigeon Cove, Mistaken Point Ecological Reserve, Newfoundland (sample DRK-10). Nine single grain analyses (MSWD = 2.8, n = 9)	(*133*)
**575.0 ± 5.1**	Re-Os age Nadaleen Fm, J1443. Initial $^{187}Os/^{188}Os$ = 0.60 ± 0.01. (2σ age, MSWD = 1.20, n = 5)	(*135*)
**578.2 ± 5.9**	Re-Os age middle Khufai Fm, Well L. Initial $^{187}Os/^{188}Os$ = 1.15 ± 0.05. (2σ age, MSWD = 0.97, n = 7)	(*135*)
**579.88 ± 0.44 Ma (0.52) [0.81]**	Zircon U-Pb CA-ID-TIMS age of lower Drook Formation (sample NoP-0.9). Five single grain analyses (MSWD = 0.82, n = 5)	(*136*)
**580.90 ± 0.40 (0.53) [0.82]**	Zircon U-Pb CA-ID-TIMS age of Upper Mall Bay Formation (sample GCI-neg6.55). Nine single grain analyses (MSWD = 1.1, n = 9)	(*136*)
**585.7 ± 2.6 [2.8]**	Re-Os age on sample (A1606) 1m below 'Carbonate B' of the Doushantuo Formation at Wenghui section, Yangtze Gorges, South China (sample	(*28*)

	A1606). Initial $^{187}Os/^{186}Os$ = 1.81 ± 0.02. (n = 9)	
**587.2 ± 3.3 [3.6]**	Re-Os age on sample (F1404) 58m above the base of the Doushantuo Formation at Jiulongwan section, Yangtze Gorges, South China (sample F1404). Initial $^{187}Os/^{186}Os$ = 0.90 ± 0.02. (2σ age, MSWD = 1.1, n = 6)	(*28*)
**612.46 ± 0.62 (0.67) [0.94]**	Zircon U-Pb CA-ID-TIMS age (re-interpreted as a maximum deposition age for detrital zircons in a mudstone layer) at the Unit 4/5 contact of the Doushantuo Fm at Zhangcunping section, Hubei. Carbonate below the dated horizon records a negative $\delta^{13}C_{carb}$ excursion.	Original SIMS age of (*138*) updated by (*28*)
**614 ± 7.6**	Zircon U-Pb SHRIMP age of a tuff bed in the Doushantuo Formation at Wangjiagou section, Zhangcunping, Hubei (sample 7527). Tuff bed occurs between beds 3 and 4, below an erosional unconformity in the middle Doushantuo Fm (Member II). Eighteen single grain analyses. Age reported initially was 614 ± 7.6 Ma (MSWD = 2.3, n = 18).	(*139*), updated by (*97*)
**632.48 ± 1.02**	Zircon U-Pb CA-ID-TIMS age of a tuff bed in the lower Doushantuo Formation (at top of black shale unit, ~9m above the Nantuo-Doushantuo contact) at Jijiawan (Jiuqunao) section (sample YG-04-2). Three concordant (of 9 total) single grain analyses. Ref. (*128*) initially reported an age of 632.50 ± 0.48 Ma (MSWD = 0.38, n = 3).	(*128*) updated by ref. (*97*)
**634.57 ± 0.88 (0.90) [1.61]**	Zircon U-Pb CA-ID-TIMS of an ~20cm thick grey tuffaceous mudstone within the top of the Nantuo diamictite at Eshan section, eastern Yunnan (sample ES-1). Four concordant analyses (MSWD = 1.4, n = 4 of 7).	(*140*)
**635.21 ± 0.59 (0.61) [0.92]**	Zircon U-Pb CA-ID-TIMS age of a tuff deposit (~30m below the contact with the Keilberg cap dolostone) interbedded with the basinal equivalent of the Ghaub	(*141*)

	Formation glacial diamictite, Navachab section, norther Namibia (sample NAV-00-2B). Five single grain analyses (MSWD = 3.4, n = 5).	
**635.26 ± 1.07**	Zircon U-b CA-ID-TIMS age of a tuff bed at the contact surface between the lower and upper part of the cap dolomite, overlying the Nantuo glacial diamictite at the Wuhe-Gaojiaxi section, Yangtze Gorges (sample YG-04-15). Three concordant (of 18) single grain analyses. Ref. (*128*) initially reported an age of 635.23 ± 0.57 Ma (MSWD = 0.28, n = 3).	(*128*) recalculated by ref. (*97*)
**639.29 ± 0.26 (0.31) [0.75]**	Zircon U-Pb CA-ID-TIMS age of tuff deposit interbedded with the Ghaub glacial diamictite (~15m below the base of the Keilberg cap carbonate), Duurwater section, northern Namibia (sample DW-1). Middle of three ash beds. Nine single grain analyses (MSWD = 2.6, n = 9)	(*141*)
**651.69 ± 0.64 (0.73) [0.99] Ma**	U-Pb zircon CA-ID-TIMS age from a lenticular bed of laminated light-grey siltstone within dolostone of the upper Thorndike submember, 15m below the base of the Wildrose submember of the South Park Member of the Kingston Peak Formation, Panamint Range, Death Valley, California (sample L1502). Interpreted as a maximum depositional age. MSWD=1.0, n=4 of 5.	(*142*)

Table S2. Details used in outcrop/terrane area and stratigraphic thickness estimates, with associated references. *Italicized text indicates units with highly uncertain thickness estimates and/or upper/lower boundary age estimates.*

Craton/Terrane	Unit (Group/ Formation/ Member)	Acronym/ Abbreviation used in Fig. S1	Unit thickness	Additional information (e.g., max./min. age uncertainty)	References*
West Avalonia (Avalon Terrane, Newfoundland)	Brigus Formation (St Mary's Member only)		St Mary's Member (lower carbonate-rich interval ca. 2 m, clastic-dominated interval ca. 18 m, carbonate-rich interval ca. 2 m, clastic-dominated interval ca. 10 m, carbonate-rich interval ca. 2 m, clastic-dominated interval ca. 10 m, carbonate-rich interval ca. 2 m, clastic-dominated interval ca. 40 m).	St Mary's Member only, after u-Pb zircon date of 507.91 ± 0.07 (0.16) [0.55] Ma from sample RBBR-26.5.	(*143*, *144*)
	Bonavista Group (including Petley, West Centre Cove, Cuslett and Fosters Point formations)		Petley Fm (basal carbonate – ca. 2m; clastics with nodular carbonate – ca. 18 m; upper carbonate – ca. 2 m); West Centre Cove Fm (lower clastics – ca. 56 m; upper carbonate – ca. 2 m); Cuslett Formation (Member 1 and lower Member2 clastics – ca. 28 m; mid-Member2 carbonate – ca. 2 m; mid-upper Member 2 clastics with nodular carbonate – ca. 5 m; upper Member 2 = 2x carbonate beds, each ca. 3 m-thick separated by ca. 1.5 m-thick clastic interbed; lower member 3 clastics – ca. 28 m-thick, upper Member 3 carbonate – ca. 2 m-thick; lower member 4 clastics – ca. 18 m-thick, middle Member 4 carbonate – ca. 2 m-thick, upper Member 4 clastics – ca. 10 m-thick); Fosters Point Formation max. ca. 50 m		(*143*, *144*)
	Random Formation	R	estimated thickness ca. 185 m		(*145*)

	Chapel Island Formation (Chapel Island Formation, members 1-5, including: Quaco Road and Mystery Lake members)	CIF	Chapel Island Formation, Member 5: estimated thickness ca. 180 m [composite log of (*146*)]<br><br>Chapel Island Formation, Member 4: basal limestone marker bed: 0.15 m-thick, followed by 50 m clastics, 0.24 m- thick limestone 2, 35 m clastics, and finally max 0.80 m-thick limestone 3 (*147*)<br><br>Chapel Island Formation, Member 2 + Member 3 clastics: estimated thickness 575 m [composite log of (*146*)]<br><br>*Chapel Island Formation, Member 1: estimated thickness 185 m [composite log of* (*146*)*]. Max. age remains highly uncertain.*		(*146*, *147*)
	Rencontre Formation	RENC	*Rencontre Fm: estimated maximum thickness = 2.75 km* (*148*)*. Minimum and maximum ages remain highly uncertain.*		(*148*)
	Signall Hill Group (correlates to Marystown Group on southern Burin Peninsula and Musgravetown Group on Bonavista Peninsula-Western Placentia Bay)		*Estimated maximum thickness = 5.75 km. Maximum and minimum ages remain highly uncertain.*		(*149*)
	Renews Head Formation	RENEWS	*Estimated thickness = 0.8 km (based on composite log). Minimum age remains highly uncertain.*		(*149*)

	Fermeuse Formation	FERM	Estimated thickness of Fermeuse (ca. 720 m)		(*133*)
	Trepassey Formation	T	Estimated thickness of Trepassey (ca. 240 m)		(*133*)
	Mistaken Point Formation	MP	Estimated thickness of Mistaken Point (ca. 290 m)		(*133*)
	Briscal Formation	BRISCAL	Estimated thickness of Briscal (ca. 310 m)		(*133*)
	Drook Formation	DROOK	Estimated thickness of Drook (ca. 770 m)		(*133*)
	Gaskiers Formation		Based on mean thickness at Old Bonaventure (ca. 40m) and St. Mary's Bay (ca. 260m) = 150m.		(*136*)
	Additional(/summary) references				(*105*, *133*, *143*, *144*, *146*, *148–153*)
East Avalonia (England and Wales)	Caerbwdi Sandstone (including Caerfai Bay Shale) of the Caerfai Group (Pembrokeshire)<br><br>Purley Shale Formation (Nuneaton)<br><br>Lower Comley Sandstone	CAER/PUR/COM	*Caerbwdi Sandstone and Caerfai Bay Shale (max. thickness ca. 165 m). lower Comley Sandstone Fm (max. ca. 150 m), Purley Shale (max. ca. 200 m): mean = ca. 170 m. Minimum age of these units remains highly uncertain.*		

	Formation (Shropshire)				
	Home Farm Member of the Hartshill Sandstone Formation	HFM	Woodlands Member of the Hartshill Formation: dominated by dark maroon, thick-bedded subarkosic sandstone with bioturbated top – ca. 10 m.<br><br>Home Farm Member of the Hartshill Formation (simplified): basal conglomerate and calcareous sandstone with rare mudstone/siltstone/shale – max. ca. 0.98 m, phosphatised limestone conglomerate – max. ca. 0.15 m, coleoloides limestone – max. ca. 0.55 m, mudstone/siltstone/shale – max. ca. 0.28 m, coleoloides limestone with some hyolith-bearing phosphatised limestone conglomerate, sparry limestone with desiccation cracks, algal limeston, and rare mudstone/siltstone/shale interbeds – max. ca. 0.78 m		(*154*, *155*)
	Lower Hartshill Sandstone Formation (Nuneaton) and equivalent Wrekin Quartzite (Shropshire)	HART (LWR)	*Lower Hartshill Sandstone Fm (Boon's Mb – max. 40 m, Park Hill Mb – max. 55 m, Tuttle Hill Mb – lower grey to pink subarkosic sandstone interval – ca. 60 m, middle dark maroon subarkosic micaceous sandstone with grey mudstone and siltstone interbeds – ca. 10 m, upper dark maroon subarkosic sandstone – 110 m, Jee's Mb – 6 m); Wrekin Quartzite (40 m)* (*110*, *155*)*. Maximum age remains highly uncertain.*		(*110*, *155*)
	Charnian Supergroup, Maplewell Group, Hanging Rocks Formation (Charnwood Forest,	HR	*ca. 260 m. Minimum age remains highly uncertain.*		(*130*)

	Leicestershire)				
	Charnian Supergroup, Maplewell Group, Bradgate Formation (Charnwood Forest, Leicestershire)	BRAD	ca. 680 Ma		(*130*)
	Charnian Supergroup, Maplewell Group, Beacon Hill Formation (Charnwood Forest, Leicestershire)	BEACON	BB + Beacon Hill – ca. 1530 m		(*130*)
	Charnian Supergroup, Maplewell Group, Benscliffe Breccia Member of the Beacon Hill Formation (Charnwood Forest, Leicestershire)	BB	BB + Beacon Hill – ca. 1530 m		(*130*)
	Charnian Supergroup, Blackbrook Group, Blackbrook	BB Res	ca. 595 m		(*130*)

	Reservoir Formation (Charnwood Forest, Leicestershire)				
	Charnian Supergroup, Blackbrook Group, Ives Head Formation (Charnwood Forest, Leicestershire)	IVES HEAD	ca. 860 m		(*130*)
	Additional(/summary) references				(*106*, *110*, *130*, *143*, *154*, *156*)
South China (Yangtze Platform to open slope, west)	Wulongqing Formation	WULO	*estimated max. thickness ca. 55 m at Xindian-Xianfeng section. Precise minimum age remains uncertain.*		(*157*)
	Hongjingshao Formatino	H	estimated max. thickness ca. 100 m at Xindian-Xianfeng section		(*157*)
	Yu'anshan Formation	YU'AN	Max. thickness ca. 200 m (Xiaotan section)		(*158*, *159*)
	Shiyantou Formation		Max. thickness ca. 200 m (Xiaotan section). Basal phosphatic beds at Meishucun ≤ ca. 2 m		(*159*, *160*)
	Zhujiaqing Formation, Dahai Member	ZHUJIAQI	average thickness – ca. 40 m (based on 3 sections)		

	Zhujiaqing Formation, Zhongyicun Member	ZHUJIAQI	average thickness – ca. 35 m (based on 5 sections)		
	Zhujiaqing Formation, Daibu Member	ZHUJIAQI	average thickness – ca. 50 m (based on 3 sections)		
	Dengying Formation, upper carbonate-dominated interval (e.g., Beiwan and Baiyanshao members)	BEIWAN/<br>BAIYANSHA	*average thickness estimated at ca. 135 m (based on 8 sections). Minimum age of upper carbonate-dominated Dengying Formation remains uncertain.*		
	Dengying Formation, middle clastic/carbonate interval (e.g., Gaojiashan and Jiucheng members)	JIUCH/<br>GAO	average thickness estimated at ca. 40 m (based on 4 sections)		
	Dengying Formation, lower carbonate-dominated interval (e.g., Donglongtan and Algal Dolomite members)	A DOL/DONG	*Highly variable thickness across the platform-slope (min. = ca. 15 m at Weng'an, max. = ca. 270 m at Feidatian-Luxishao/Dongdahe section), with tentative average thickness estimated at ca. 90 m (based on 12 sections), however, note that underlying and/or overlying contacts are not always observed, so this may approximate a minimum thickness estimate*		

	Doushantuo Formation		average thickness of interval with BAINCE – ca. 6 m; thickness of interval with 'Khufai peak' (shallow dolostone with phosphatic interbeds) is highly variable (at Weng'an – ca. 17 m, at Feidatian-Luxishao/Dongdahe – ca. 30 m, at Sishang – ca. 14 m), with tentative average thickness of ca. 20 m; lower DOUNCE interval of deeper carbonate ranges from ca. 5.5 m (e.g., Weng'an) to ca. 25 m (e.g., Sishang), with tentative average thickness estimated at ca. 10 m (based on 7 sections); upper DOUNCE interval (prior to recovery) with shallower marine carbonate is most commonly ≤1 m where preserved, but occasionally up to > 25 m (e.g., Feidatian-Luxishao/Dongdahe section), with tentative average thickness estimated at ca. 5 m (based on 4 sections); DOUNCE recovery interval with deeper clastic/carbonate lithologies also highly variable across the platform-slope and basin, commonly ≤1 m, but may approach ca. 10 m (e.g., Feidatian-Luxishao/Dongdahe section), with tentative average thickness estimated at ca. 5 m (based on 4 sections).		
South China (Yangtze Gorges area)	Tianheban Formation	TIA	Thickness in core ca. 100 m		(*161–163*)
	Shipai Formation	S	Thickness in core ca. 190 m; thickness at Jiuqunao-Wangjiaping ca. 160 m. Average thickness ca. 175 m.		(*161–164*)
	Shuijingtuo Formation		Thickness in core and at Wangzishi section ca. 70 m		(*161–163*, *165*)
	Yanjiahe Formation	YANJIA	Average thickness ca. 30 m (based on 4 sections).		

	Dengying Formation, Baimatuo Member		Average thickness of Baimatuo Member, where lower and upper boundaries are reported, ca. 150 m (based on 3 sections). *Minimum age of Baimatuo Member remains uncertain.*		
	Dengying Formation, Shibantan Member	SHIB	Average thickness of Shibantan Member, where lower and upper boundaries are reported, ca. 85 m (based on 3 sections).		
	Dengying Formation, Hamajing Member		Hamajing Member thickness is variable across the Huangling anticline due to differences in sections completeness associated with possible slumping in western exposures and karstic upper boundary. Only section thicknesses in the central and easter Huangling anticline are considered, where base and top of the Hamajing Member are exposed and where slumping has not been reported. At these sections, the average thickness of the Hamajing Member ca. 57 m (based on 6 sections).		
	Doushantuo Formation		In sections that preserve the BAINCE, shallow water carbonates that record the BAINCE downturn have average thickness ca. 8 m (based on 7 sections where this lithological transition has been recorded). However, the lithological expression of this d13C excursion varies between sections of the Huangling anticline, with some sections preserving the full excursion in thinly interbedded dolostones and shales, with little evidence for a shallow carbonate dominated downturn. Deeper carbonate-dominated recovery from BAINCE has average thickness ca. 7 m (based on 5 sections where this lithological transition has been recorded). Shallow marine carbonate interval recording ‘Khufai peak’ and DOUNCE downturn has average thickness ca. 23 m		

			(based on 15 sections). Deeper marine carbonate (commonly limestone) that records DOUNCE nadir has average thickness ca. 25 m (based on 14 sections). Clastic-dominated interval that records DOUNCE recovery has average thickness ca. 5 m (based on 16 sections – does not include slumped interval on west side of Huangling anticline).		
South China (Yangtze Block, southeastern openly connected deep basin)	Dachenling Formation	DA	*Balang and Dachenling formations: average thickness ca. 95 m (based on just 2 sections). However, note that the precise 510 Ma level within these formations remains uncertain, and so the volume calculations certainly represent maximum possible estimates.*		
	Niutitang and Hetang formations		*Niutitang and Hetang formations: basal Niutitang phosphorite ca. 2 m. Overlying chert average thickness ca. 90 m (based on 5 sections where the upper and lower boundaries are observable). Maximum age of Niutitang/Hetang formations remains uncertain due to highly diachronous lower boundary.*		
	Liuchapo and Piyuancun formations		*Liuchapo and Piyuancun formations: basal Liuchapo dolostone interval ca. 10 m (based on 4 sections). Overlying chert average thickness ca. 50 m (based on 9 sections that record upper and lower formation boundaries). Minimum age of Liuchapo/Piyuancun formations remains uncertain due to highly diachronous boundary of e.g., Liuchapo/Niutitang.*		
	Doushantuo and Lantian formations		Doushantuo Formation: Interval thought to correspond with the BAINCE at Siduping section ca. 3 m. Interval of interbedded deep marine		

			carbonate that records the 'Khufai peak' and DOUNCE downturn average thickness ca. 25 m (based on 2 sections). Deep marine carbonates and clastics (including Lantian Formation Member III) that record the DOUNCE interval and onset of recovery, average thickness ca. 26 m (based on 7 sections).		
				Additional(/summary) references	(*60*, *66*, *94*, *157*, *158*, *166–170*)
Laurentia (Selwyn/Ogilvie Mountains)	Gull Lake Formation	GL	*Maximum thickness ca. 1050 m* (*171*)*. However, 510 Ma level within this Formation remains uncertain, and so the volume calculations certainly represent a maximum possible estimate. Archaeocyathid-bearing limestone conglomerate in the lower Gull Lake Formation* (*172*) *may suggest a minimum age coincident with lower Stage 4 (pre-Sinsk extinction event). However, no information could be found on constituent genera/species of the archaeocyath assemblage.*		(*171*, *172*)
	Narchilla Formation		Ranges in thickness from ca. 300 to 830 m thick in the Selwyn Basin (*171*).		(*171*)
	Last Chance (PH4)		Average thickness (Coal Creek and Tango Tarn) ca. 160 m.		
	PH3		*The BAINCE has not been recorded in the siliciclastic upper Sheepbed Formation (Unit PH3 of the Coal Creek Inlier and at Tango Tarn), and so this interval may either represent a hiatus below the lower PH4 sequence boundary, or ongoing siliciclastic deposition* (*173*)*.*		(*173*)

Laurentia (Mackenzie Mountains)<br><br>*See ref. (49) for detailed discussion of ongoing complications in robust regional litho- and chemostratigraphic correlation of the Sekwi Brook succession. Given the area covered by the Mackenzie Mountains, this correlation issue becomes extremely important for observed rock volume through time (especially associated with the Gametrail anomaly), and associated biostratigraphy.*	Sekwi Formation		Approximately 1 km of the Sekwi Formation can be calibrated into this interval based on global d13Ccarb and biostratigraphic correlation (*94*, *174*).		(*94*, *174*)
	Vampire Formation		Thickness ca. 300 m, estimated based on composite measured section of (*175*, *176*).		(*175*, *176*)
	Backbone Ranges Formation	BBR	*Max. thickness ca. 560 m, estimated based on composite measured section of* (*176*)*. Uncertain maximum age for Backbone Ranges Formation.*		(*176*)
	Ingta Formation	ING	Max. thickness ca. 270 m (*175–177*); thickness follows measured sections J1125, S1306 and S1305 of ref. (*178*)		(*175–178*)
	Risky Formation		At June Lake and Sekwi Brook south, the Risky Formation is noted to be on the order of ca. 100 m thick (*173*, *175–177*), but thins eastward to ca. 30 m (*179*).		(*173*, *175–177*, *179*)
	Blueflower Formation		*Blueflower Formation at Sekwi Brook: Lower interval of shallow marine carbonate ca. 15 m; overlying interval of thin bedded micrite ca. 130 m; remaining clastic-dominated Blueflower average thickness ca. 500 m (based on 3 sections in the vicinity of Sekwi Brook)* (*173*)*. Precise temporal extent of the Blueflower Formation remains uncertain.*		(*173*)
	Gametrail Formation		Gametrail Formation at Sekwi Brook: Lower interval of thin bedded micrite ca. 40 m. (*173*); upper Gametrail shallow marine carbonate ca. 130	Note ongoing uncertainty in precise	(*49*, *173*)

			m.	chemostratigraphic correlation of the Gametrail Formation at Sekwi Brook to the Shuram excursion interval [see ref. (*49*)].	
	Nadaleen Formation		*The BAINCE has not been recorded in the siliciclastic upper Sheepbed Formation, and so this interval may either represent a hiatus below the Nadaleen Formation boundary, or ongoing siliciclastic deposition in the upper Sheepbed* (*173*, *180*). *'Khufai peak' recorded in Nadaleen Formation (formerly June Beds) at Sekwi Brook ca. 500 m.*		(*173*, *180*)
Laurentia (Wernecke Mountains)<br><br>*Nadaleen River area (2000 km^2) considered separately for key intervals that show lithological distinction (e.g., Shuram excursion interval in Nadaleen River area is dominated by limestone whereas sections to the north are dominantly dolostone).*	Illtyd Formation		The Illtyd Formation ca. 950 m does not immediately overlie the Vampire Formation (which is itself overlain by carbonates of the Sekwi Formation). However, the Illtyd Formation (NW Wernecke Mountains) has been studied more extensively, includes measured sections, and can be directly correlated in time with the Sekwi Formation based on biostratigraphic information (*181*).		(*181*)
	Vampire Formation		*Like the underlying Backbone Ranges Formation, there appears to be limited measured section information for the Vampire Formation in the Wernecke Mountains (relative to the Mackenzie Mountains). Here, the maximum thickness of the Vampire Formation is estimated at ca. 85 m based on the average thickness of the interval dominated by siltstone/shale/silty very fine sandstone that overlies the quartzite-dominated interval, noted as*		(*182*)

			'Vampire Formation' in sections 7-10 of (*182*).		
	Backbone Ranges Formation	BBR	*There appears to be very little information on the lateral extent and thickness of the Backbone Ranges Formation in the Wernecke Mountains. Ref.* (*178*) *note that the Goz D section sits stratigraphically beneath a thick shallow water sandstone-dominated unit reminiscent of the Backbone Ranges Formation elsewhere. Here, the maximum thickness of the Backbone Ranges Formation is estimated at ca. 140 m based on the thickness of the lower quartzite dominated interval noted as 'Vampire Formation' at sections 7-10 of ref.* (*182*)*. This interval begins above the 40 m thick shale interval suggested by ref.* (*178*) *to correspond with the Ingta Formation. Highly uncertain maximum age for Backbone Ranges Formation.*		(*178*, *182*)
	Ingta Formation	ING	Thickness estimates based on description of section J1218 (GozD) in supplementary information of ref. (188). Lower carbonate unit (with upper phosphatic SSF-bearing horizon) ca. 30 m, upper black shale interval ca. 40 m.		(*178*)
	Risky Formation		Risky Formation (north of Nadaleen River area): Average thickness ca. 65 m (based on 5 sections) (*173*).		(*173*)
	Algae Formation		*Nadaleen River area: Algae Formation average thickness ca. 190 m [3 sections,* (*180*)*]. Maximum age for the lower Algae Formation remains uncertain.*	Regional outcrop in the Nadaleen River area, southern Wernecke Mountains	(*180*)
	Blueflower		As with the June Beds/Nadaleen and Gametrail		(*173*, *180*)

	Formation		formations, the Blueflower Formation thickens from north to south in the Wernecke Mountains. Average thickness (including in the Nadaleen River/Rackla area) ca. 300 m [based on 6 sections, (*173*, *180*)].		
	Gametrail Formation		Average thickness ca. 200 m (based on 14 sections, including what has previously been described as the lower Blueflower carbonate). (*49*, *180*).		(*49*, *180*)
	Nadaleen Formation		*The BAINCE has not been recorded in the siliciclastic upper Sheepbed Formation, and so this interval may either represent a hiatus below the Nadaleen Formation boundary, or ongoing siliciclastic deposition in the upper Sheepbed* (*173*, *180*). *Interval of the Nadaleen Formation that records the 'Khufai peak' and Shuram downturn thickens from Goz A (ca. 30 m) → NE Profeit (ca. 80 m). In Nadaleen River area, the Rackla (Nadaleen Type section) section in this interval is ca. 660 m.* (*173*, *180*)		(*173*, *180*)
Laurentia (Mexico)<br><br>Average unit thicknesses: (*123*, *183*, *184*)	Puerto Blanco Formation		ca. 430 m. *Minimum age of measured interval remains uncertain.*		
	Cerro Rajón Formation	CERRO RAJ	ca. 270 m		
	La Ciénega Formation	LA CIÉ	ca. 175 m. Age model 'K', adopted herein, is consistent with the timing of 1n/BACE onset prescribed by ref. (*30*).		
	Tecolote	T	ca. 75 m. *Global carbon isotope correlation remains uncertain in this interval, resulting in ongoing*		

	Formation		*uncertainty in the maximum and minimum ages of the Clemente, Pitiquito, Gamuza, Papalote and Tecolote formations.*		
	Papalote Formation	P	ca. 430 m. *Global carbon isotope correlation remains uncertain in this interval, resulting in ongoing uncertainty in the maximum and minimum ages of the Clemente, Pitiquito, Gamuza, Papalote and Tecolote formations.*		
	Gamuza Formation	GAM	ca. 115 m. *Global carbon isotope correlation remains uncertain in this interval, resulting in ongoing uncertainty in the maximum and minimum ages of the Clemente, Pitiquito, Gamuza, Papalote and Tecolote formations.*		
	Pitiquito Formation	PTQ	ca. 75 m. *Global carbon isotope correlation remains uncertain in this interval, resulting in ongoing uncertainty in the maximum and minimum ages of the Clemente, Pitiquito, Gamuza, Papalote and Tecolote formations.*		
	Clemente Formation		ca. 200 m. *Global carbon isotope correlation remains uncertain in this interval, resulting in ongoing uncertainty in the maximum and minimum ages of the Clemente, Pitiquito, Gamuza, Papalote and Tecolote formations.*		
Laurentia (California/Nevada, Great Basin)	Poleta Formation		ca. 550 m [after composite section of ref. (*185*)]		(*185*)

	Campito Formation		ca. 1.20 km [after composite section of ref. (*185*)]		(*185*)
	Lower Wood Canyon Formation and equivalent strata of the Deep Spring Formation	LWC/DS	Deep Spring Formation and equivalent lower Wood Canyon Formation [correlated via lithostratigraphy and carbon isotope chemostratigraphy in refs. (*30*, *65*)]: Interval thickens from SE to NW, with average thickness ca. 310 m (based on 12 sections).		(*30*, *65*)
	Reed Dolomite		ca. 500 m [based on simplified composite section of ref. (*65*)].		(*65*)
	Stirling Formation		ca. 950 m [based on simplified composite section of ref. (*65*)].		(*65*)
	Johnnie Formation		Shuram downturn and nadir within the Rainstorm Member, ca. 100 m (*50*, *183*). Full thickness of the Johnnie Formation is 600 m.		(*50*, *183*)
				Additional(/summary) references	(*30*, *49*, *50*, *65*, *123*, *135*, *171*, *173*, *180*, *183*, *185–192*)
Australia (Adelaide Superbasin, Flinders Ranges)	Hawker Group, Mernmerna Formation	MERNM	Average thickness uncertain. Minimum thickness ca. 60 m, maximum thickness ca. 850 m (*108*). Average thickness at Bunyeroo Gorge ca. 200 m, based on composite section of ref. (*193*).		(*108*, *193*)
	Hawker Group, Wirrapowie Limestone and Wilkawillina	WIR/WIL	Combined approximate composite thickness of successive segments of the Woodendinna Formation, Wirrapowie limestone and Wilkawillina limestone provide a maximum thickness of ca. 1.8		(*108*, *193*)

	Limestone		km [based on sections of ref. (*108*)]. Minimum thickness estimate of ca. 600 m for Woodendinna and overlying Wilkawillina limestone based on composite section of ref. (*193*).		
	Hawker Group, Woodendinna Formation / Woodendinna Dolomite	WOO			
	Hawker Group, Parachilna Formation	PARA	*ca. 50 m-thick* (*194*). *The maximum age of the basal Parachilna Formation is unknown.*		(*194*)
	Hawker Group, Uratanna Formation		*ca. 375 m-thick* (*194*). *Note that the maximum age of the Uratanna Formation remains unknown. Co-occurrence of frondose body fossils and T. pedum in upper Uratanna Formation implies that at least the upper Uratanna Formation is lower Cambrian in age, by current definition, with implication for range extension of soft-bodied biota into the lower Cambrian* (*194*). *Requires further investigation.*		(*194*)
	Wilpena Group, Pound Subgroup, Upper Rawnsley Quartzite	URQ	*Maximum thickness ca. 730 m based on composite section of the Chace Range* (*195*). *Note that the age of the boundary between the upper Rawnsley Quartzite and the overlying Uratanna Formation is unknown. Minimum age for the upper Rawnsley Quartzite remains unknown.*		(*195*)
	Wilpena Group, Pound Subgroup, Rawnsley Quartzite, Ediacara Member		*Average thickness ca. 190 m based on 11 measured sections in the Chace Range, Elder Range, Wilpena Pound and Heysen Range* (*195*). *Minimum and maximum ages for the Ediacara Member remain unknown. The temporal extent of this unit is based on tentative best-fit biostratigraphic correlation to the dated succession of the White Sea area, which*		(*195*)

			contains a comparable soft-bodied fossil assemblage.		
	Wilpena Group, Pound Subgroup, Chace Quartzite Member	CQ	*Average thickness ca. 85 m based on 7 measured sections in the Chace Range, Elder Range, Wilpena Pound and Heysen Range* (*195*)*. Minimum and maximum ages of the Chace Quartzite Member remain unknown.*		(*195*)
	Wilpena Group, Pound Subgroup, Bonney Sandstone	BO	*Maximum thickness ca. 400 m based on composite section of the Chace Range* (*195*)*. Minimum age of the Bonney Sandstone remains unknown.*		(*195*)
	Wilpena Group, Wonoka Formation		Total thickness ca. 800 m based on composite canyon fill section of ref. (*196*).		(*196*)
	Wilpena Group, Bunyeroo Formation		Ref. (*197*) note that the thickness of the Bunyeroo Formation above the Acraman impact ejecta layer is 4x thicker than the interval that underlies the ejecta layer (which itself is 80 m-thick). Uncertainty remains in the precise age of the Acraman impact, but it is widely considered to approximate the Gaskiers glaciation ca. 580 Ma. This interval of the Bunyeroo Formation is therefore tentatively suggested to be ca. 320 m-thick.		(*197*)
				Additional(/summary) references	(*108*, *195*, *196*, *198–202*)
White Sea Area, northwest Russia (East European Platform)	Padun Group, Brusov Formation		*Average thickness ca. 116 m. Poorly constrained maximum and minimum age, but assumed to be Cambrian Stage 2 based on associated fossil assemblage.*		

	Padun Group, Nyugus Formation	NYU	*Average thickness ca. 91 m. Poorly constrained maximum and minimum age.*		
	Padun Group, Zolotitsa Formation	ZOL	*Average thickness ca. 70 m. Poorly constrained maximum and minimum age.*		
	Erga/Yorga Formation	ERG	*Average thickness ca. 151 m. Minimum age for Erga/Yorga Formation remains uncertain.*		
	Zimnegory Formation	ZIM	Average thickness ca. 59 m		
	Verkhovkha Formation	VERKH	Average thickness ca. 150 m		
	Lyamtsa Formation		*Average thickness ca. 160 m. Maximum age for base of Lyamtsa Formation unconstrained.*		
				Additional(/summary) references	(*22*, *28*, *137*, *203–206*)
Moldova/Podilya succession, Moldova and southwest Ukraine (East European Platform)	Samets Formation (Самецька світа)		*Lower and upper boundaries of the Samets Formation are erosive. Thickness ca. 84 m* (*207*). *Uncertain age range as lower and upper boundaries are erosive and limited biostratigraphic information exists.*		(*207*)
	Baltic Group, Zhbruch Formation		*Total Group thickness (average ca. 130 m) appears to be relatively consistent across the region [*(*208*) *and references therein], but precise age range of*		(*208*)

	Batic Group, Khmielnitsky Formation	KHMI	*Group and individual formations remains highly uncertain.*		
	Baltic Group, Okunets Formation	OKU			
	Kanyliv Group: from stratigraphically lowest to highest: Danylivka Formation (Pylypy and Shebutyntsi beds) Zharnivka Formation (Ku and SU beds), Krushanivka Formation (Kryvchany and Du beds), and Studenytsya Formation (Po and Komariv beds)		*Kanyliv Group (ca. 172 m): Danylivka Formation (ca. 50 m), Zharnivka Formation (ca. 25 m), Krushanivka Formation (ca. 60 m), Studenytsya Formation (ca. 37 m). Precise age ranges of individual formations within the Kanyliv Group remain uncertain and controversial. However, the presence of Burykhia in the Studenytsya Formation* (*209*) *may suggest a slightly older correlation than shown for the upper Kanyliv Group [coincident with the Kuibis Subgroup of the Nama Group based on occurrence of Ausia fenestrata in Kliphoek Quartzite,* (*210*)*]. Given that the range extension of this fossil remains uncertain, and is known to extend into the Nama assemblage interval, using it for assigning a precise age is currently problematic.*		(*209*, *210*)
	Mohyliv-Podilsky Group, Nagoryany Formation (Szhuzhivka and Kalyus beds)	NAG	ca. 70 m. *Individual Member thicknesses are highly variable from Ivano-Frankivsk to Mohyliv Podilsky to Rivne [*(*208*) *and references therein].*		(*208*)
	Mohyliv-Podilsky Group, Yaryshiv Formation	YARY	ca. 55 m. *Individual Member thicknesses are highly variable from Ivano-Frankivsk to Mohyliv Podilsky to Rivne [*(*208*) *and references therein].*		(*208*)

	(Lyadova, Bernashivka Br and Zn beds)				
	Mohyliv-Podilsky Group, Mohyliv Formation (Olc, Lm and Yampil beds)	MOHYL	ca. 50 m. *Individual Member thicknesses are highly variable from Ivano-Frankivsk to Mohyliv Podilsky to Rivne [(208) and references therein].*		(*208*)
	Volyn Group, Hruska Formation		ca. 20 m		(*211*)
	Additional(/summary) references				(*131*, *207–209*, *211–213*)
Siberian Platform, NW Siberian Platform (e.g., Igarka-Norilsk Uplift)	Shumnoy Formation	SHUM	Minimum thickness ca. 30 m. *Uncertain minimum age for the Shumnoy Formation within this interval.*		(*214*, *215*)
	Krasny Porog Formation		ca. 35 m		
	Sukharikha Formation		ca. 135 m		
	Izluchin Formation	IZ	*ca. 18 m. Uncertain maximum age.*		
Siberian Platform, SW Siberian Platform (e.g., Yenisei Range)	Lebyazhino Formation		*Minimum thickness for this time interval ca. 620 m. Based on loose chemostratigraphic correlation* (*72*)		(*216*) Carbon isotope correlation follows (*72*)
	Nemchanka Formation		*ca. 3.2 km (assuming approximately continuous depositional rate and correlation of stromatolitic limestone interval with 1n/BACE). Poorly constrained due to dominantly siliciclastic lithology*		

			and lack/paucity of available radiometric, chemostratigraphic or biostratigraphic markers.		
Siberian Platform, S Siberian Platform (e.g., Irkutsk Amphitheatre)	Olekma Formation	OLEK	Maximum thickness in carbonate-dominated sections ca. 165 m		
	Tolbachan Formation		Maximum thickness in carbonate-dominated sections ca. 450 m		
	El'gyan Formation		Maximum thickness in carbonate-dominated sections ca. 320 m		
	Nokhtuysk Formation	NO	ca. 520 m	*Magnitude and trend of carbon isotope data reported from the Nokhtuysk Formation by ref.* (*217*) *matches well with the 1n/BACE, but interpreted therein as SHICE. Requires future verification.*	(*217*)
	Tinnaya Formation	TINNA	ca. 330 m		
	Zherba Formation		ca. 350 m. *Limited data to accurately constrain the maximum age of the Zherba Formation.*		
	Zhuya Group, Chencha Formation	CHEN	Maximum thickness ca. 720 m		

	Zhuya Group, Nikol’skoe Formation	NIKOL’	ca. 500 m		
	Dal’nyaya Taiga Group, Valyukhta Formation		*Maximum thickness in this interval ca. 990 m [Ura Uplift section, (218, 219)]. Limited data to accurately constrain maximum age of Valyukhta Formation.*		(*218*, *219*)
Siberian Platform, central Siberian Platform (e.g., Sygdzhera Saddle - Nepa-Botuoba Uplift to southern slope of the Anabar Shield)	Olekma Formation	OLEK	ca. 100 m		(*220*), following litho- and chemo-stratigraphic correlations outlined in (*72*)
	Tolbachan Formation		Maximum thickness ca. 450 m		
	Yurega Formation	YU	*ca. 220 m*		
	Danilovo Group, including Uspun, Kudulakh, Yuryakh and Bilir formations	USP/KUD/ YUR/BILIR	*ca. 400 m*		
	Tira Group, Byuk Formation		*Upper Byuk Formation: Maximum thickness ca. 200 m*		
	Nepa Group, Parshino Formation		*Lower siliciclastic interval ca. 150 m (assuming similar sedimentation rate to overlying carbonate interval that records ‘Shuram recovery’),* carbonate interval of upper Parshino + lower Byuk Formation recording the Shuram recovery ca. 50 m.		
Siberian Platform, SE Siberian Platform	Titary Formation	T	ca. 62 m		(*55*, *221*).

(Uchur-Maya Plate to Yudoma-Maya Belt)	Kutorgina and Keteme formations	K-K	Kutorgina Formation ca. 115 m. Keteme Formation ca. 165 m.		Compiled references in (*55*, *72*)
	Sinsk Formation	S	ca. 76 m		
	Perekhod Formation	PK	ca. 60 m		
	Pestrotsvet Formation		Composite Lena River section thickness (plus lowermost interval at Selinde River section inferred to record peak '6p') ca. 280 m.		
	Ust'-Yudoma Formation	U'YUDO	Maximum thickness in most continuous single section (Kyra-Ytyga) ca. 280 m. *However, note that dolostone of the Ust'-Yudoma Formation at this section may only represent the upper Ust'-Yudoma Formation relative to laterally equivalent sections (e.g., Nuuchchalakh Valley) due to lateral progradation of the carbonate platform [discussion in ref.* (*72*)*]. Remains poorly constrained in time due to difficulty in robust lateral chemo- and biostratigraphic correlation.*		(*72*, *222*). Full references in (*72*)
	Aim Formation		Clastic-dominated lower Aim Formation ca. 50 m, carbonate-dominated upper Aim Formation average thickness ca. 65 m. *Remains poorly constrained in time due to difficulty in robust lateral chemo- and biostratigraphic correlation.*		
Siberian Platform, NE Siberian Platform (e.g., Olenek Uplift and Khara-Ulakh Mountains)	Erkeket Formation		Minimum thickness ca. 190 m		
	Kessyuse Group, Chuskuna	C	Chuskuna Formation (ca. 26 m)		

	Formation				
	Kessyuse Group, Mattaia Formation	MAT	Mattaia Formation (ca. 50 m),		
	Kessyuse Group, Syhargalakh Formation	S	Syhargalakh Formation (ca. 27 m)		
	Turkut Formation	TURK	*ca. 65 m. Precise age of Khatyspyt/Turkut boundary remains uncertain.*		
	Khatyspyt Formation		*ca. 195 m. Precise ages of Maastakh/Khatyspyt and Khatyspyt/Turkut boundaries remain uncertain [<550 Ma after ref.* (*223*)*].*		(*223*)
Additional(/summary) references					Full compiled reference lists and lithostratigraphic correlation frameworks are compiled in the main text and supplementary information files of (*72*) and (*55*)
Oman (South Oman Salt Basin, Huqf, and Oman Mountains/Jabal Akhdar area)	Mahatta Humaid Group, including Amin, Mahwis/Miqrat and Andam formations		Only the lower Mahatta Humaid Group may be present in the studied time interval. *Thickness of Amin (max. 700 m) and Mahwis/Miqrat (ca. over 350 m) formations* (*224*). *Uncertain temporal extent.*		(*224*)

	Nimr Group, including Karim and Haradh formations	NIM	*Approximate thickness ca. 435 m.*		
	Ara Group, including Birba, "U"/Athel, Al Noor and Dhahaban formations and lateral equivalent volcanics of Jabal Akhdar		*Extreme differences in lateral thickness associated with differential thickness of evaporite units and carbonate stringers. Maximum thickness estimated at ca. 600 m. See ref.* (*30*) *for details of alternative age for negative carbonate carbon isotope excursion recorded within A4 carbonate stringer (alignment with the 1n/BACE with onset at ca. 535 Ma). This re-alignment is used herein, and contrasts with age models that anchor this excursion to a radiometric age (ca. 541 Ma) from an ash bed at base of A4 carbonate stringer [see ref.* (*124*)*, and now outdated carbon isotope age models of ref.* (*94*)*].*		(*30*, *94*, *124*)
	Buah Formation		*Approximate maximum thickness ca. 250 m. The Buah Formation appears to be thicker in the TM-6 core than elsewhere.*		
	Shuram Formation		Maximum thickness ca. 380 m		
	Khufai Formation		Scaled vertical thickness of ca. 310 m (*225*)		(*226*)
	Masirah Bay Formation	M	*Thickness of uppermost Masirah Bay Formation siliciclastics that cover BAINCE interval uncertain. This interval is assigned ca. 20 m, which is likely near a maximum possible thickness based on average sedimentation rates throughout the overlying and underlying SOSB succession.*		

	Additional(/summary) references				(*30*, *135*, *226–234*)
Brazil	Corumbá Group, Guaicurus Formation		*ca. 180 m. Uncertainty remains in precise maximum and minimum ages of the Guaicurus Formation.*		
	Corumbá Group, Tamengo Formation		*Max. thickness estimated at ca. 140 m. There remain issues in confident lateral correlation of sections and differential section completeness that result in uncertainty in maximum and minimum ages of the Tamengo Formation.*		
	Corumbá Group, Bocaina Formation		*ca. 40-100 m. Maximum and minimum ages of Bocaina Formation remain uncertain. This age model assumes notable hiatus at the Bocaina/Tamengo boundary. The upper Bocaina Formation has very limited carbon isotope data to aid chemostratigraphic correlation, but recently reported carbon isotope data from the lower to middle Bocaina Formation by ref.* (*92*) *in combination with stratigraphic correlations of outcrop and drill core* (*93*)*, aid correlation.*		(*235*, *236*)
	Corumbá Group, Cerradinho Formation	CER	*ca. 10 m. Uncertain age for the Cerradinho Formation.*		
	Additional(/summary) references				(*89*, *91*, *237*, *238*)
Southern Namibia and northwest South Africa (Kalahari Craton)	Nama Group, Fish River Subgroup	FR	*Fish River Subgroup (total outcrop area ca. 37,800 km^2): Average thickness estimated ca. 800 m. Uncertain temporal range within lower Cambrian.*		

Unit thicknesses:<br>Units pinch-out in proximity to the Kalahari craton to the east, and various basement arches such as 'Osis' and 'Koedoelaagte'	Nama Group, Schwarzrand Subgroup, upper Nomtsas Formation	UN	Herein prescribed to strata of the Nomtsas Formation that overlie the erosive unconformity in some sections of the Witputs Sub-basin [not present on the Neint Nababeep Plateau, (*63*)]. Nomtsas Formation (total outcrop area ca. 15,250 km^2): *upper Nomtsas Formation (above erosion surface in eastern sections) average thickness ca. 24 m. Poor age control on upper Nomtsas Formation, but assumed to be approximately correlative in age with basal Fortunian clastics on other continents [equal to or younger than peak 1.5p/2p based on preliminary global biostratigraphic correlation with successions that host* T. pedum*, e.g., ref.* (*30*)].		(*30*, *125*)
	Nama Group, Schwarzrand Subgroup, lower Nomtsas Formation	LN	Herein prescribed to strata of the Nomtsas Formation below the erosive unconformity (corresponding to, at minimum, the Nomtsas Formation of the Neint Nababeep Plateau). Nomtsas Formation (total outcrop area ca. 15,250 km^2): In Witputs Sub-basin lower Nomtsas Formation average thickness ca. 118 m.		
	Nama Group, Schwarzrand Subgroup, Urusis Formation, including (in ascending order) the Nasep, Huns, Feldschuhhorn and Spitskop members		Urusis Formation (total outcrop area ca. 16,250 km^2): In Witputs Sub-basin [including Neint Nababeep Plateau (*63*) and Tses borehole (*239*)]: Urusis Formation average thickness ca. 450 m. Note ongoing uncertainty in lateral correlation of the Urusis Formation between the Witputs and Vioolsdrif sub-basins [e.g., discussed briefly in ref. (*125*)].		(*125*)
	Nama Group, Schwarzrand Subgroup, Nudaus	NUD	Nudaus Formation (total outcrop area ca. 16,650 km^2): average thickness ca. 250 m		

	Formation, including (in ascending order) the Niederhagen and Vingerbreek members				
	Nama Group, Kuibis Subgroup, Zaris Formation, including the Mooifontein Member (Witputs Sub-basin) and, in ascending order, the Omkyk, Hoogland and Urikos members (Zaris Sub-basin)	ZA	Zaris Formation (total outcrop area including exposure in the Fish River Canyon, ca. 33,000 km^2): Mooifontein+upperOmkyk+Hoogland+Urikos members (average thickness ca. 140 m). Note that Zaris Sub-basin thicknesses greatly exceed Witputs Sub-basin thicknesses for the Zaris Formation.		
	Nama Group, Kuibis Subgroup, Dabis Formation, including the Kanies Member (Zaris and Witputs Sub-basins), and the Mara, Kliphoek and Aar members (Witputs Sub-basin only)	DA	Dabis Formation (total outcrop area including exposure in the Fish River Canyon, ca. 25,000 km^2): Kanies Member (average thickness ca. 18 m), Mara Member (average thickness ca. 84 m), Kliphoek+Aar+lowerOmkyk(Zaris Formation equivalent horizon) members (average thickness ca. 75 m). *Age of the base of the Nama Group remains uncertain.*		
	Additional(/summary) references				(*63*, *240–244*) and compiled references in (*18*)

Anti-Atlas Mountains, Morocco<br><br>Unit thicknesses:<br>Highly variable thickness and completeness across southern Morocco (verified by radiometric and chemostratigraphic data)	Tazlaft Formation	TAZ	*Max. thickness ca. 100 m. Uncertain average thickness and/or temporal extent.*		
	Issafen Formation	ISSAF	Approximate total thickness where fully developed ca. 175 m. *Uncertain minimum age*.		
	Amouslek Formation	A	Approximate total thickness where fully developed ca. 250 m		
	Igoudine Formation	I	Approximate total thickness where fully developed ca. 165 m		
	Lie de Vin Formation	LDV	Approximate total thickness where fully developed ca. 900 m		
	Adoudou Formation		Approximate total thickness where fully developed ca. 1.15 km		
	Additional(/summary) references				(*109*, *111–113*, *245*, *246*)
Mongolia, Zavkhan Terrane	Khairkhan Formation		*Up to 200 m thick at Salaa Gorge. Precise temporal placement uncertain but biostratigraphically constrained to be equivalent to Botoman-Toyonian* (*247*)		(*247*)
	Salaagol Formation		*Average thickness where fully developed ca. 180 m. Precise temporal placement remains uncertain [see discussion and corresponding references in refs.* (*94*, *248*)].		(*94*, *248*)
	Bayangol		Average thickness where fully developed ca. 800 m		

	Formation				
	Zuun-Arts Formation	Z-ARTS	Average thickness where developed ca. 200 m		
				Additional(/summary) references	(*64*, *189*, *247–249*)

*Geologic Time Scale 2020 used as universal reference text throughout, except where superseded by more recent published information.

Additional external supplementary datasets associated with this article:

Data S1: Age framework database (580–510 Ma), including temporally calibrated carbonate carbon and uranium isotopes from multiple sources, paleontological occurrence information and biostratigraphic justifications, with full references [updated after ref. (*18*)].

Data S2: Age framework database (580–510 Ma) following Data S1, in a software-readable format.

Data S3: Lithostratigraphic database (580–510 Ma) detailing hiatuses and dominant lithofacies for 24 composite stratigraphic sections subdivided into 50 Kyr increments. Dominant lithofacies are classified as non-marine/restricted/diamictite (including volcanic; diamictite; evaporitic/semi-restricted; fluvial/continental siliciclastic), and marine, including siliciclastic (shallow inner shelf, mid-outer shelf to upper slope; lower slope to basinal), carbonate (shallow inner shelf; mid-outer shelf to upper slope; lower slope to basinal), and phosphorite.

Data S4: Genus-level turnover per-capita rates (*82*) and diversity at 1 Myr resolution [divDyn R package of ref. (*81*)] that incorporate and test biostratigraphic uncertainties of the age framework.
